# Electroreduction of Activated Esters Catalyzed by a Manganese Complex

**DOI:** 10.1021/acs.organomet.6c00205

**Published:** 2026-08-14

**Authors:** Kai-Thorben Kuessner, Nils Rotthowe, Max C. Holthausen, Nilay Hazari, Alexander J.M. Miller, Inke Siewert

**Affiliations:** † Georg-August-Universität Göttingen, 9375 Institut für Anorganische Chemie, Tammannstr. 4, Göttingen 37077, Germany; ‡ Institut für Anorganische und Analytische Chemie, 9173 Goethe-Universität, Max-von-Laue-Strasse 7, Frankfurt Am Main 60438, Germany; § Department of Chemistry, 5755 Yale University, P.O. Box 208107, New Haven, Connecticut 06520, United States; ∥ Department of Chemistry, 2331 University of North Carolina at Chapel Hill, Chapel Hill, North Carolina 27599, United States; ⊥ Georg-August-Universität Göttingen, International Center for Advanced Studies of Energy Conversion, Tammannstr. 6, Göttingen 37077, Germany

## Abstract

Electrochemical hydrogenation (electrohydrogenation) using electrons and protons as H_2_ surrogates offers a sustainable alternative to conventional reduction methods. Herein, we report the manganese-catalyzed electroreduction of activated esters employing a derivative of Mn­(bpy)­(CO)_3_ (bpy = 2,2’-bipyridine) featuring a proton relay in the ligand. Using low catalyst loadings (1 mol%), a range of fluorinated esters were reduced to the corresponding alcohols under mild electrochemical conditions. Experimental and quantum-chemical mechanistic studies provide an understanding of the reaction pathway. The key catalytic intermediate, a manganese hydride species, is generated by electrochemical reduction of the precatalyst followed by protonation. Computational analysis indicates that hydrogenation proceeds via an outer-sphere H^–^/H^+^ transfer pathway. These findings expand the scope of base-metal electrocatalytic hydrogenation to esters and provide insight into manganese-hydride-mediated electroreduction mechanisms.

## Graphical abstract

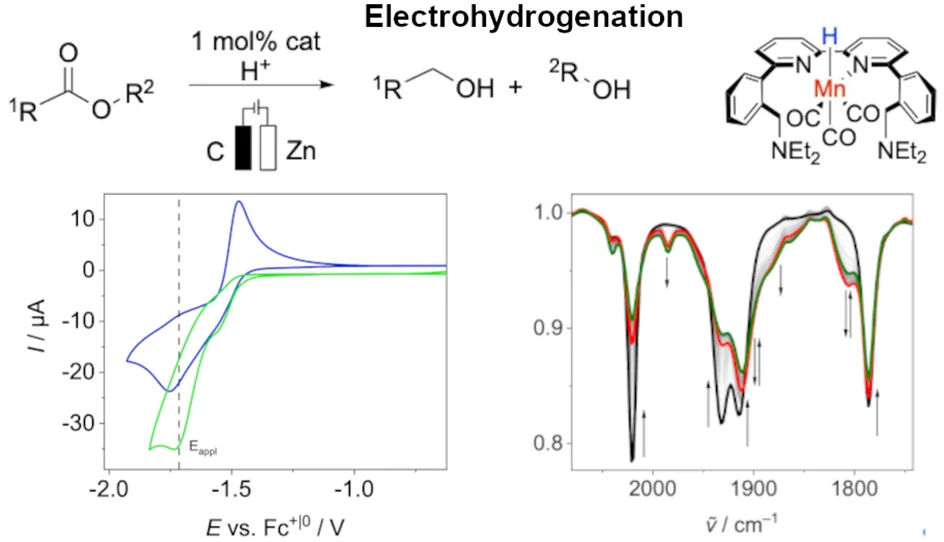

## Introduction

3d transition metal hydrides are crucial intermediates in a large number of chemical transformations ranging from the hydrogen evolution reaction,^1^ to olefin isomerization,^2^ and hydrogenation reactions.^3^ 3d metal hydride species usually feature relatively low MH bond dissociation free energies (BDFE) of around 50–60 kcal mol^–1^ and thus can be prone to H_2_ loss.^4^ Further, they span a large range of thermodynamic hydricities, i.e., the free energy for hydride ion donation, and react with electrophiles.^5^ The ability of 3d metal hydride species to donate a hydride ion or a hydrogen atom has been exploited widely in chemical catalysis for the hydrogenation of π-systems such as alkenes, ketones, esters, and nitriles.^3^ In the vast majority of these protocols, dihydrogen or alcohols are used as the hydrogen source.^3,6^ Recently, there have been a small number of reports where electrons and protons serve as H_2_ surrogates in such 3d transition metal-catalyzed hydrogenation reactions, although the electrochemical formation of the crucial M–H species is widely explored in reactions related to energy storage.^7^ In the hydrogen evolution reaction (HER), the molecular MH species is commonly formed by 2e^–^ reduction and subsequent protonation of the metal complex. The MH species then reacts with protons to form H_2_ (Scheme 1b). In turn, the HER represents an undesired side reaction in electrochemical protocols targeting π-systems in organic scaffolds and thus must be suppressed kinetically or thermodynamically. The thermodynamic bias toward the desired reaction—expressed by the standard potential difference between electrochemical H_2_ formation and electroreduction of the organic scaffolds—is only small (Scheme 1a).^8^

**Scheme 1:**
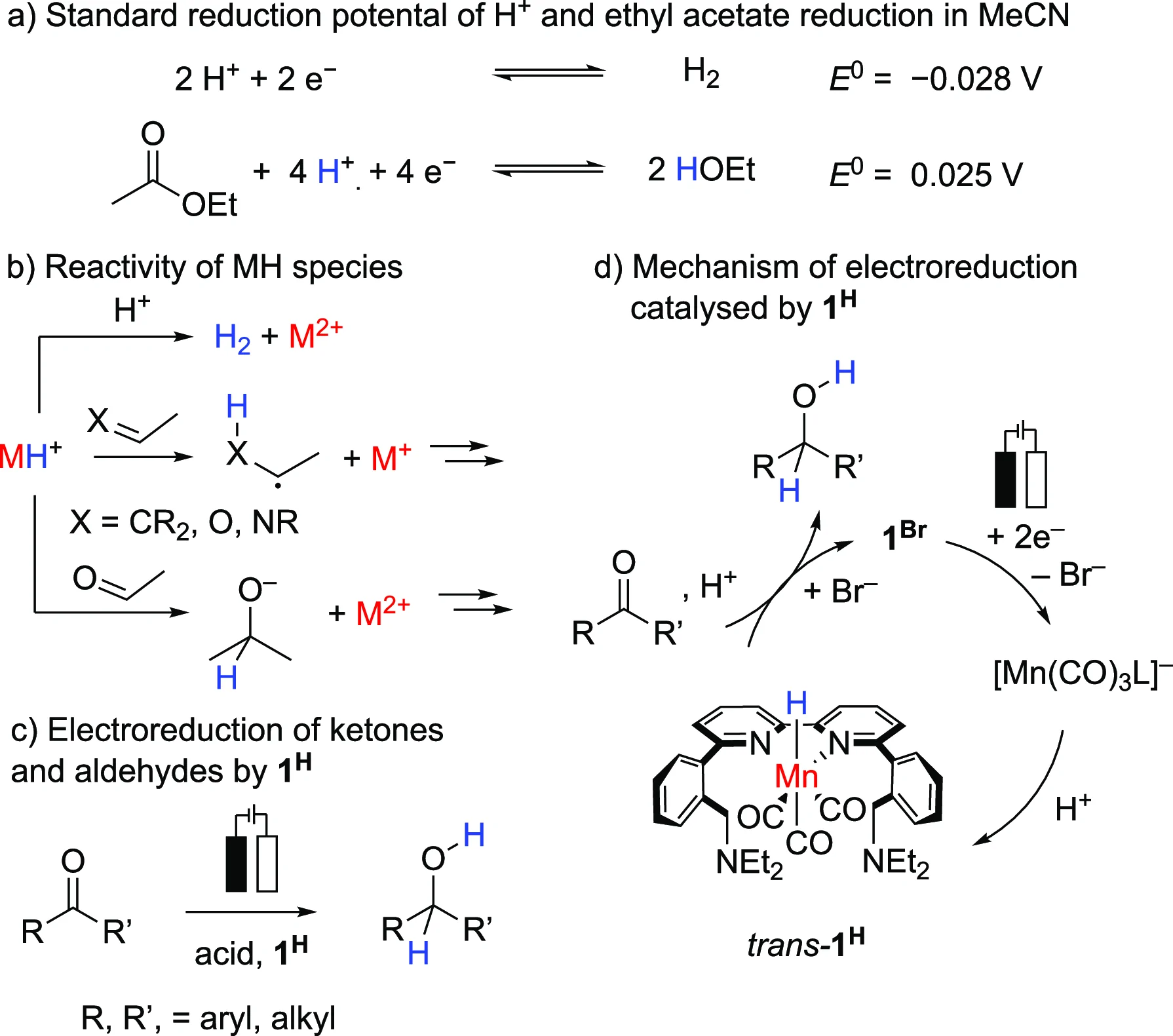
a) Standard Reduction Potentials in MeCN *vs.* Fc^+|0^; b) Schematic Reaction of Metal Hydride Complexes with Electrophiles; c, d) Ionic H^+^/H^–^ Electroreductions of Ketones. **1** Denotes the {Mn^I^(CO)_3_L}-Core Motif and the Superscript Denotes the Sixth Ligand

Ionic electrohydrogenations, which proceed via a formal H^–^/H^+^ transfer, provide a suitable strategy for selective polar C=O double bond reductions over thermodynamically favored C=C bond hydrogenations (Scheme 1b).^9^ Inspired by the work on the electrohydrogenation of CO_2_ to form formic acid by manganese tricarbonyl complexes,^10^ we recently reported on base-metal-catalyzed protocols for the selective electroreduction of polar C=O bonds over C=C bonds in ketones and aldehydes using electrons and protons forming the alcohols (Scheme 1c).^7b,7e^ The reaction is applicable to a large variety of aliphatic and aromatic ketones and aldehydes and proceeds with excellent Faraday efficiencies (FE) and yields up to 99%.

Mechanistic studies revealed that **1** ^**H**^, [MnH­(CO)_3_L] (L = 2,2’ functionalized bipyridine), is formed from **1** ^**Br**^, [Mn­(CO)_3_LBr], in a stepwise EC^Br^EC^H+^ mechanism consisting of two reduction steps, which are connected by bromide loss in between,^7e,10b,11^ and subsequent protonation of the highly nucleophilic [Mn­(CO)_3_L]^−^ species (Scheme 1d).^10a,10b^ Amine groups attached to the bipyridine ligands that can act as proton relays were found to be crucial to achieve reasonable reaction rates for the protonation of [Mn­(CO)_3_L]^−^.^10b,12^ A quantum-chemical study on the electrohydrogenation of CO_2_ by [Mn­(CO)_3_L]^−^ demonstrated that the proton relay acts as a base effectively enabling protonation of the nucleophilic metal center forming [MnH­(CO)_3_L].^10b^ Trifluoroethanol (TFE) is bound to the amine functionality via hydrogen bonding, which lowers the activation barrier for **1** ^**H**^ formation by 12 kcal mol^–1^ and, in turn, makes this step accessible at rt.^10b^ Further, ab initio molecular dynamics (AIMD) simulations and DFT calculations on [Mn­(CO)_3_L]^−^ showed that the amine units are flexible and both *trans* and *cis* conformations are feasible.^12^ We denote the *cis* conformer as the isomer in which both amine groups are in the same hemisphere as the reactive site, while in the *trans* conformer, both amine groups are in the same hemisphere as the axial CO ligand (Scheme 1d). At an applied potential of −1.7 V (all potentials in the paper are given vs. Fc^+|0^), **1** ^**H**^ and TFE react in a H^+^/H^–^ transfer pathway with the ketone to generate the alcohol product.^7e^ It was unclear if this reaction proceeded via an outer-sphere H^+^/H^–^ mechanism or in a two-step inner-sphere process forming the manganese alkoxide species, which is protonated subsequently.

So far, successful base-metal-catalyzed electrohydrogenation reactions have only been described for aldehydes, ketones, alkenes, or alkynes, whereas examples with esters have not been reported.^7,13^ Esters represent an attractive target because they are ubiquitous functional groups in sustainable chemical feedstocks, such as vegetable oils, and in the chemical industry for producing fragrances, flavors, and other fine chemicals.^14^ The reduction of carboxylic acids and esters is likely more challenging because the carbonyl carbon atom has a lower electrophilicity compared with ketones or aldehydes. Here, we show that introducing electron-withdrawing groups into the ester enables the application of **1** ^**Br**^ as a versatile electrocatalyst for reduction of esters using only 1 mol% catalyst loading. The protocol can be applied to a range of activated esters. Furthermore, in-depth experimental and quantum chemical studies were conducted to elucidate mechanistic details of the hydrogenation step and aid in the design of improved catalysts.

## Results and Discussion

Our previous study demonstrated^7e^ that nonactivated esters are not hydrogenated using **1** ^**Br**^. Therefore, we pursued an approach involving activating the carbonyl group using an electron-withdrawing group. Thus, we chose ethyl 2,2,2-trifluoroacetate **2a** as a model ester substrate with an activated electrophilic C atom. As the starting point, we utilized the same conditions as previously reported for ketone reduction, namely, a glassy carbon (GC) foam electrode as the working electrode, 1 mol% **1** ^**Br**^, 100 mM of **2a**, and 2 M TFE as the acid, and conducted a controlled potential electrolysis experiment at −1.70 V in a separated cell (Table 1, entry 1). As the counter electrode, we utilized a zinc spiral electrode. After the injection of 4.5 charge equivalents with respect to the substrate **2a**, we indeed observed the formation of ethanol.

**Table 1:**
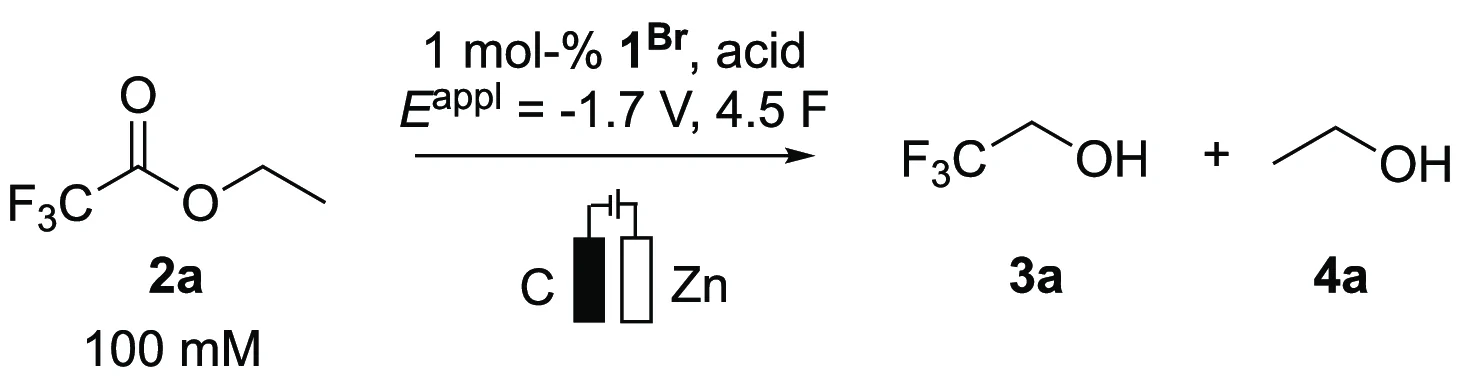
Optimization of the Reaction Conditions^a^

	% **3a**	% **4a**	Conv. %	acid	FE %	
1	–	70	85	2 M TFE	62	
2	50	90	90	2 M PhOH	40	
3	^b^	^b^	>99	1 M PhOH	^b^	
4	low	low	12	1 M Phos^c^	low	
5	58	85	87	1 M PhOH	49	^d^
6	66	82	>99	1 M PhOH	58	^e^
7	0	0	3	1 M PhOH	0	^e^ ^f^

a All experiments in MeCN as solvent.

b Reliable yields cannot be given due to precipitation.

c Phos: [^*t*^Bu-*N*H=P­{NP­(pyrrolidine)_3_}_3_]­[PF_6_].

d 0.2 M cyclam (1,4,8,11-tetraazacyclotetradecane) in the anodic chamber.

e 0.5 M ^n^Bu_4_NBr in the anodic chamber.

f Without **1** ^**Br**^.

Next, we optimized the reaction conditions. Switching to phenol as the acid allowed the use of a lower acid concentration of 1 M (Table 1, entries 2 and 3). The change in acid also allowed direct detection of trifluoroethanol alongside ethanol, confirming electroreduction of the ester. As a minor side product, we observe the formation of trifluoroacetic acid and/or its conjugate base, **5a**, during electrolysis, irrespective of the proton source. As this only occurs under applied potential, we attribute this to the basic conditions upon proton consumption and trifluoroethoxide formation during catalysis, which leads to the saponification of the ester. The precipitation of Zn^2+^ as poorly soluble ionic salts in the anodic chamber hampers charge flow, and thus 0.2 M cyclam (Table 1, entry 5) or 0.5 M ^n^Bu_4_NBr (Table 1, entry 6) was added to the anodic chamber. Zn^2+^ ions partially dissolve as its bromide salt in the latter case, resulting in an increased yield. The Faraday efficiency is similar when TFE and phenol were used; however, since the product **3a** could not be quantified when it was also used as an acid and since the acid loading was lower with PhOH, subsequent experiments were performed with PhOH.^15^

Using our optimized reaction conditions, we investigated the substrate scope of the reaction (see Figure 1). At first, we varied the number of F atoms at the CF_3_ substituent to evaluate the required degree of activation. Ethyl 2,2-difluoroacetate, **2b**, was still converted with a yield of 71% in **4a**, whereas no conversion was observed for ethyl 2-fluoroacetate, **2c**. Thus, considerable activation is necessary to achieve a high conversion. Next, we utilized esters **2d** and **2e**, with fluorine-containing groups in the alcohol moiety of the ester. For **2d**, considerable charge was injected, but no product formation was observed. Instead, transesterification was observed, forming **6d** and H_2_, which we attribute to the proton consumption during electrolysis resulting in basic conditions, which promote transesterification. **2e** decomposes during electrolysis, and no formation of any alcohol products was observed. These results suggest that the introduction of electron-withdrawing groups at the OR^2^ substituent of the ester does not lead to sufficient electrophilicity at the C atom to promote ester electroreduction.

**Figure 1:**
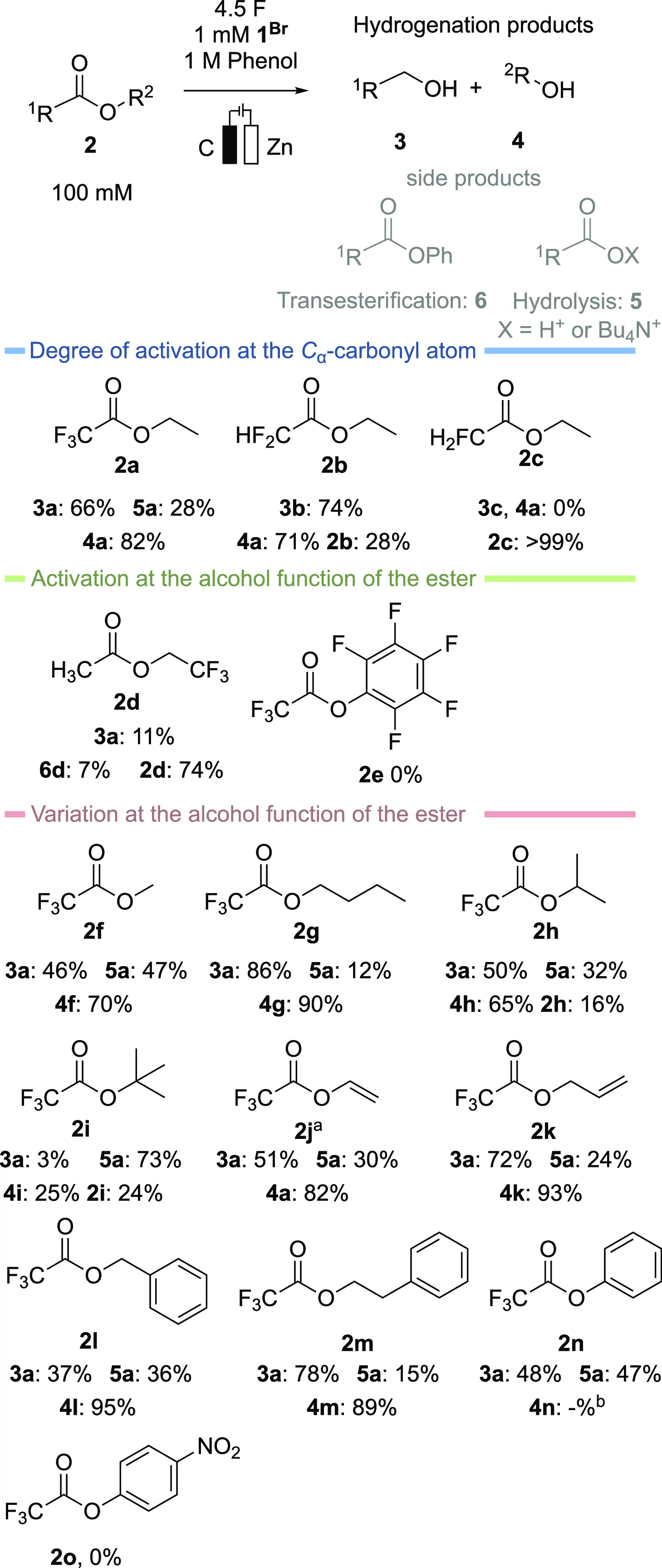
Scope of the electroreduction reaction using the optimized conditions; a: 6.5 charge equivalents were injected. b: Since phenol is used as the acid, the yield of **4n** could not be quantified.

Next, we varied the substituent at the OR^2^ moiety of the 2,2,2-trifluoroacetate ester. Increasing or decreasing the chain length of the group as in **2f** and **2g** leads to similar yields of the products. However, the yield decreased dramatically with increasing steric bulk (**2h** and **2i**). For **2j**, 6.5 charge equivalents were needed to observe full conversion, because the 4e^–^/4H^+^ reduced product, *viz*., the vinyl alcohol, isomerizes to acetaldehyde, which is further reduced at the applied potential, yielding ethanol.

Catalysis is selective for C=O bonds over C=C bonds, even though hydrogenation of the latter is thermodynamically favored: **2k** was converted in good yields, and the resulting alcohol retains its double bond and does not isomerize. Also, aromatic residues attached to the alkyl chain led to moderate-to-good conversions in the electroreductions, as shown for **2l** and **2m**. Finally, we investigated phenyl derivatives of trifluoroacetate: **2n** was converted in moderate yields, whereas the presence of a nitro group in the *para* position of the phenyl moiety led to unselective, noncatalyzed electroreduction of **2o**.

Subsequently, we explored the mechanism of the reaction. Labeling experiments confirmed electroreduction of the ester: After electrolysis using **1** ^**Br**^, **2a**, and phenol-d_6_ as the acid, the two deuterium atoms at the former carbonyl C atom in **2a** give rise to a ^2^H NMR signal at 3.85 ppm, confirming the formation of the reduced, deuterated alcohol CF_3_CD_2_OD, **3a**-d_3_ (Figure 2 and Figure S12). Electrochemical studies employing **1** ^**Br**^ confirmed the previously reported redox behavior of the complex.^10b^ **1** ^**Br**^ is reduced twice in an EC^Br^E type-mechanism at *E*_p_ = −1.75 V (ν = 0.1 V s^–1^), that is, initial reduction induces bromide loss and the resulting radical species [Mn­(CO)_3_L] is easier to reduce than **1** ^**Br**^, and thus, it is further reduced at the electrode surface forming [Mn­(CO)_3_L]^−^. [Mn­(CO)_3_L]^−^ is reoxidized at −1.56 V (Figure 2). Upon adding the acid and **2a**, the reoxidation peak of [Mn­(CO)_3_L]^−^ vanishes, because [Mn­(CO)_3_L]^−^ is protonated by the acid forming [Mn^I^H­(CO)_3_L], which is the catalytically active species (Figure 2, Figure S1).^10b^ Notably, we observe only very modest current enhancement at the initial reduction potential of −1.75 V under catalytic conditions on the time scale of the CV (ν = 0.1 V s^–1^), but electrolysis at that applied potential is successful, which confirms the previous observation that [Mn^I^H­(CO)_3_L] is catalytically active in electrohydrogenation reactions, though at slow rates.^7e,10b^ At more negative potentials of about −2.2 V, a new catalytic wave appears that belongs to the reduction of [Mn^I^H­(CO)_3_L] forming [Mn^0^H­(CO)_3_L]^−^, which is also catalytically active in the electrohydrogenation as shown previously (Figure S1).^7e,10b^ The current increase at ∼−2.2 V suggests faster catalysis than at −1.7 V. However, the harsh reductive conditions would have limited the substrate scope, and thus, catalysis was run at −1.7 V.

**Figure 2:**
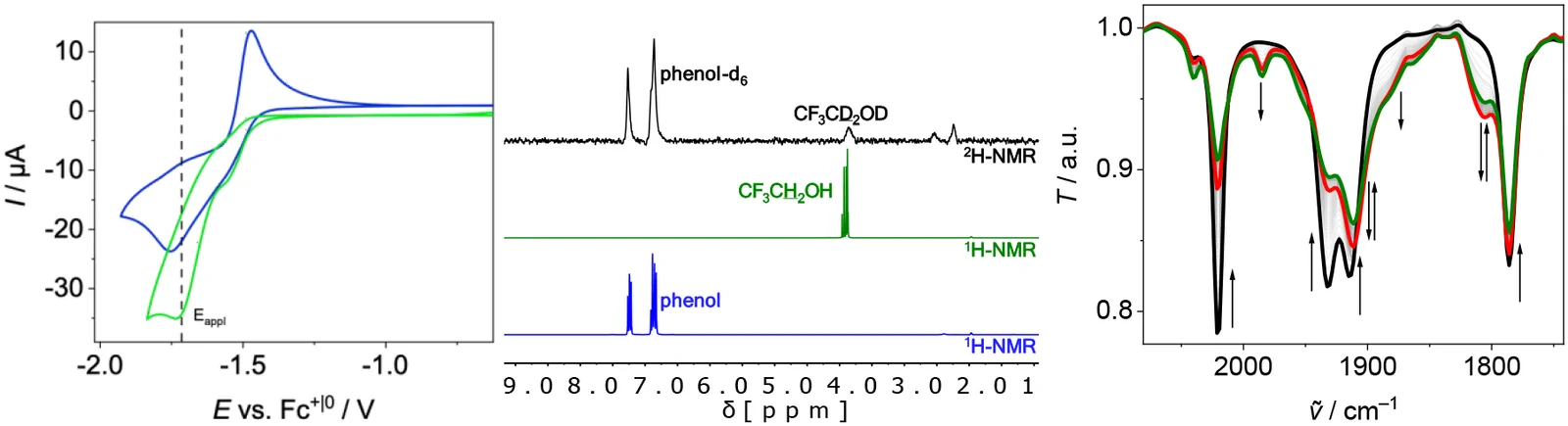
Left: CV data of **1^Br^** (1 mM, blue) and of **1^Br^** (1 mM) with PhOH (0.2 M) and **2a** (0.1 M, bright green) in MeCN, rt, 0.1 M NBu_4_PF_6_. Middle: Overlay of the ^2^H NMR spectrum (black) of the crude reaction mixture after the CPE experiment of **2a**, phenol-d_6_, and **1^Br^** in MeCN, *E*_appl_ = −1.7 V vs Fc^+|0^ and the ^1^H NMR spectra of TFE (green) and PhOH (blue); right: linear sweep IR-SEC of **1^Br^** (3 mM) in the presence of 10 equiv of TFE and 5 equiv of **2a** in MeCN, rt, 0.1 M NBu_4_PF_6_; black: beginning; red: after initial reduction; green: under catalytic conditions.

Subsequently, we conducted IR-spectroelectrochemical (IR-SEC) studies of **1** ^**Br**^, 30 mM TFE, and 15 mM **2a** to substantiate the active species during catalysis. **1** ^**Br**^ exhibits three carbonyl stretching vibrations at 2021, 1932, and 1915 cm^–1^,^10b^ which decrease upon applying a reductive potential. Concurrently, three new species are formed: one major species with stretching vibrations at 1912 and 1806 cm^–1^, and two minor species, one having characteristic stretching vibrations at 1985 cm^–1^, 1880 cm^–1^, and one at 2039 cm^–1^ (Figure 2 and Figure S3). Further bands of the minor species are likely masked by the main species. The main species was assigned to [Mn­(CO)_3_L]^−^ and the latter minor species tentatively to [Mn­(CO)_3_L­(MeCN)]^+^, **1** ^**MeCN+**^, based on previous reports.^10b,16^ **1** ^**MeCN+**^ is formed as the initial reaction product after one turnover. The minor species with characteristic stretching vibrations at 1985 cm^–1^ and at 1880 cm^–1^ was tentatively assigned to **1** ^**H**^ and/or [Mn­(CO)_3_L] based on previous reports.^7e,10,16^ Importantly, the C=O stretching vibration of **2a** at 1786 cm^–1^ decreases only after formation of [Mn­(CO)_3_L]^−^ is observed. In other words, electrochemical 2e^–^ reduction of **1** ^**Br**^ induces catalysis and leads to the formation of [Mn­(CO)_3_L]^−^, which reacts with the acid forming **1** ^**H**^. After catalytic turnover, [Mn­(CO)_3_L­(MeCN)]^+^, **1** ^**MeCN+**^, is formed.

This scenario was further substantiated by ^1^H NMR studies. Chemical reduction of **1** ^**Br**^ and subsequent protonation by phenol as reported previously lead to the formation of diamagnetic **1** ^**H**^, which has a characteristic ^1^H NMR signal at −3.1 ppm (Figure S10).^10b^ Upon addition of 100 equiv of **2a** to the solution of **1** ^**H**^, the signal of the hydride species vanishes in the ^1^H NMR spectrum, and the signal of the fluorinated alcohol **3a** appears in the ^19^F NMR spectrum taken after 30 min, confirming that the hydride species reduces the ester (Figure S11). Since the 2,2,2-trifluoroacetaldehyde, which is formed as the initial 2e^–^/2H^+^ reduced product after hydrogenation of **2a**, is much more reactive than the ester, it is reduced in a second hydrogenation cycle forming the alcohol.

We assume that the electrohydrogenation occurs via hydride/proton transfer step(s), because (i) C=C double bonds are not hydrogenated, (ii) we do not observe the radical coupling products, *viz*., biacetyl and butane-2,3-diol, and (iii) **1** ^**H**^ is a closed-shell species. However, as in the previous study,^7e^ it remained unclear whether this process occurs stepwise through a discrete alkoxide intermediate, similar to that proposed for the CO_2_ reduction employing **1** **H** ^10b^ or in an outer-sphere concerted transfer of the hydride ion from **1** ^**H**^ and a proton from the acid (Scheme 2).

**Scheme 2:**
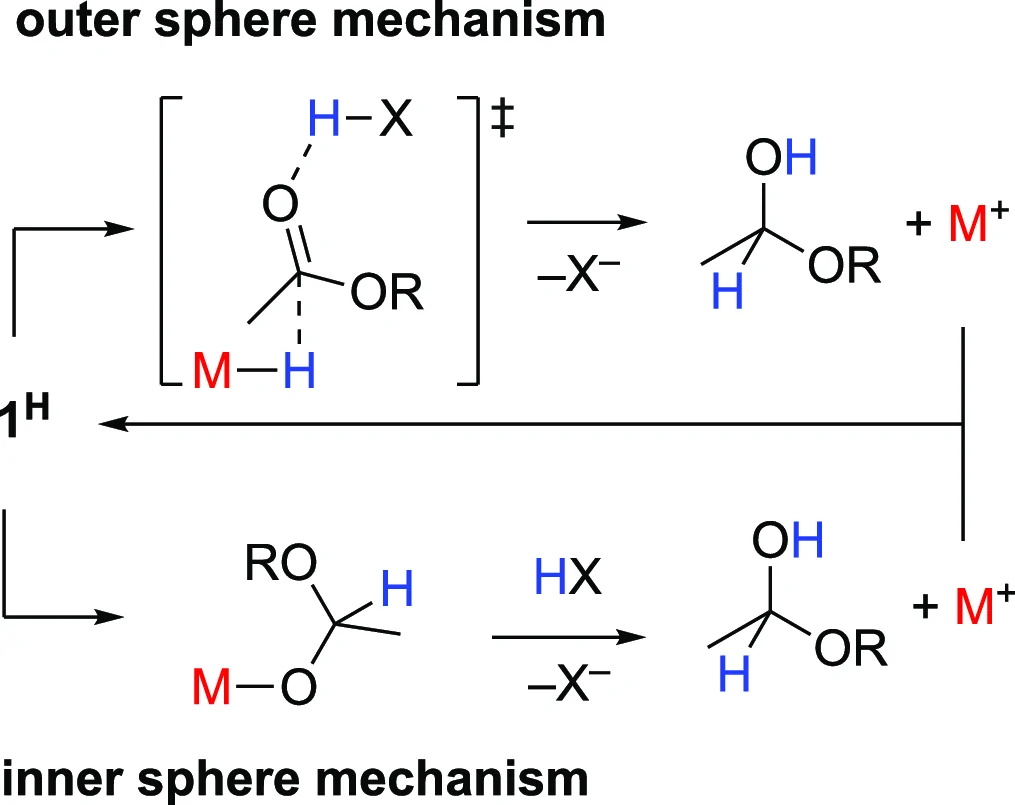
Simplified Scheme for the Two Mechanisms for the H^+^/H^–^ Transfer from **1** ^**H**^ and an Acid XH to the Ester; HX = Protonated Amine on the Ligand or TFE

In order to shed light on the mechanism of the hydride transfer step, quantum chemical calculations were performed at the B3LYP-D4/def2-TZVPP/SMD­(CH_3_CN)//B97–3c/CPCM­(CH_3_CN) level of DFT using ORCA 5.0.4. and 6.1.0 (see Supporting Information for details).^17^ Calculations were performed with TFE as the acid because the Faraday efficiency was slightly higher with TFE as the acid, pointing to less detrimental side reactions.

We assessed first whether **1** ^**H**^ or the protonated amine **1** ^**H**^ **H** ^**+**^, which could be formed in an equilibrium reaction with TFE, is the more plausible starting structure. Taking the p*K*_a_ values of Et_3_N and TFE/PhOH in MeCN as a reasonable estimate, the equilibrium is largely on the reactant side (p*K*_a_^TFE^ = 32.5;^18,19^ p*K*_a_^PhOH^ = 29.2;^20^ p*K*_a_^Et3N^ = 18.82 ^20^), even if homoassociation between the acid and its alkoxide is considered (Scheme 3).^21^

**Scheme 3:**
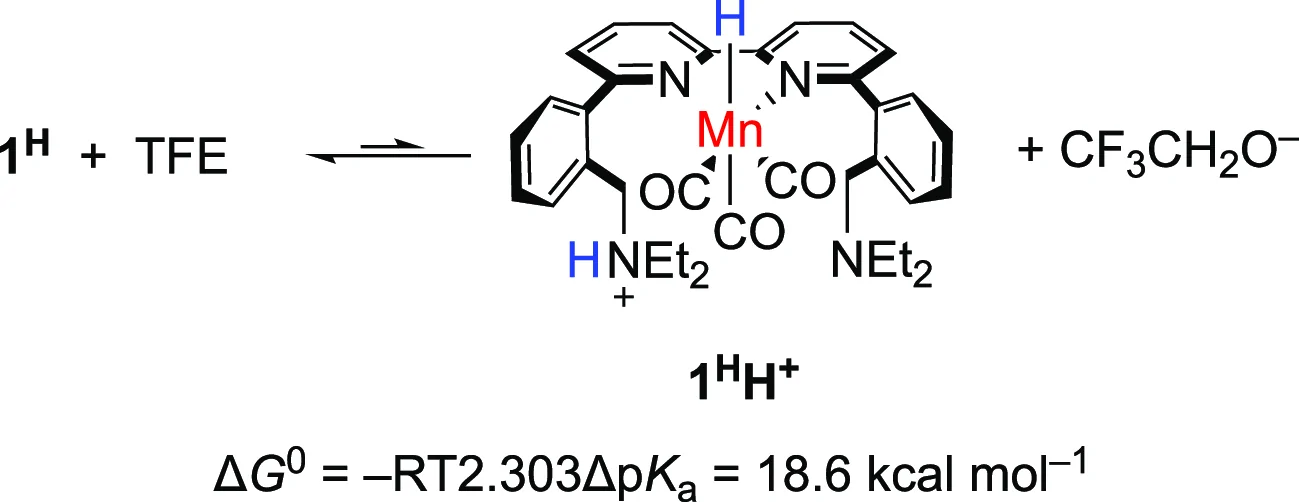
Protonation Equilibrium of **1^H^** with TFE

In line with previous calculations,^12^ the *cis* and *trans* conformers of the 6-fold coordinated species **1** ^**H**^, **1** ^**Br**^, **1** ^**MeCN+**^, and **1** ^**OCH2CF3**^ are very similar in energy with both conformers being feasible at room temperature (Table S1). For **1** ^**H**^, **1** ^**Br**^, and **1** ^**OCH2CF3**^, the *trans* conformers are energetically slightly favored, whereas for **1** ^**MeCN+**^, the *cis* conformer is slightly lower in energy. AIMD simulations by Nova and co-workers^12b^ on the anionic 5-coordinated precatalyst [Mn­(CO)_3_L]^−^ point to a fast rotation of the three carbonyl moieties at room temperature, enabling the formation of both hydride conformers upon protonation.^10b,12^ For the present system, we also assessed the *cis*/*trans* interconversion of the 6-fold coordinated hydride species **1** ^**H**^. The calculated pathway via rotation around the C^py^–C^ArNEt2^ bond gives an activation free energy of 20 kcal mol^–1^ relative to *trans*-**1** ^**H**^ (Figure S4), whereas the twist-type arrangement of the three CO ligands leads to a substantially larger barrier of about 31 kcal mol^–1^. We note, however, that the actual conformational exchange is likely to involve a more complex multidimensional motion than captured by this simplified scan/transition-state model. The results indicate that conformational interconversion has a sizable barrier and competes with substrate binding and hydride transfer. We thus investigated the ester-reduction pathways for both *cis*-**1** ^**H**^ and *trans*-**1** ^**H**^.

The reaction starts with an initial interaction of **2a** and **1** ^**H**^, which leads to the intermediate **Int1** via **TS2** (Figure 3, Figure S5). *cis*-**Int1** and *cis*-**TS2** are both energetically higher by 3 kcal mol^–1^ than the corresponding *trans* conformers likely due to steric repulsion between the NEt_2_ groups and the substrate. The computed activation barrier of 20 kcal mol^–1^ for the energetically preferred *trans* pathway is in a range expected for a slow reaction at room temperature. We also considered that the developing partial negative charge at the oxygen atom of **Int1** could be stabilized by hydrogen-bonding interactions to TFE. However, this does not lead to any significant change of the reaction profile for *trans*-**TS2** (*cf*. Figure S6).

**Figure 3:**
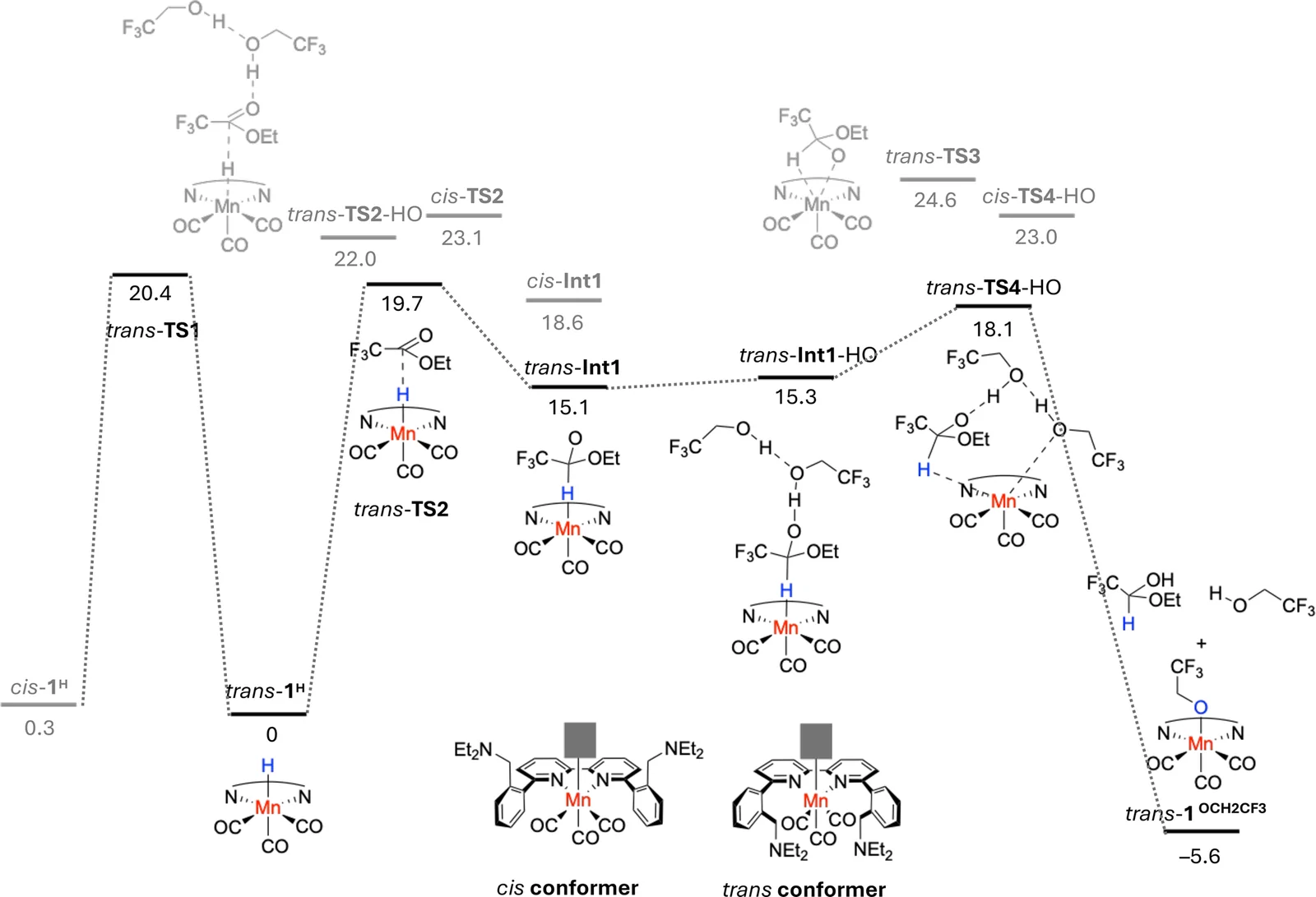
Computed lowest free energy pathway for the hydrogenation step of **2a** by **1^H^**; standard state in MeCN; free energies in kcal mol^–1^.

We then studied the two conceivable pathways for the hydride transfer step, i.e., an inner-sphere and an outer-sphere pathway (Figure S7, Figure S9). Along the inner-sphere pathway, the Mn­(I)-bound hemiacetal species *cis*-**1** ^**acetal**^ is formed via transition-state *cis*-**TS3** with a substantial activation barrier of 24 kcal mol^–1^ (Figure S7). Subsequent formation of the product is facile (Δ*G* ^0^ = −5 kcal mol^–1^) and initial protonation of *cis*-**1** ^**acetal**^ is facilitated by the pendant amine functionalities. The reaction sequence proceeds through various intermediates associated with an effective activation barrier of only 11 kcal mol^–1^ (Figure S8). In the outer-sphere concerted H^–^/H^+^ pathway, the hydride and the proton from TFE are directly transferred to the substrate. This pathway involves the formation of a hydrogen-bond network with two additional TFE molecules, which facilitates protonation of the hemiacetal and stabilizes the partial charge at one TFE in the transition state *cis-* **TS4**-HO. The computed activation barrier of 23 kcal mol^–1^ is very similar to the activation barrier in the inner-sphere pathway so that both pathways would contribute equally to the reactivity of the *cis* conformer.

In case of the *trans* conformer, the inner-sphere pathway proceeds via *trans*-**TS3**, which has a sizable barrier of 25 kcal mol^–1^ (Figure S9). In contrast, the outer-sphere transfer pathway *via trans*-**TS4-**HO has a substantially lower computed barrier energy of only 18 kcal mol^–1^ eventually leading to *trans*-**1** ^**OCH2CF3**^ as the initial product. This reaction pathway proceeds *via trans*-**Int1**-HO as the local minimum and benefits from hydrogen bonding of two TFE molecules providing the lowest energy route to formation of the product of this elementary step.

Taking the experimental and computational results together, we propose the following mechanism for the manganese-catalyzed hydrogenation of **2a** by protons and electrons: Initial 2e^–^ reduction of **1** ^**Br**^ in an EC^Br^E mechanism leads to the formation of [Mn­(CO)_3_L]^−^.^7e,10b^ Protonation of the highly nucleophilic Mn ion is facilitated by the amine functionalities with an estimated second-order reaction rate constant of 5 × 10^2^ M^–1^ s^–1^ for TFE as the acid yielding primarily *cis*-**1** ^**H**^.^7^ ^e^,^10^ ^b^ The calculated reaction profiles suggest interconversion between *cis*- and *trans*-**1** ^**H**^ and further that the reaction of *trans*-**1** ^**H**^ with **2a** is energetically favored through an outer-sphere pathway over both the inner-sphere pathway and the corresponding reaction of *cis*-**1** ^**H**^. This eventually leads to the formation of the hemiacetal and **1** ^**MeCN+**^, which is formed as the initial product according to IR-SEC studies. Finally, the hemiacetal releases the alcohol, and the resulting trifluoroethanal is reduced in a second catalytic cycle forming trifluoroethanol (Scheme 4). Hydrogenation of **2a** by **1** ^**H**^ has an upper limit of the catalytic fourth-order rate constant of <2 M^–3^ s^–1^ because we only observe modest current enhancement on the time scale of the CV (ν= 0.1 V s^–1^).

**Scheme 4:**
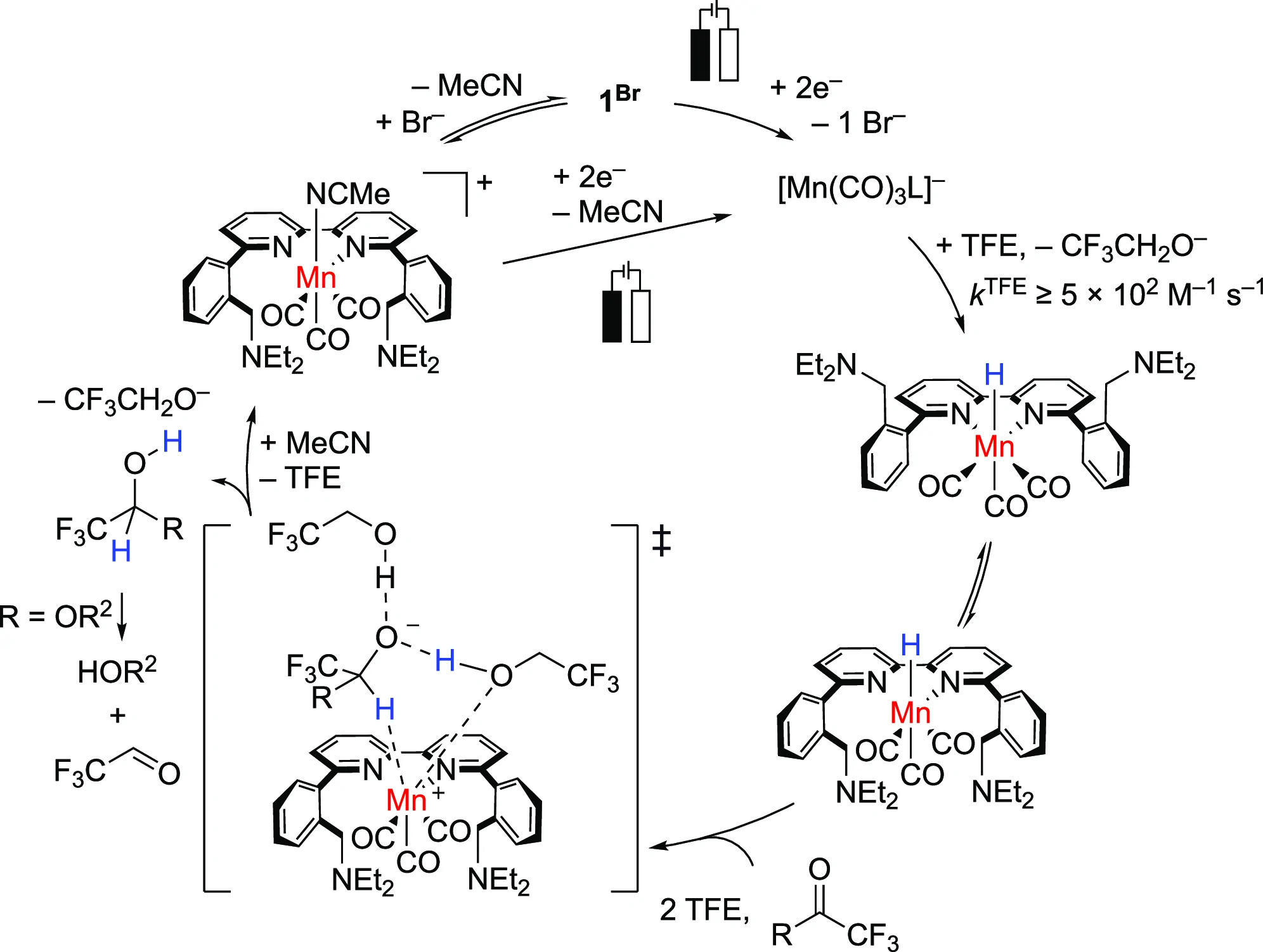
Proposed Reaction Mechanism for the Electrohydrogenation of Activated Esters by **1** ^**Br**^, R = H or OR^2^

## Conclusion

We report a molecular electrocatalyst for the reduction of activated esters consisting of a manganese bipyridine tricarbonyl complex with a proton relay. The catalyst enables the selective conversion of fluorinated esters to the corresponding alcohols under mild electrochemical conditions with a low catalyst loading. The studies demonstrate that some activation of the ester carbonyl atom is required to achieve efficient reduction, while the reaction shows high selectivity for C=O bonds over C=C functionalities. To our knowledge, this is the first base-metal system capable of the electrochemical hydrogenation of esters. Combined electrochemical, spectroscopic, and labeling experiments identify a manganese hydride species as the key reactive intermediate that is formed upon electrochemical reduction and subsequent protonation of the precatalyst. Computational studies provide insight into the hydrogenation step and identify the outer-sphere hydride/proton transfer pathway of the *trans* conformer as being the lowest in energy. These findings expand the scope of base-metal-catalyzed electrohydrogenation to activated esters and highlight the potential of manganese hydride complexes for selective electrocatalytic hydrogenation reactions using electrons and protons as hydrogen equivalents. More thermodynamically hydridic catalysts are likely required to activate less electrophilic carbonyl substrates.

## Experimental Section

All experimental details can be found in the SI.

## Supplementary Material





## Notes

Experimental details, CV data, IR-SEC data, NMR data, and computational details (PDF)
xyz coordinates of the calculated species (XYZ)

## References

[1] Fukuzumi S., Lee Y.-M., Nam W. (2018). Thermal and photocatalytic
production of hydrogen with earth-abundant metal complexes. Coord. Chem. Rev..

[2] Larionov E., Li H., Mazet C. (2014). Well-defined
transition
metal hydrides in catalytic isomerizations. Chem. Commun..

[3] Wang D., Astruc D. (2015). The golden age of transfer hydrogenation. Chem. Rev..

[4] Tilset M., Parker V. D. (1989). Solution homolytic bond dissociation
energies of organotransition-metal hydrides. J. Am. Chem. Soc..

[5] b Jacobsen, H. ; Berke, H. Chapter 4 - Hydridicity of Transition Metal Hydrides and its Implications for Reactivity, in Recent Advances in Hydride Chemistry; Elsevier; 2001.

[6] Matsunami A., Kayaki Y. (2018). Upgrading and expanding the scope
of homogeneous transfer hydrogenation. Tetrahedron
Lett..

[7] Shimakoshi H., Luo Z., Tomita K., Hisaeda Y. (2017). Cathodic reductive
couplings and hydrogenations of alkenes and alkynes catalyzed by the
B12 model complex. J. Organomet. Chem..

[8] Fokin I., Kuessner K.-T., Siewert I. (2022). Transition Metal Complex Catalyzed
Photo- and Electrochemical (De)­hydrogenations Involving C = O and
C = N Bonds. Synthesis.

[9] Gase phase bond dissociation energies. http://ibond.nankai.edu.cn/bde/.

[10] Fokin I., Denisiuk A., Würtele C., Siewert I. (2019). The Impact of a Proton Relay in Binuclear α-Diimine-Mn­(CO)_3_ Complexes on the CO_2_ Reduction Catalysis. Inorg. Chem..

[11] Rossenaar B.
D., Hartl F., Stufkens D. J., Amatore C., Maisonhaute E., Verpeaux J.-N. (1997). Electrochemical
and IR/UV–Vis Spectroelectrochemical Studies of fac-[Mn­(X)­(CO)_3_(iPr-DAB)]_n_ (n= 0, X = Br, Me, Bz; n= + 1, X =
THF, MeCN, nPrCN, P­(OMe)_3_; iPr-DAB = 1,4-Diisopropyl-1,4-diaza-1,3-butadiene)
at Variable Temperatures: Relation between Electrochemical and Photochemical
Generation of [Mn­(CO)_3_(α-diimine)]. Organometallics.

[12] Luthra M., Castro A. C., Balcells D., Daasbjerg K., Nova A. (2025). Metal-Dependent Mechanism of the
Electrocatalytic Reduction of CO_2_ by Bipyridine Complexes
Bearing Pendant Amines: A DFT Study. ACS Org.
Inorg. Au.

[13] Chalkley M., Garrido-Barros P., Peters J. C. (2020). A molecular mediator for reductive concerted proton-electron
transfers via electrocatalysis. Science.

[14] Modern Reduction Methods, Pher, G. ; Andersson; Munslow, I. J. , Eds.; Wiley, 2008.

[15] Electrolysis was also conducted at a higher overpotential of –1.9 V. This leads to a lower yield in the desired electrohydrogenation product **3a** and instead the yield of **5a** increased.

[16] Sampson M. D., Kubiak C. P. (2016). Manganese Electrocatalysts with Bulky Bipyridine Ligands:
Utilizing Lewis Acids To Promote Carbon Dioxide Reduction at Low Overpotentials. J. Am. Chem. Soc..

[17] Neese F. (2025). Software Update: The ORCA Program
System–Version 6.0. WIREs Comput. Mol.
Sci..

[18] Kütt A., Selberg S., Kaljurand I., Tshepelevitsh S., Heering A., Darnell A., Kaupmees K., Piirsalu M., Leito I. (2018). pKa values in organic chemistry –
Making maximum use of the available data. Tetrahedron
Lett..

[19] Lam Y. C., Nielsen R. J., Gray H. B., Goddard W. A. (2015). A Mn Bipyrimidine
Catalyst Predicted To Reduce CO_2_ at Lower Overpotential. ACS Catal..

[20] Kaljurand I., Kutt A., Soovali L., Rodima T., Maemets V., Leito I., Koppel I. A. (2005). Extension of the self-consistent
spectrophotometric basicity scale in acetonitrile to a full span of
28 p*K*
_a_ units: unification of different
basicity scales. J. Org. Chem..

[21] Fourmond V., Jacques P. A., Fontecave M., Artero V. (2010). H_2_ evolution and molecular electrocatalysts:
determination of overpotentials and effect of homoconjugation. Inorg. Chem..

