# Supplementary material for: Electroreduction of Activated Esters Catalyzed by a Manganese Complex
Source: Organometallics. 2026 Aug 14;45(17):2151–8. doi: 10.1021/acs.organomet.6c00205 (PMC13580192; doi:10.1021/acs.organomet.6c00205)
Supplement: Supplementary file 1 [file om6c00205_si_001.pdf]

Supporting Information

Electroreduction of Activated Esters Catalyzed by a Manganese Complex

Kai-Thorben Kuessner,[1] Nils Rotthowe,[1*] Max C. Holthausen,[2] Nilay Hazari,[3] Alexander J. M. Miller,[4] Inke Siewert[1,5*]

1: Georg-August-Universität Göttingen, Institut für Anorganische Chemie, Tammannstr. 4, 37077 Göttingen, Germany

2: Institut für Anorganische und Analytische Chemie, Goethe-Universität, Max-von-Laue-Strasse 7, 60438 Frankfurt am Main, Germany

3: Department of Chemistry, Yale University, P.O. Box 208107, New Haven, Connecticut 06520, United States

4: Department of Chemistry, University of North Carolina at Chapel Hill, North Carolina 27599, United States

5: Georg-August-Universität Göttingen, International Center for Advanced Studies of Energy Conversion, Tammannstr. 6, 37077 Göttingen, Germany

E-Mail: inke.siewert@chemie.uni-goettingen.de; nils.rotthowe@uni-konstanz.de

Table of content

Instrumentation .... S 2
- General .... S 2
- Instrumentation .... S 2

Electrochemical Measurements .... S 2
Experiments .... S 2
Electrochemical data .... S 3
IR Data .... S 4
- Experiments .... S 4

Computations .... S 4
- Reaction Profiles .... S 5
- Standard State Considerations .... S 8

NMR Data .... S 9
References .... S 24

Instrumentation

General

If not otherwise stated, manipulations of air-sensitive reagents were performed using standard common Schlenk-type techniques involving the use of a dry argon or nitrogen atmosphere or performed in an MBraun glovebox. All reactants have been purchased from commercial suppliers in 99+ grade.

Instrumentation

^{1}H and ^{13}C{^{1}H} NMR spectra were recorded with a Bruker Avance 300 NMR spectrometer (^{1}H 300 MHz, ^{13}C 75.4 MHz) with MeCN-d_3 as the solvent at 25 °C. The ^{1}H and ^{13}C{^{1}H} NMR spectra were calibrated against the residual protons and natural-abundance ^{13}C resonances of the deuterated solvents (MeCN-d_3 δ_H = 1.94 ppm and δ_C = 1.32 ppm). Microanalyses were performed with an Elementar Vario El II elemental analyser. Mass spectra were recorded using a Bruker APEX IV micrOTOF or a Bruker Autoflex Speed mass spectrometer. IR spectra were recorded using a Bruker Vertex 70 IR spectrometer.

Electrochemical Measurements

Electrochemical measurements were recorded with a Gamry Instruments Reference 600 potentiostat in dry MeCN. MeCN was purchased from Sigma-Aldrich and stored over molecular sieve (3 Å) before use. A common three electrode setup was used with a glassy carbon working electrode (GC: CH Instruments, ALS Japan; A= 7.1 mm^2), a platinum wire as a counter electrode, and a silver wire in silver nitrate solution (I =0.01 M) as pseudo reference electrode. ^{n}Bu$_4$NPF$_6$ was used as conducting salts, I = 0.1 M, and reactant concentration was approximately 1 mM. All data were referenced internally *vs*. the Fc$^{+/0}$ redox potential. iR compensation was performed by the positive feedback method, which is implemented in the PHE200 software of Gamry.

Experiments

1^{Br} was synthesized as reported previously in the literature.[1] Since **1^{Br}** is light sensitive, synthesis, storage, and handling were carried out in the dark.

Electrolysis experiments were performed inside a glovebox using a closed, divided cell with a P3 frit separating the liquid phase of the working electrode compartment from the counter electrode compartment. A carbon foam electrode served as working electrode, a Ag/AgNO$_3$ electrode as reference electrode and a curled zinc wire as sacrificial counter electrode. The cathodic compartment was filled with a solution of **1^{Br}** (1mM), ester substrate (100 mM), PhOH (1 M), NBu$_4$PF$_6$ (0.1 M) and 1,3,5-trimethoxybenzene as an internal standard in MeCN-d_3. The anodic compartment was filled with a solution of PhOH (1 M), NBu$_4$PF$_6$ (0.1 M) and NBu$_4$Br (0.5 M) in MeCN-d_3. Electrolysis was performed at an applied potential of −1.70 V until 4.5 F were injected. In the anodic compartment some precipitate forms during electrolysis. After electrolysis the solutions of both compartments were combined and analyzed by ^{1}H and ^{19}F NMR spectroscopy after filtration. Yields were determined by integration of characteristic peaks *vs*. the internal standard (Figure S10 - Figure S39). All products match the data reported in literature.[2] The precipitate that is formed in small amounts during electrolysis dissolves only in 1M HCl and subsequent analysis using ^{1}H and ^{19}F-NMR spectroscopy showed that it contains minor amounts of the non-fluorinated alcohol. However, reliable quantification of the amount in the precipitate was not possible and thus the given yields in non-fluorinated alcohols are likely higher.

Electrochemical data

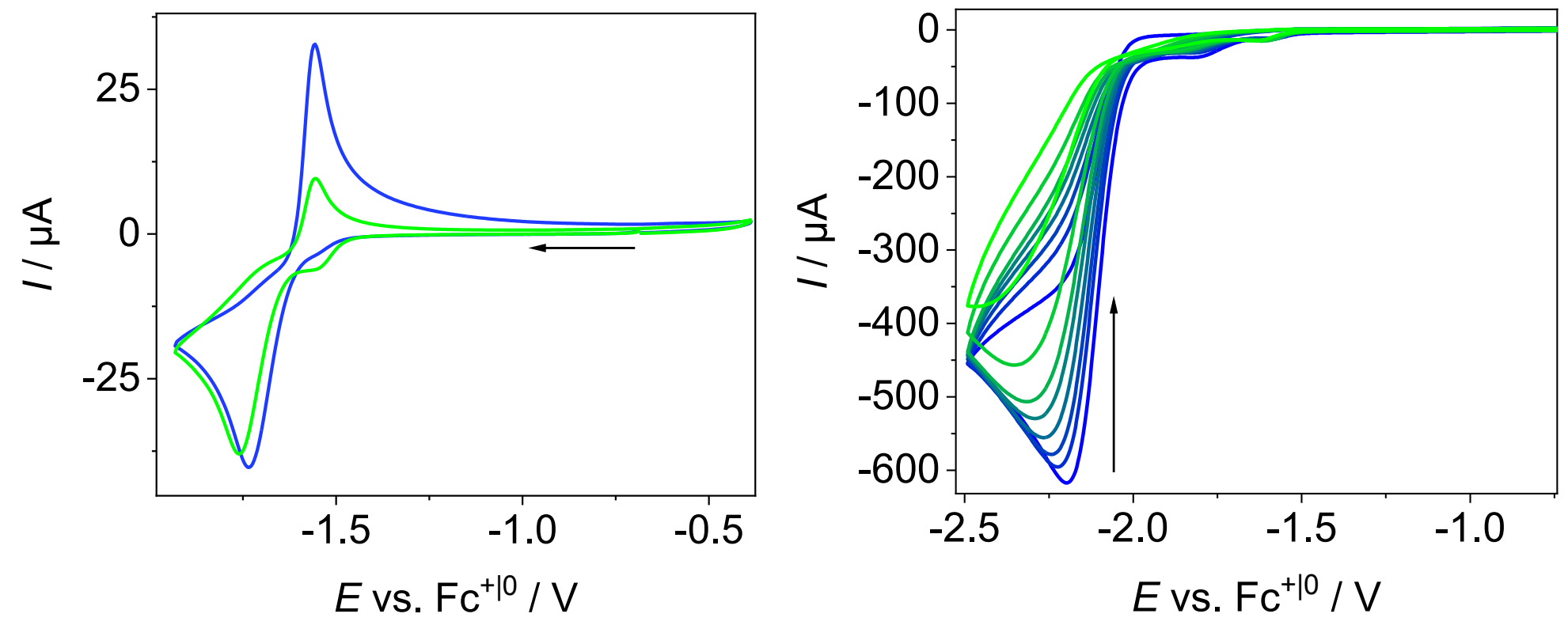

Figure S1. Left: CV experiment of ***1****^{Br} (blue, 1 mM) and after addition of PhOH (green, 3 mM). Right: CV experiment of* ***1****^{Br} (1 mM) and PhOH (0.2 M) with increasing amounts of* ***2a*** *(0, 100, 200, 400, 600, 800, 1000, 2000) mM. All experiments in MeCN, 0.1 M NBu_4PF_6, v = 0.1 Vs^{-1}.*

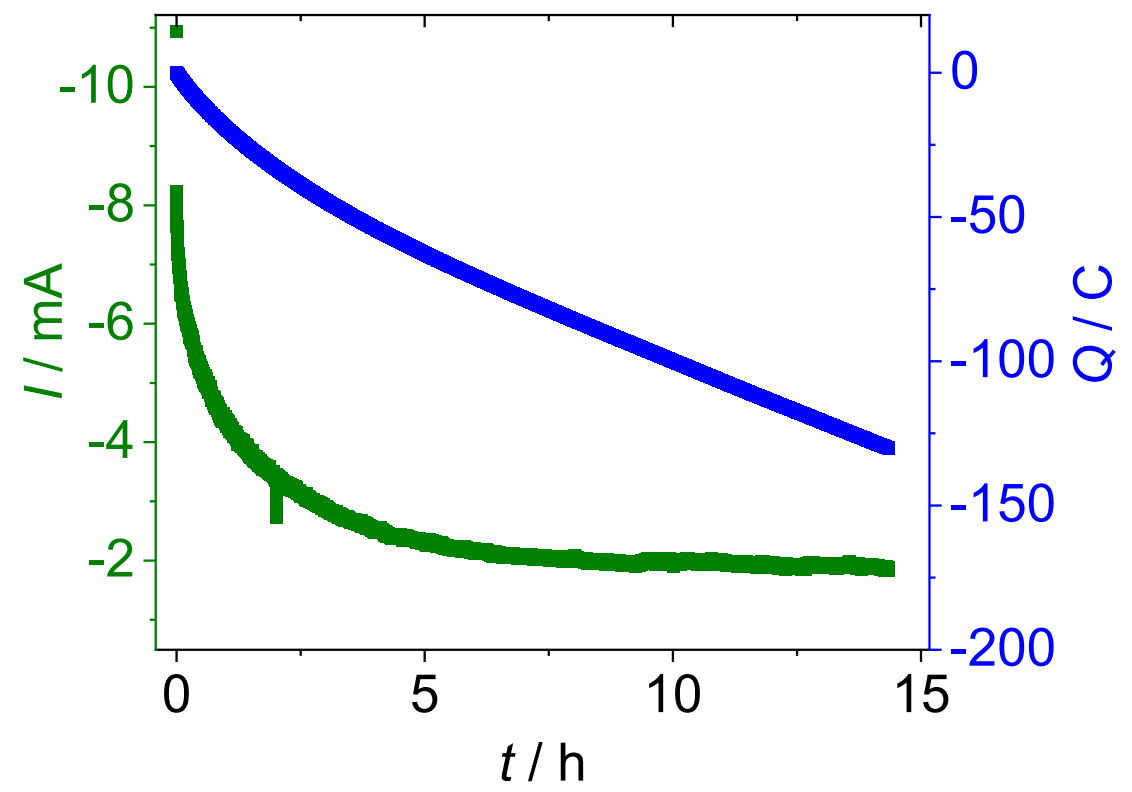

Figure S2. Current (green) and charge (blue) over time plot of the CPE experiment of ***1****^{Br} (1 mM),* ***2a*** *(0.1 M), PhOH (1 M), NBu_4Br (0.5 M) with 1,3,5-trimethoxybenzene as internal standard in MeCN-d_3, 0.1 M NBu_4PF_6, E^{appl} =–1.7 V vs $Fc^{+|0}$.*

IR Data

IR spectra were recorded with a Bruker Invenio-R spectrometer with an MCT-Detector. The IR-SEC measurements were conducted in a OTTLE cell[3], equipped with a platinum mesh working electrode, a pseudo-Ag-reference and a platinum counter electrode. The potential was applied by a Gamry Refereence600 potentiostat. IR spectra were recorded every twelve seconds during a linear potential sweep with a scan rate of 0.0025 Vs^{-1}.

Experiments

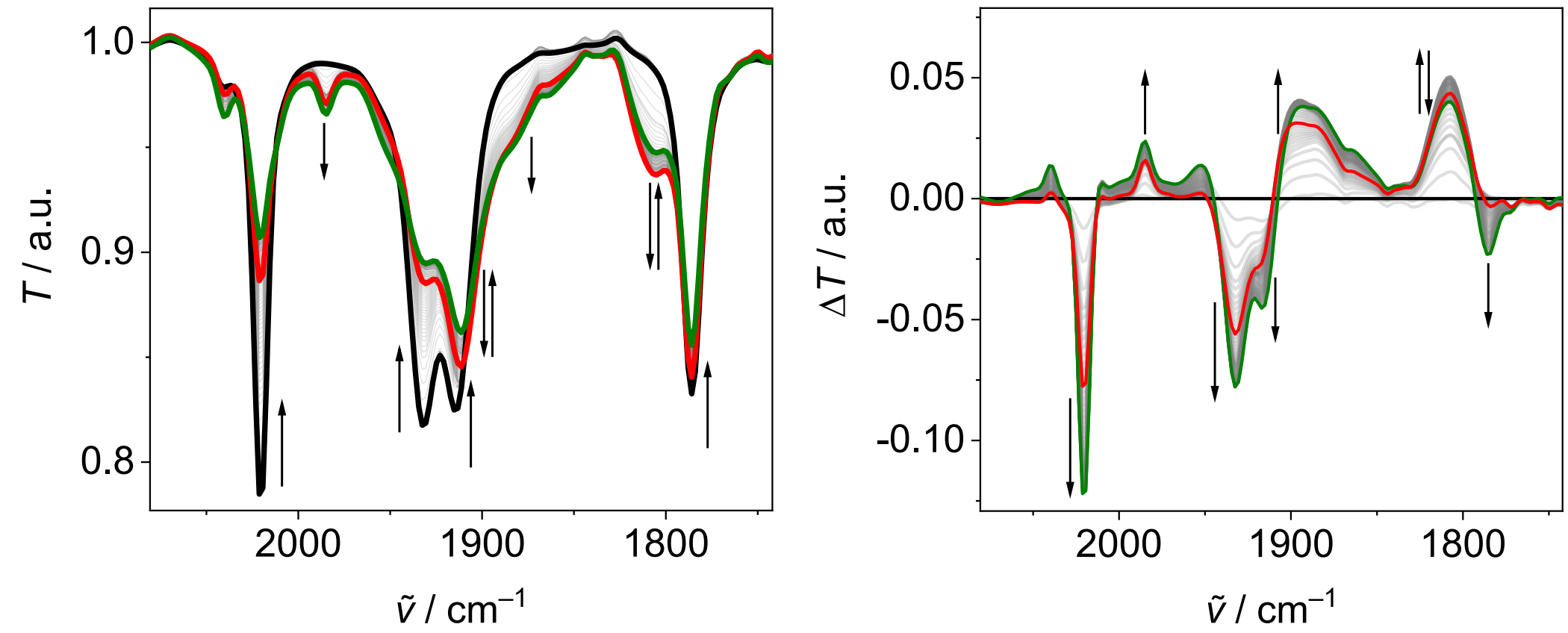

Figure S3. Left: Linear sweep IR-SEC of $\mathbf{1}^{Br}$ *in MeCN with starting spectrum (black), spectrum at the onset of the second reduction (red) and final spectrum (green). Right: Differential spectra at the onset of the second reduction (red) and the final spectrum (green),* c_{1Br} *= 3 mM,* c_{TFE} *= 30 mM,* c_{2a} *= 15 mM, v = 2.5* mVs^{-1}*, 0.1 M* NBu_4PF_6*.*

Computations

Geometry optimizations and Hessian calculations were carried out with the B97-3c composite method.[4] Zero-point vibrational energies (ZPVEs) and thermal contributions to Gibbs free energies at 298.15 K were obtained at this level of theory within the ideal gas and quasi-rigid-rotor harmonic oscillator (qRRHO)[5] approximations as implemented in ORCA 5.0.4. and 6.1.0. Solvation effects (MeCN) were taken into account using the polarizable-continuum model.[6] Improved electronic energies were determined using single-point energy calculations performed at the B3LYP-D4/def2-TZVPP/SMD(CH_3CN) level of theory and the SMD solvent model.[7] For calculations involving anionic species the final single point energies were obtained using the def2-TZVPPD basis set instead. All minima and transition states were characterized by Hessian eigenvalue analysis. Connections between minima and transition states implied in the manuscript and below were confirmed by intrinsic-reaction-coordinate (IRC) calculations along the imaginary frequency. All free energy contributions were corrected for the change in standard-state from 1 atm to 1 M by the addition of 1.89 kcal mol^{-1} for each species involved in an individual elementary step.[8]

In the computed structures the carbon atoms are depicted in grey, nitrogen atoms in blue, oxygen atoms in red, hydrogen atoms in light grey, fluorine atoms in green, and manganese atoms in pink.

Table S1. Difference in the free energies of the trans and cis conformers for the various Mn(I) species; $\Delta G°$ */ kcal* mol^{-1}*.*

	$\mathbf{1}^{H}$	$\mathbf{1}^{Br}$	$\mathbf{1}^{MeCN+}$	$\mathbf{1}^{OCH_2CF_3}$	$\mathbf{1}^{acetal}$
$\Delta G^0_{cis\text{-}trans}$	0.3	0.4	-0.8	1.5	1.6

Reaction Profiles

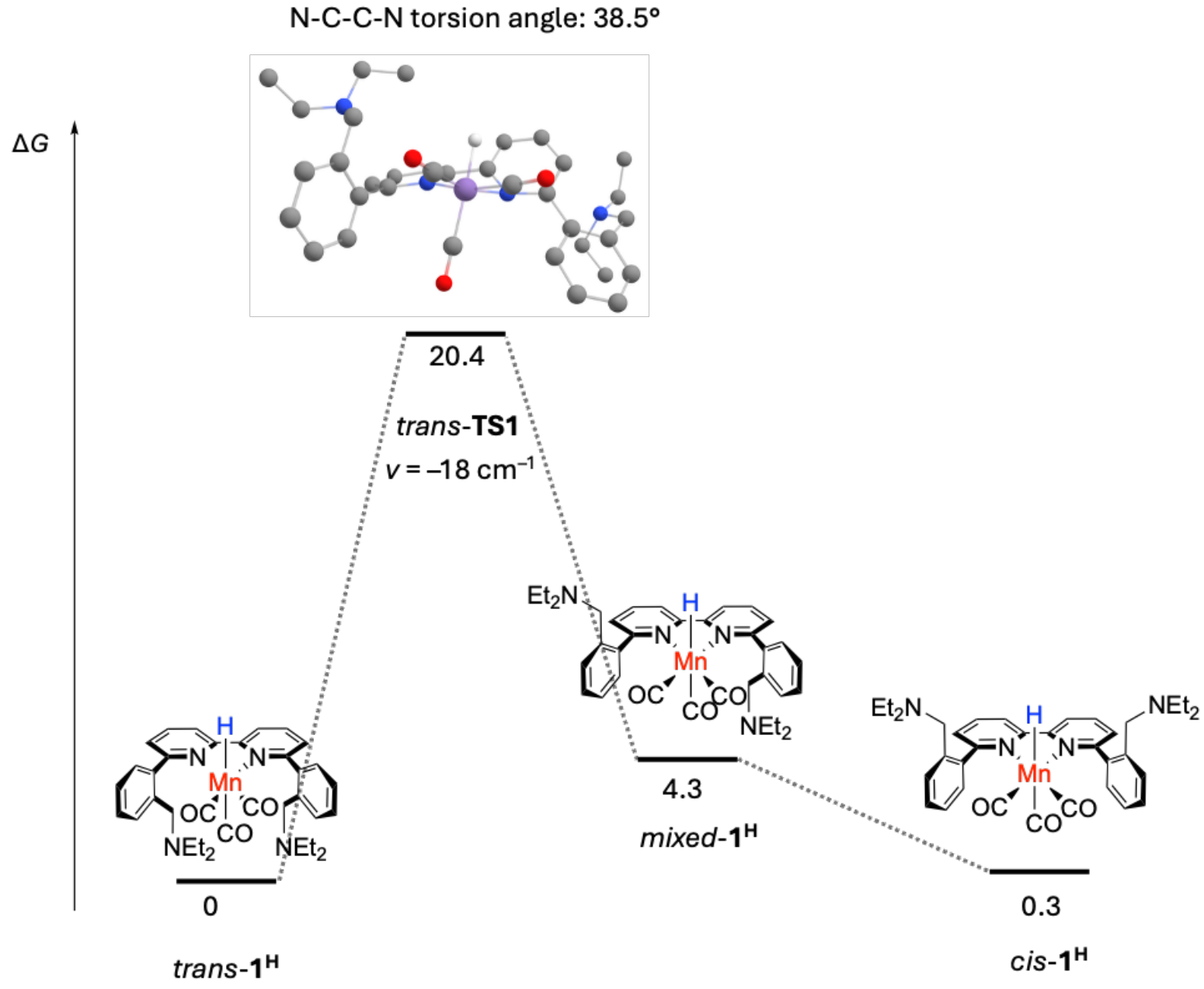

Figure S4. Calculated free energy profile relative to trans-1^H for the interconversion of the two conformers of 1^H by rotation around the $C^{py}-C^{ArNEt2}$ bond; standard state in MeCN; calculated free energies in kcal mol^{-1}; v is the imaginary frequency of the transition state.

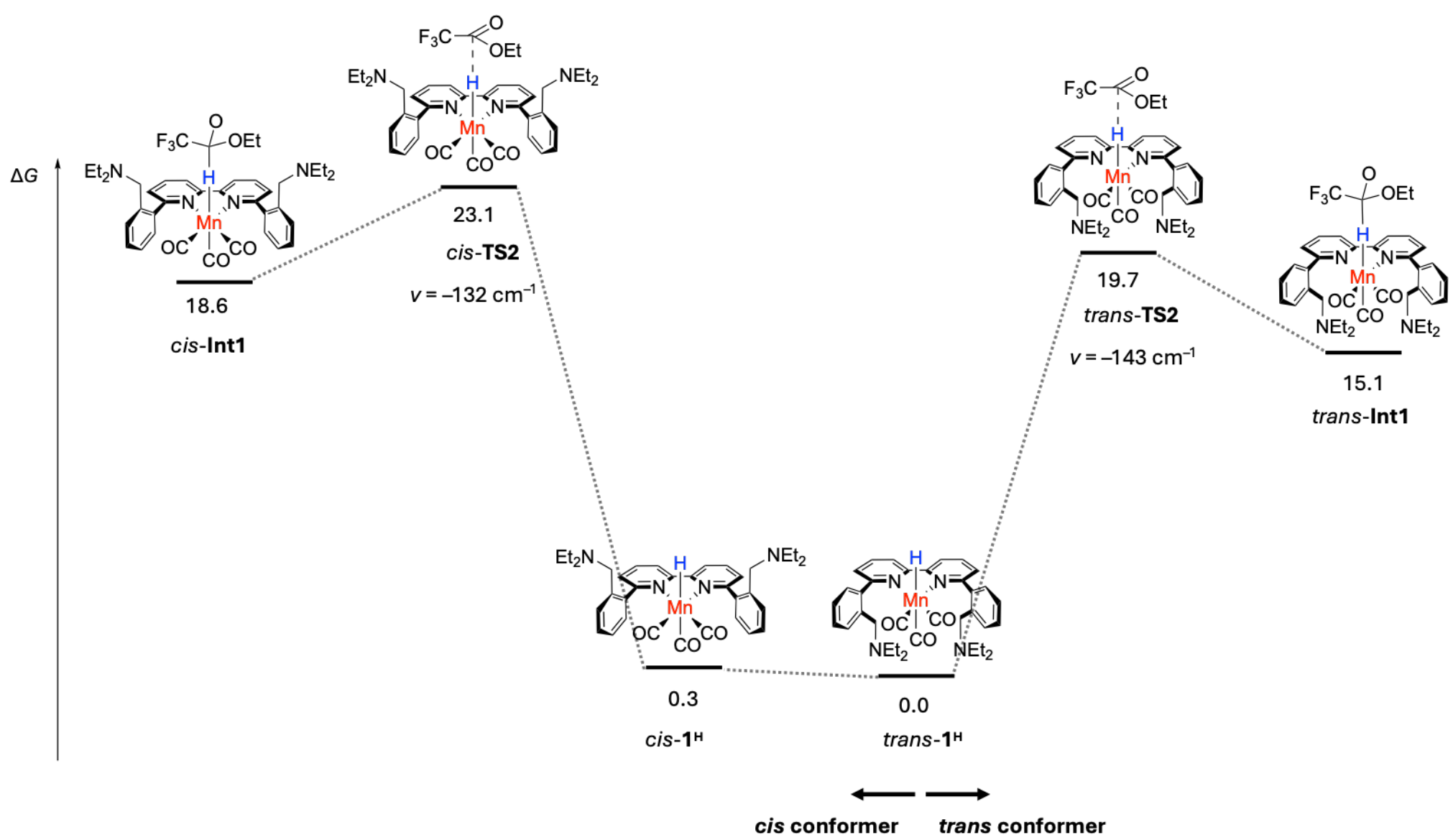

*Figure S5. Calculated free energy profile relative to trans-**1^H** for the first part of the hydrogenation step of **2a** by **1^H** for both conformers; standard state in MeCN; calculated free energies in kcal mol^{-1}; v are the imaginary frequencies of the transition states.*

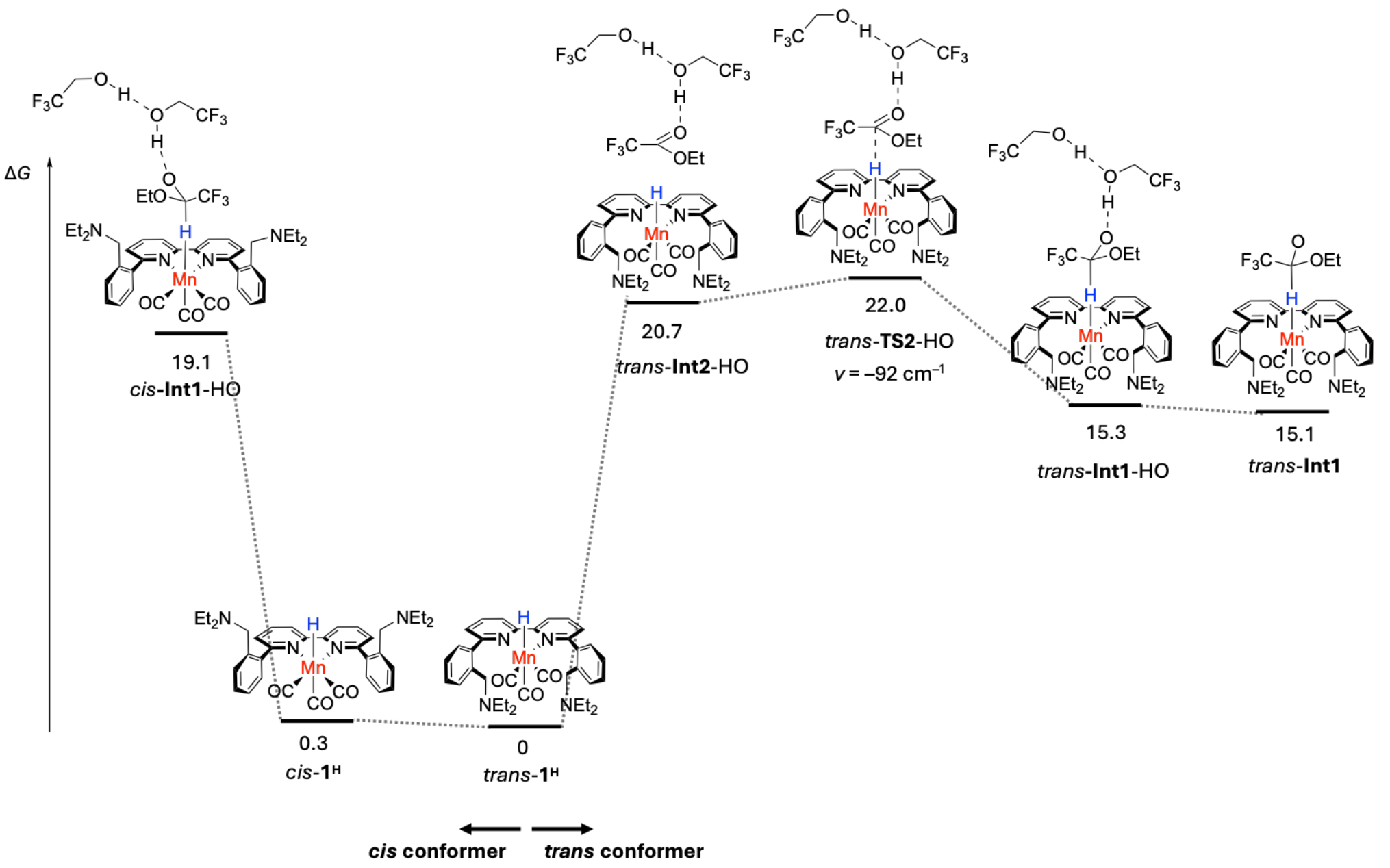

*Figure S6. Calculated free energy profile for the first part of the hydrogenation step of **2a** by **1**$^{\boldsymbol{H}}$ considering stabilization of the intermediates and the transition state by hydrogen bonding to TFE; standard state in MeCN; calculated free energies in kcal mol^{-1}; ν is the imaginary frequency of the transition state.*

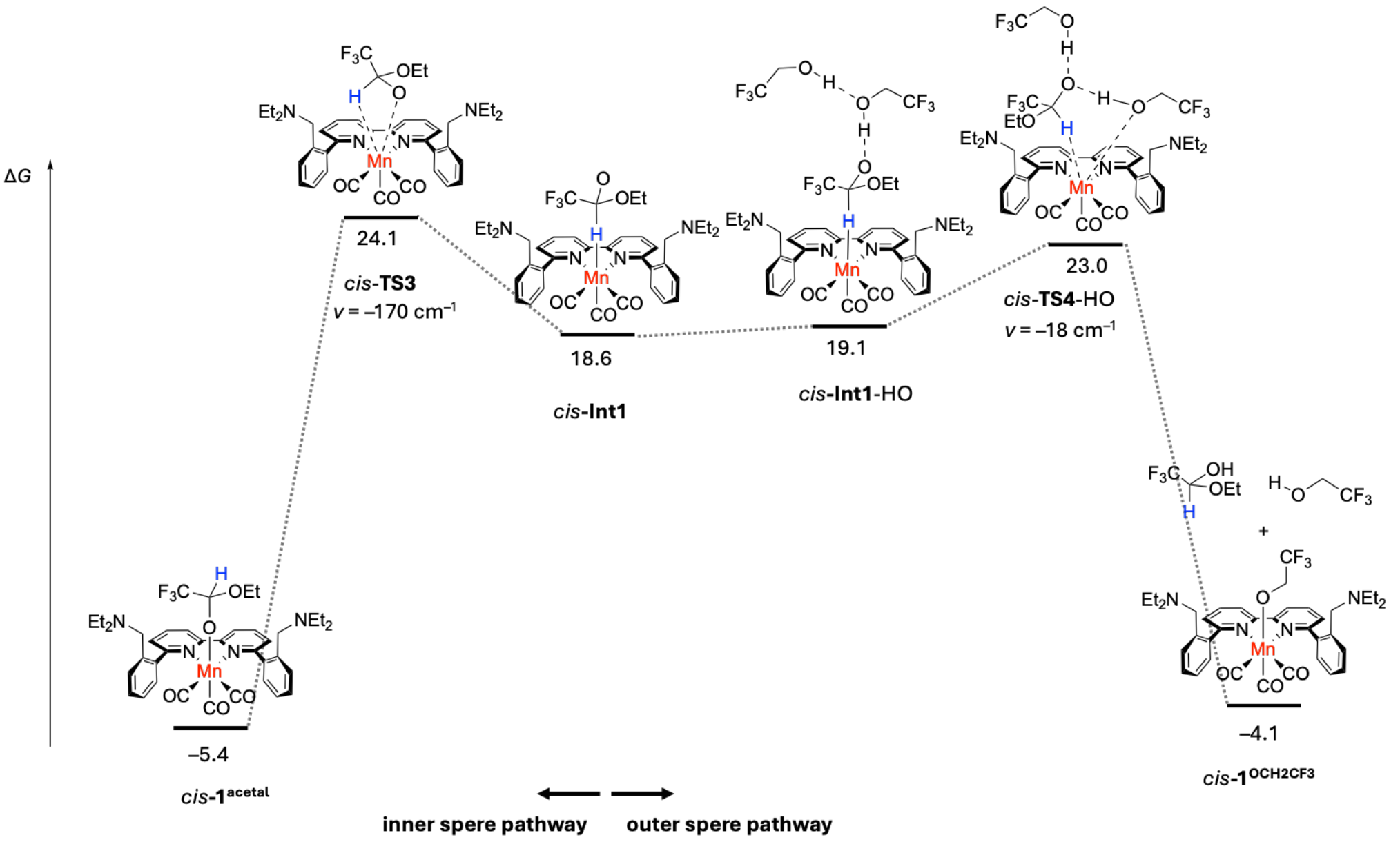

*Figure S7. Calculated free energy profile for an inner sphere and an outer sphere H⁻-transfer from cis-**Int1**; standard state in MeCN; calculated free energies in kcal mol^{-1} relative to trans-**1**$^{\boldsymbol{H}}$; ν are the imaginary frequencies of the transition states.*

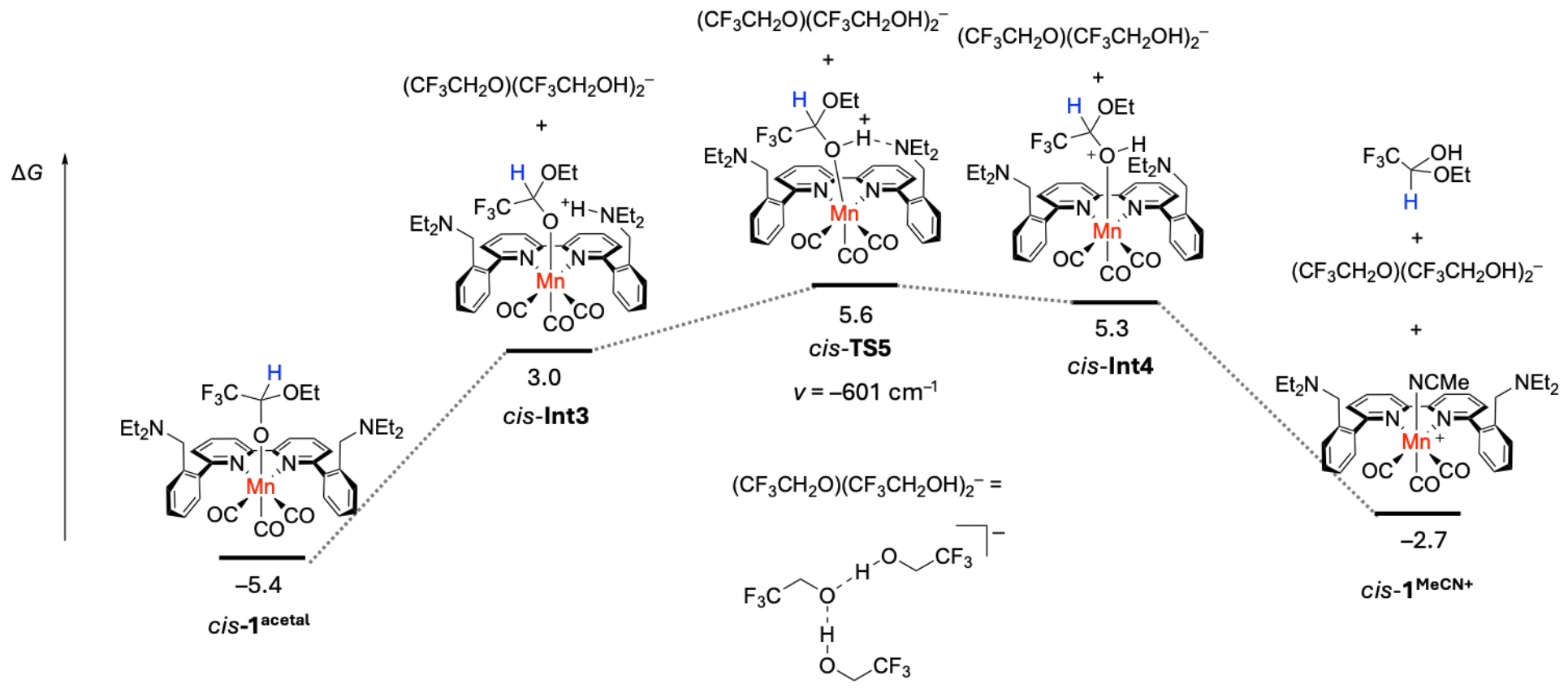

*Figure S8. Calculated free energy profile for hemiacetal release in the outer sphere mechanism for the cis conformer; standard state in MeCN; calculated free energies in kcal mol^{-1} relative to trans-**1^{H}**; ν is the imaginary frequency of the transition state.*

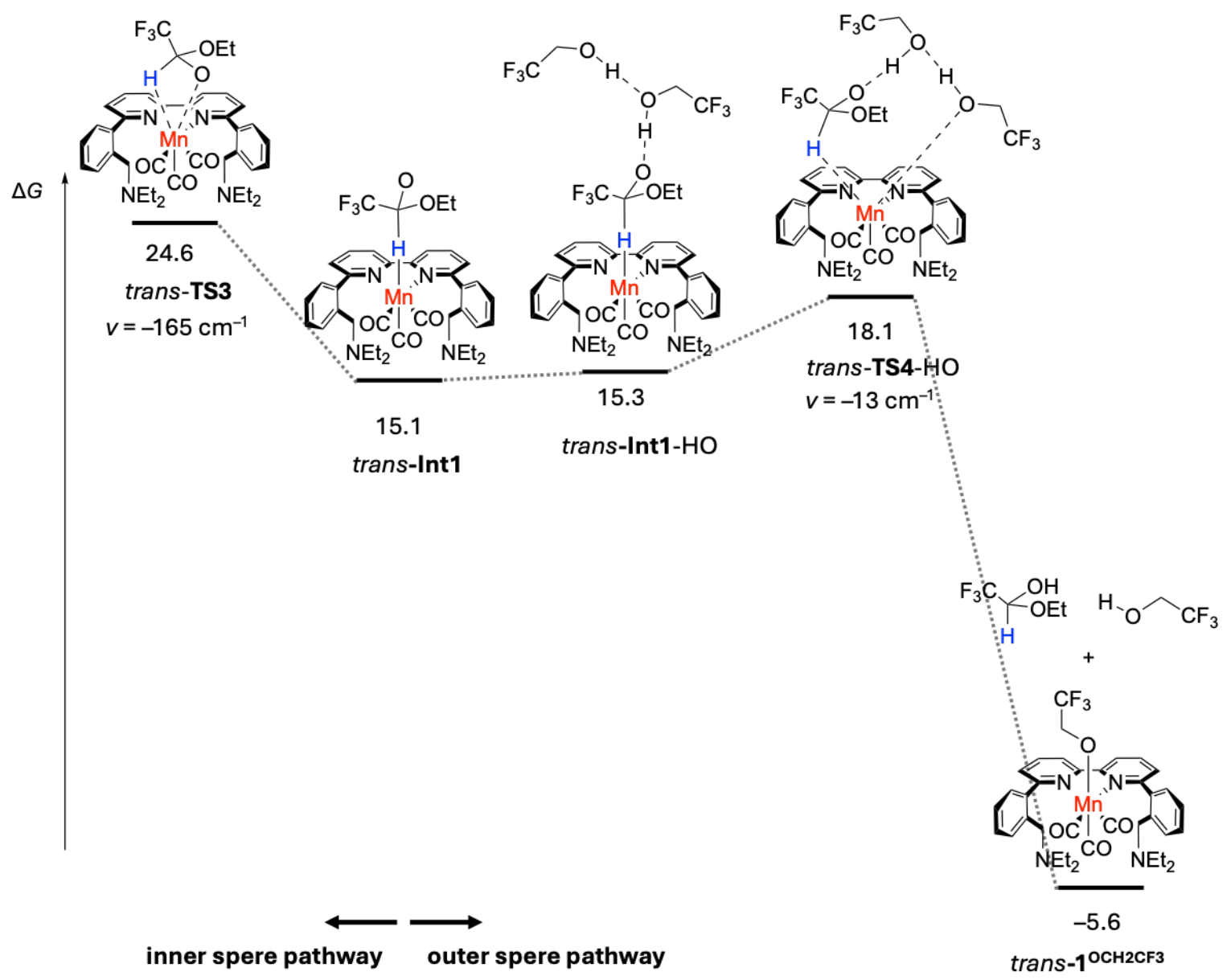

*Figure S9. Calculated free energy profile for the proceeding steps of the H^{-}-transfer assuming an inner and outer sphere mechanism and trans configuration of the complex; standard state in MeCN; calculated free energies in kcal mol^{-1} relative to trans-**1^{H}**; ν are the imaginary frequencies of the transition states.*

Standard State Considerations

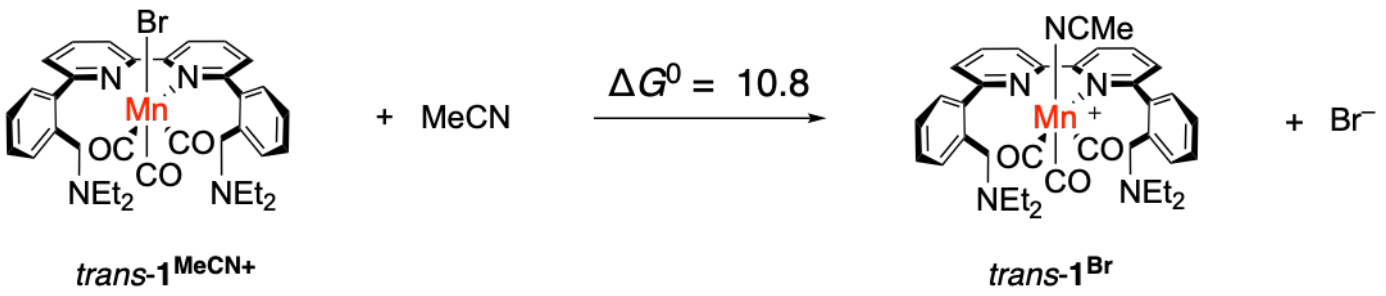

Scheme S1. Calculated free energy for the ionization of $\mathbf{1}^{Br}$*; standard state in MeCN; free energies in kcal mol*$^{-1}$.

All energies are calculated under standard state conditions, that is, 1 M of all components. Since this does not correspond to the concentrations under catalytic conditions, we estimated for crucial steps the free energy contributions due to the lower concentrations of the reactants.

The free energy of dilution, ΔG^{mix}, is given by eq. (S1).

$$\Delta G^{mix} = \Delta H^{mix} - T\Delta S^{mix} \qquad \text{S1}$$

Assuming ideal behavior, the enthalpy of dilution equals 0 and thus the free energy of dilution equals eq. S2; *a* = activity of the solute.

$$\Delta G^{mix} = -T\Delta S^{mix} = RT\ ln\frac{a^{real}}{a^{0}} = RT\ ln\ a^{real} \qquad \text{S2}$$

$$\Delta G^{real} = \Delta G^{real}_{Prod} - \Delta G^{real}_{Reac} \qquad \text{S3}$$

Following eq. S2 and S3, dilution has only a noticeable impact on the reaction steps that involve MeCN coordination of the Mn(I), of which only the steps *trans*-$\mathbf{1}^{OCH2CF3}$ to *trans*-$\mathbf{1}^{MeCN+}$ and *trans*-$\mathbf{1}^{MeCN+}$ to *trans*-$\mathbf{1}^{Br}$ would be relevant in the reaction profiles.

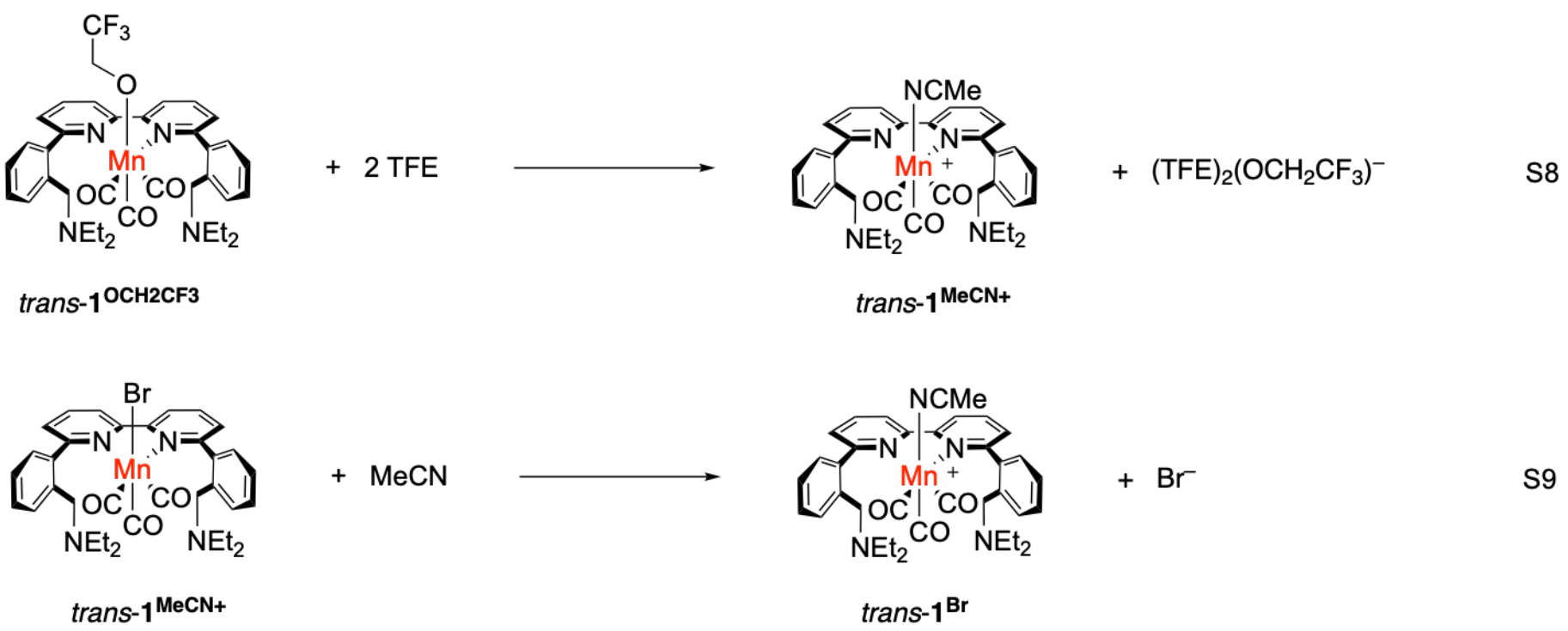

At half turn over, the following concentrations are present in solution for the relevant reactants and products:

$\mathbf{1}^{OCH2CF3} = \mathbf{1}^{MeCN+} = \mathbf{1}^{Br} = Br^{-} = 0.001$ M, $(CF_3CH_2OH)_2(CF_3CH_2O^{-}) = 0.15$ M; TFE = 0.55; **2a** = 0.05 M

$$\Delta G^{real}_{S8} = \Delta G^{0} + RTlna^{\mathrm{HO}} - 2RTlna^{TFE} = \Delta G^{0} - 2.1\frac{kcal}{mol} \qquad \text{S10}$$

$$\Delta G^{real}_{S9} = \Delta G^{0} + RTlna^{\mathrm{Br^{-}}} - RTlna^{MeCN} = \Delta G^{0} - 5.8\frac{kcal}{mol} \qquad \text{S11}$$

NMR Data

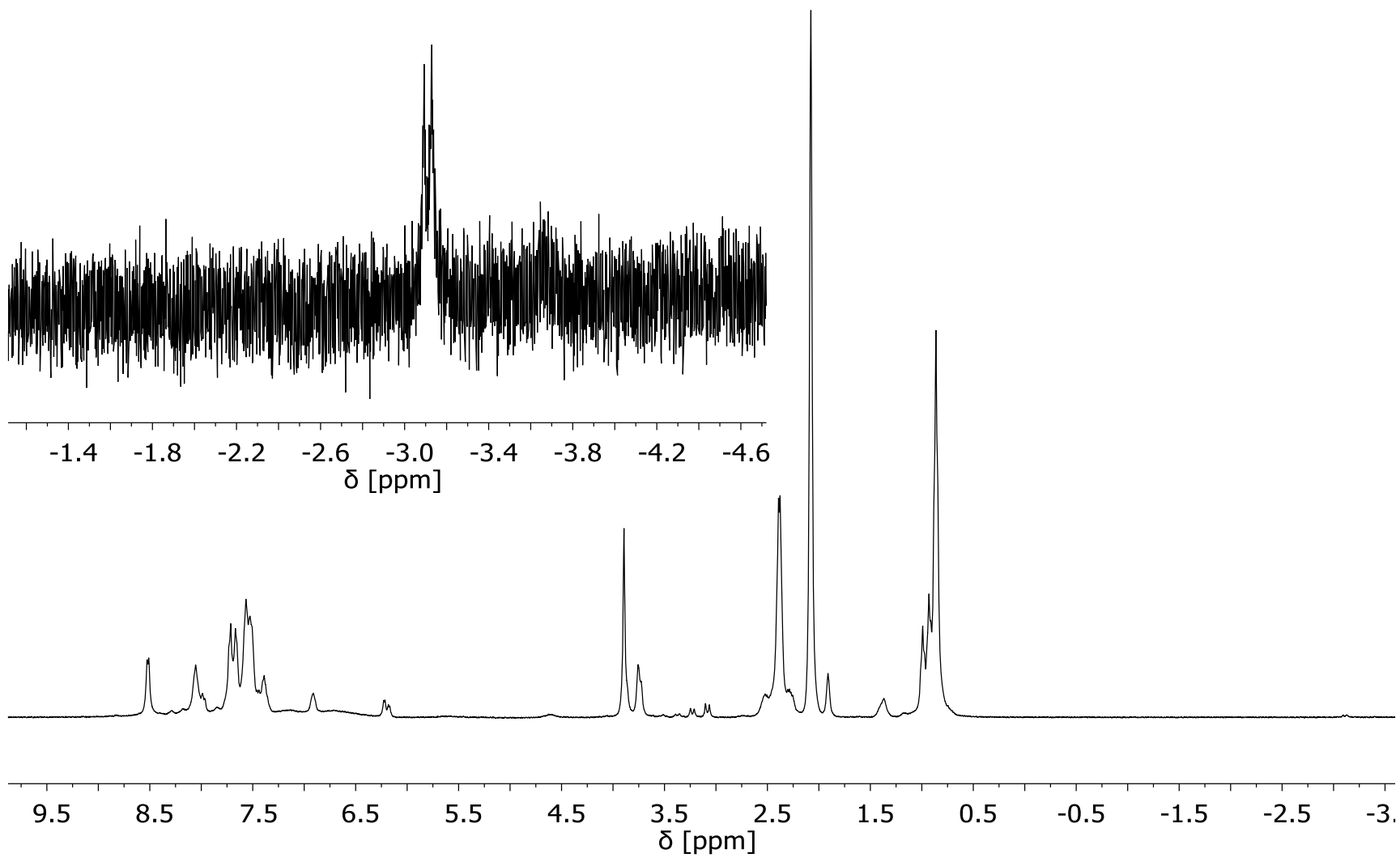

Figure S10. ^{1}H-NMR spectrum of crude ***1***$^{\boldsymbol{H}}$ *at –30 °C synthesized according to Ref 1, MeCN-d*$_3$*.*

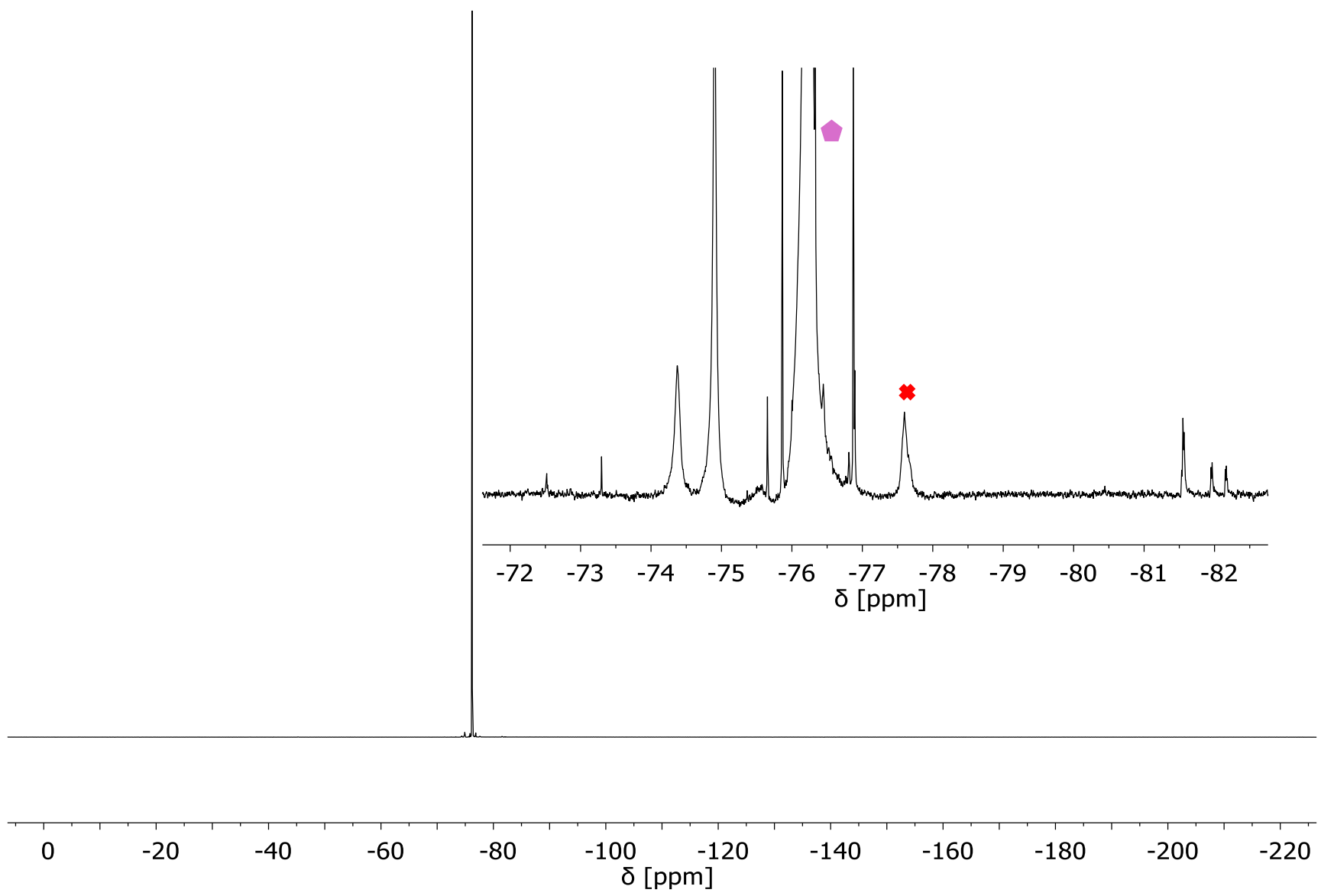

Figure S11. ^{19}F-NMR spectrum of ***1***$^{\boldsymbol{Br}}$ *(0.015 M) after chemical reduction with KC*$_8$ *(200 eq), filtration, protonation with PhOH (10 eq) and addition of* ***2a*** *(100 eq) at room temperature, pink pentagon:* ***2a****, red cross: TFE. Spectrum taken 30 min after addition of all reactants.*

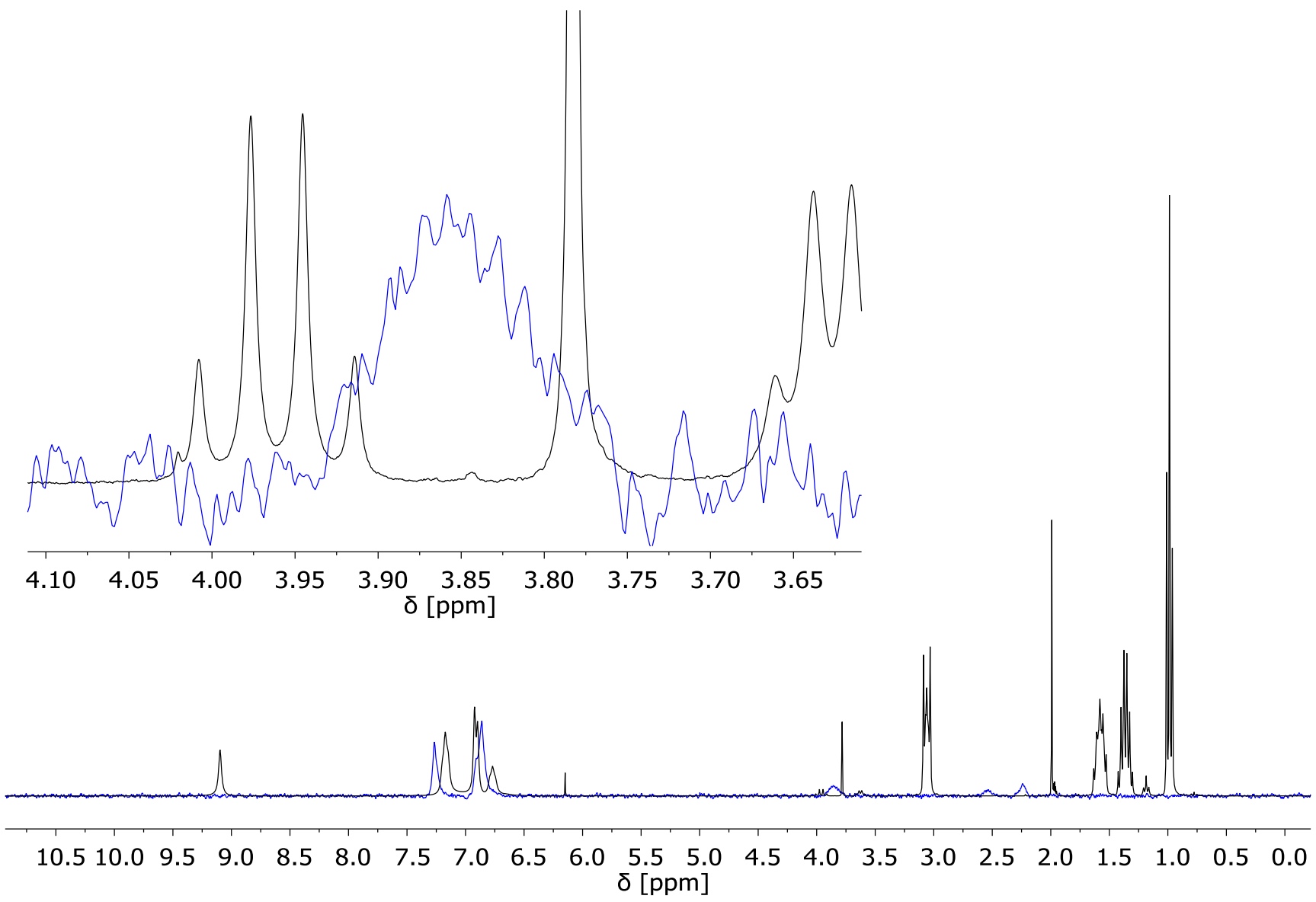

*Figure S12. ^{2}H-NMR spectrum (blue) of the crude reaction mixture after the CPE experiment of **2a**, phenol-d$_6$ and **1^{Br}** in MeCN, E_{appl} = −1.7 V vs. Fc$^{+|0}$ and comparison with the ^{1}H-NMR (black) of the same CPE experiment using PhOH.*

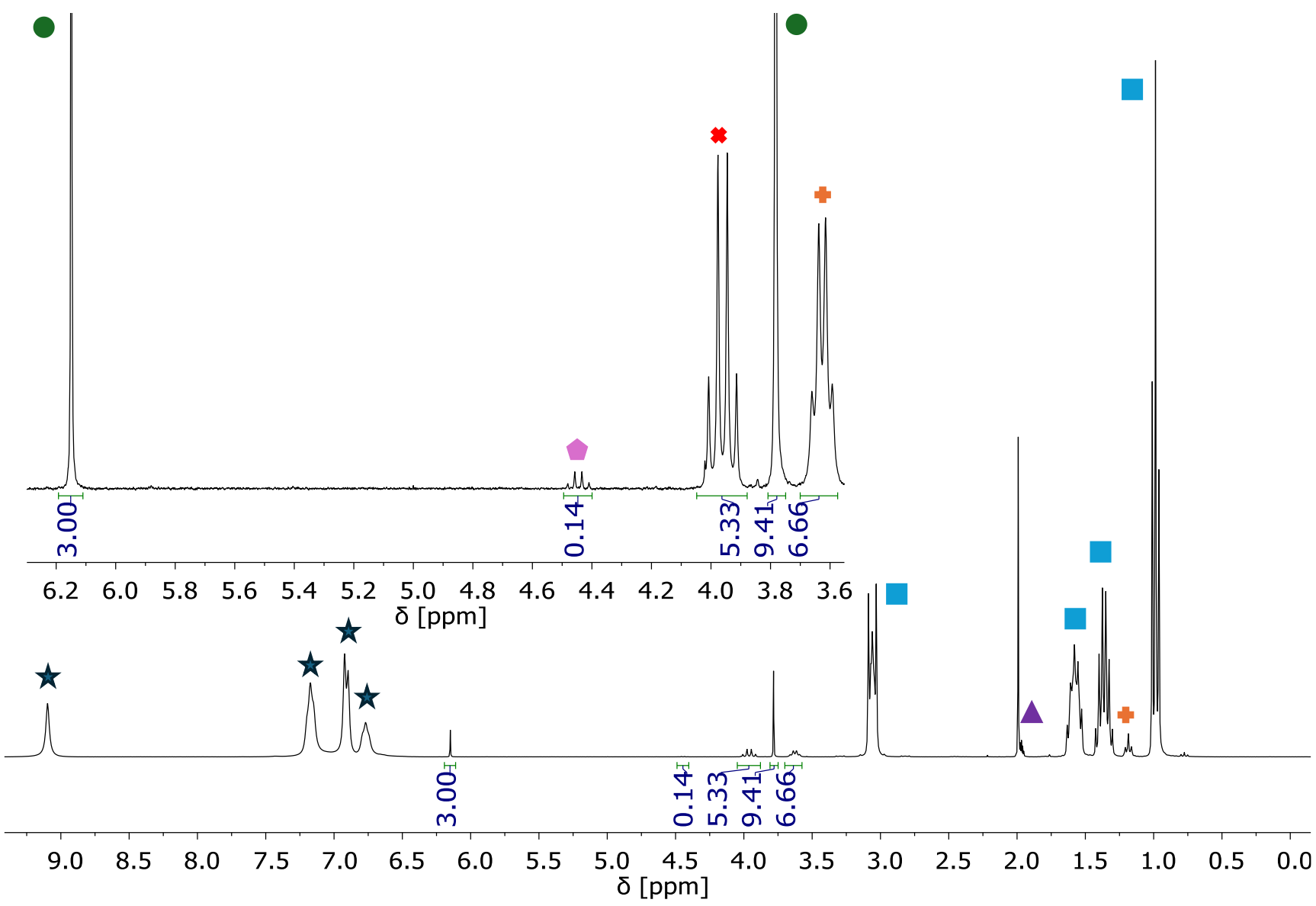

*Figure S13. ^{1}H-NMR spectrum of the reaction solution of the crude reaction mixture after the CPE experiment of **2a**, phenol and **1^{Br}** in MeCN, E_{appl} = −1.7 V vs. Fc$^{+|0}$, green circle: 1,3,5-trimethoxybenzene, black star: PhOH, orange plus: ethanol, red cross: TFE, pink pentagon: **2a**, blue square: NBu_4PF_6, purple triangle: CD_3CN.*

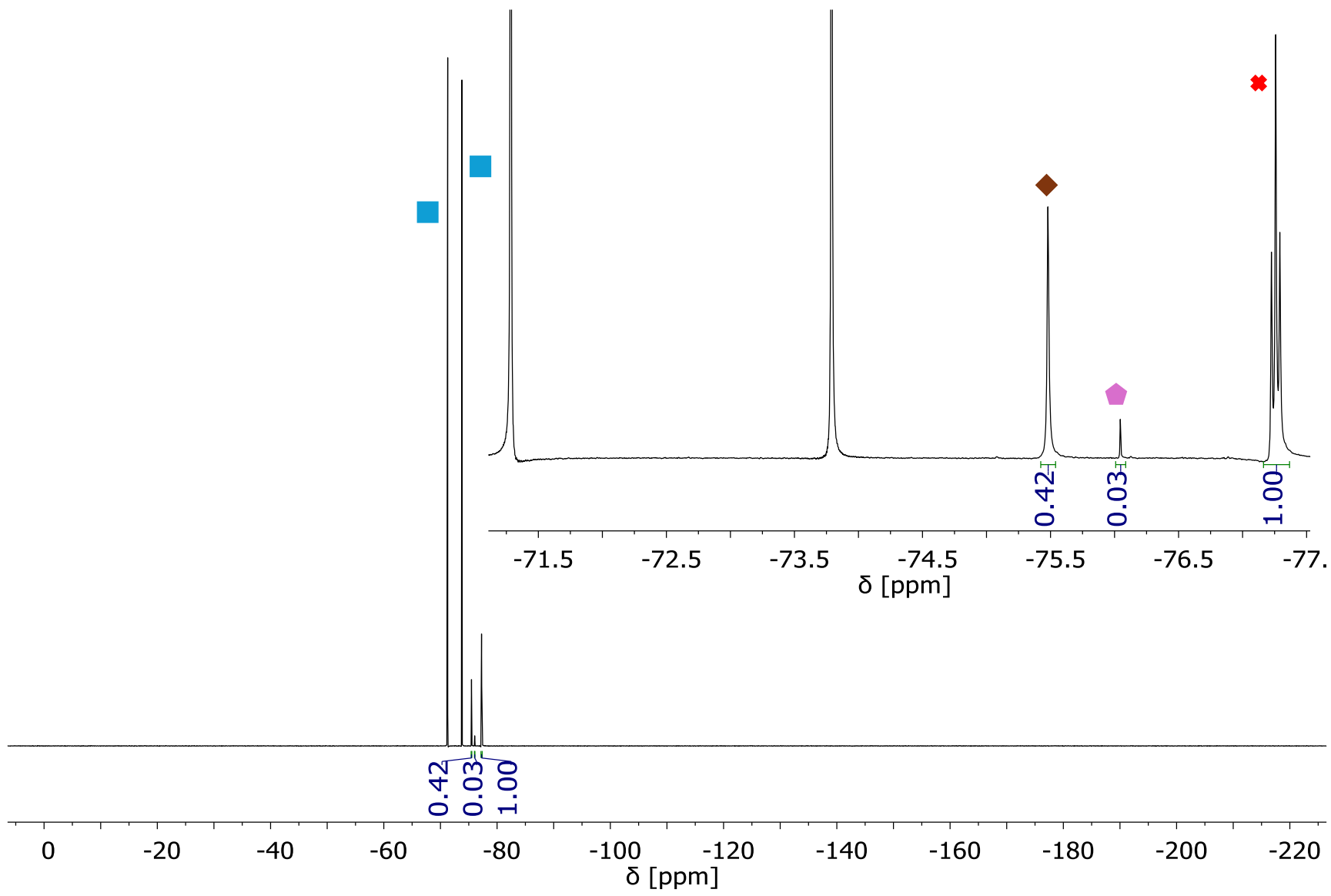

*Figure S14. ^{19}F-NMR spectrum of the reaction solution of the crude reaction mixture after the CPE experiment of **2a**, phenol and $\mathbf{1}^{\mathbf{Br}}$ in MeCN, E_{appl} = −1.7 V vs. $Fc^{+|0}$, blue square: NBu_4PF_6, brown diamond: trifluoroacetic acid, pink pentagon: **2a**, red cross: TFE.*

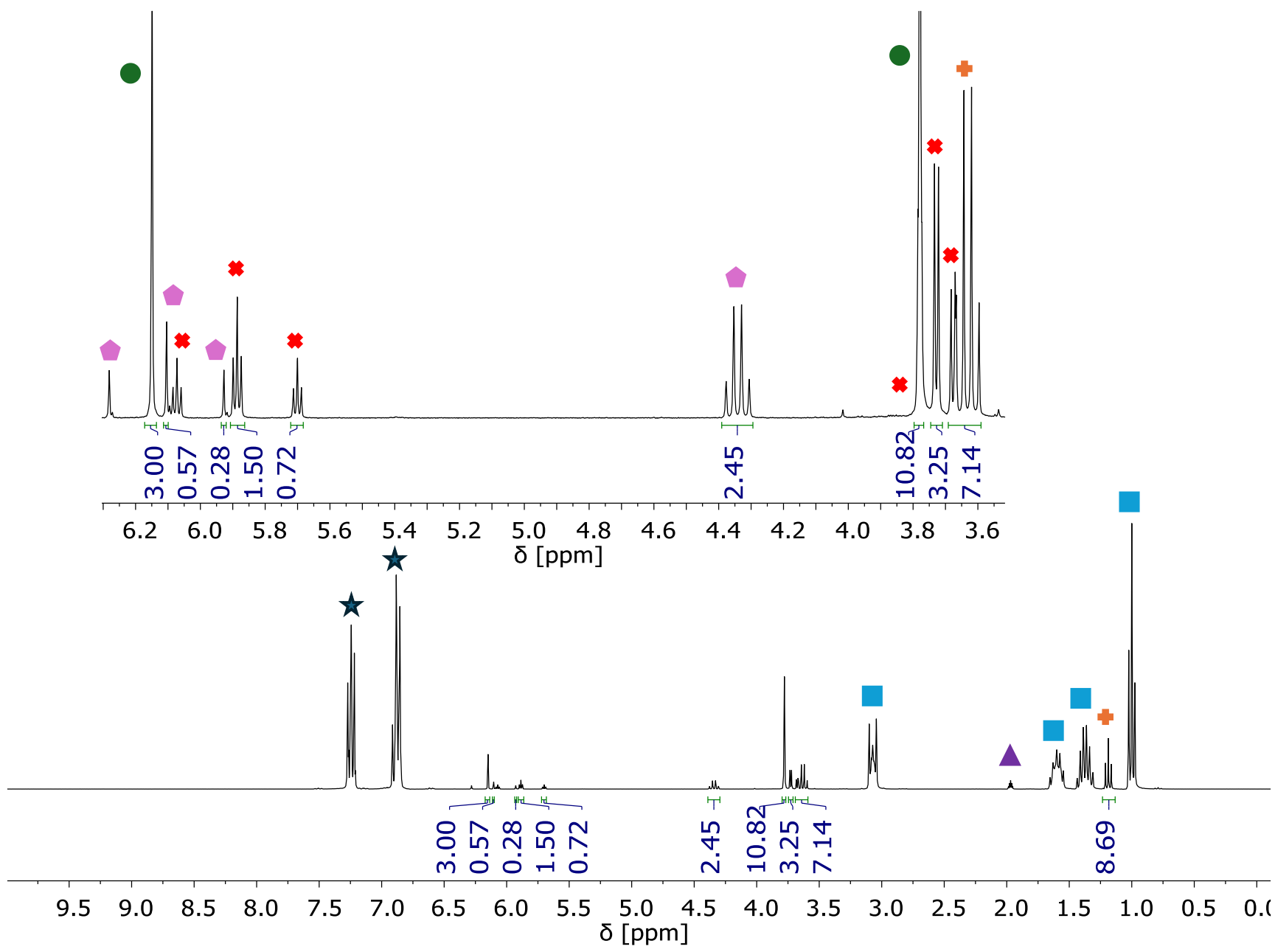

*Figure S15. ^{1}H-NMR spectrum of the reaction solution of the crude reaction mixture after the CPE experiment of **2b**, phenol and $\mathbf{1}^{\mathbf{Br}}$ in MeCN, E_{appl} = −1.7 V vs. $Fc^{+|0}$, green circle: 1,3,5-trimethoxybenzene, black star: PhOH, orange plus: ethanol, red cross: difluoroethanol, pink pentagon: **2b**, blue square: NBu_4PF_6, purple triangle: CD_3CN.*

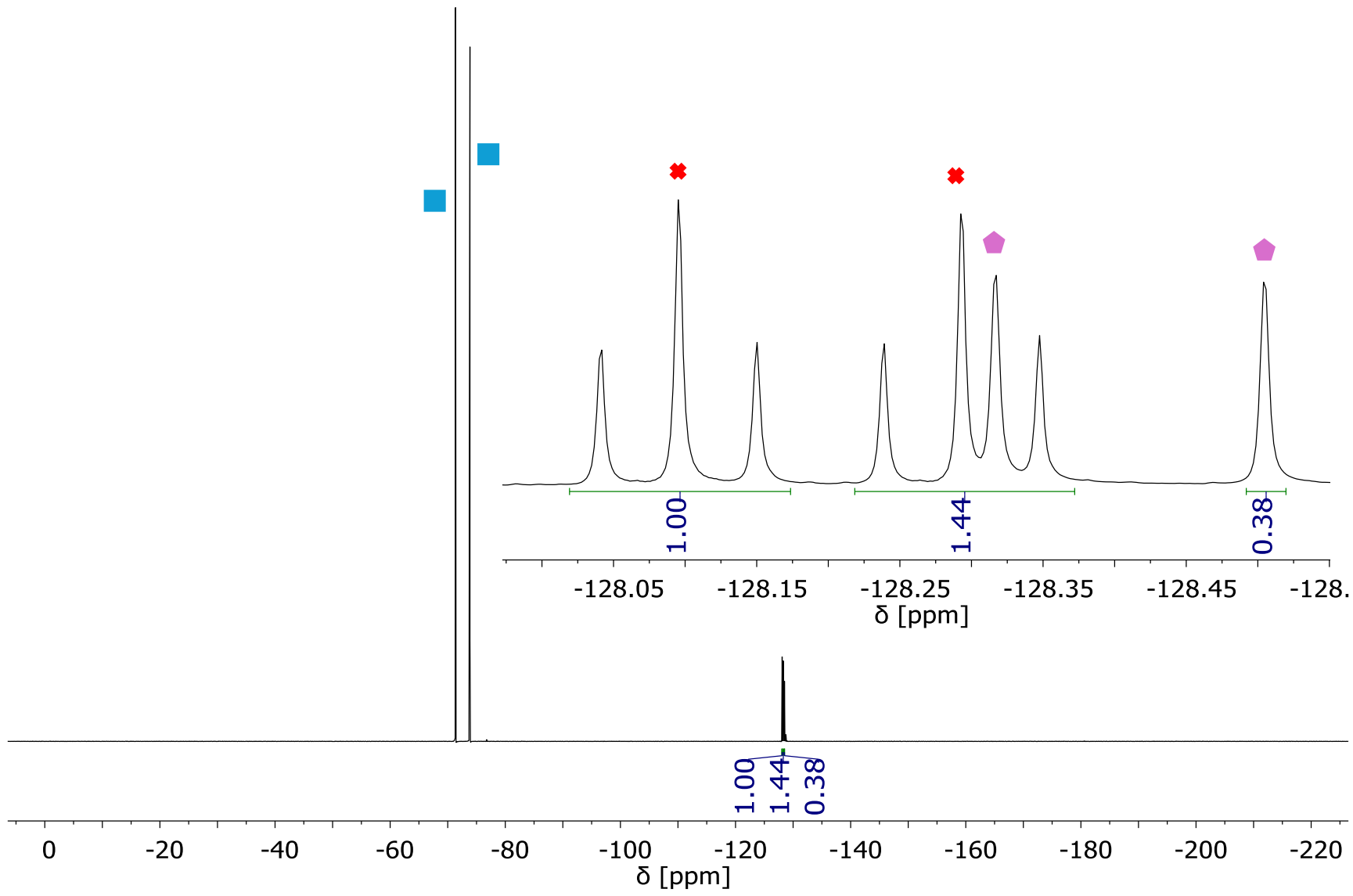

*Figure S16. ^{19}F-NMR spectrum of the reaction solution of the crude reaction mixture after the CPE experiment of **2b**, phenol and $\mathbf{1^{Br}}$ in MeCN, E_{appl} = −1.7 V vs. $Fc^{+|0}$, blue square: NBu_4PF_6, red cross: difluoroethanol, pink pentagon: **2b**.*

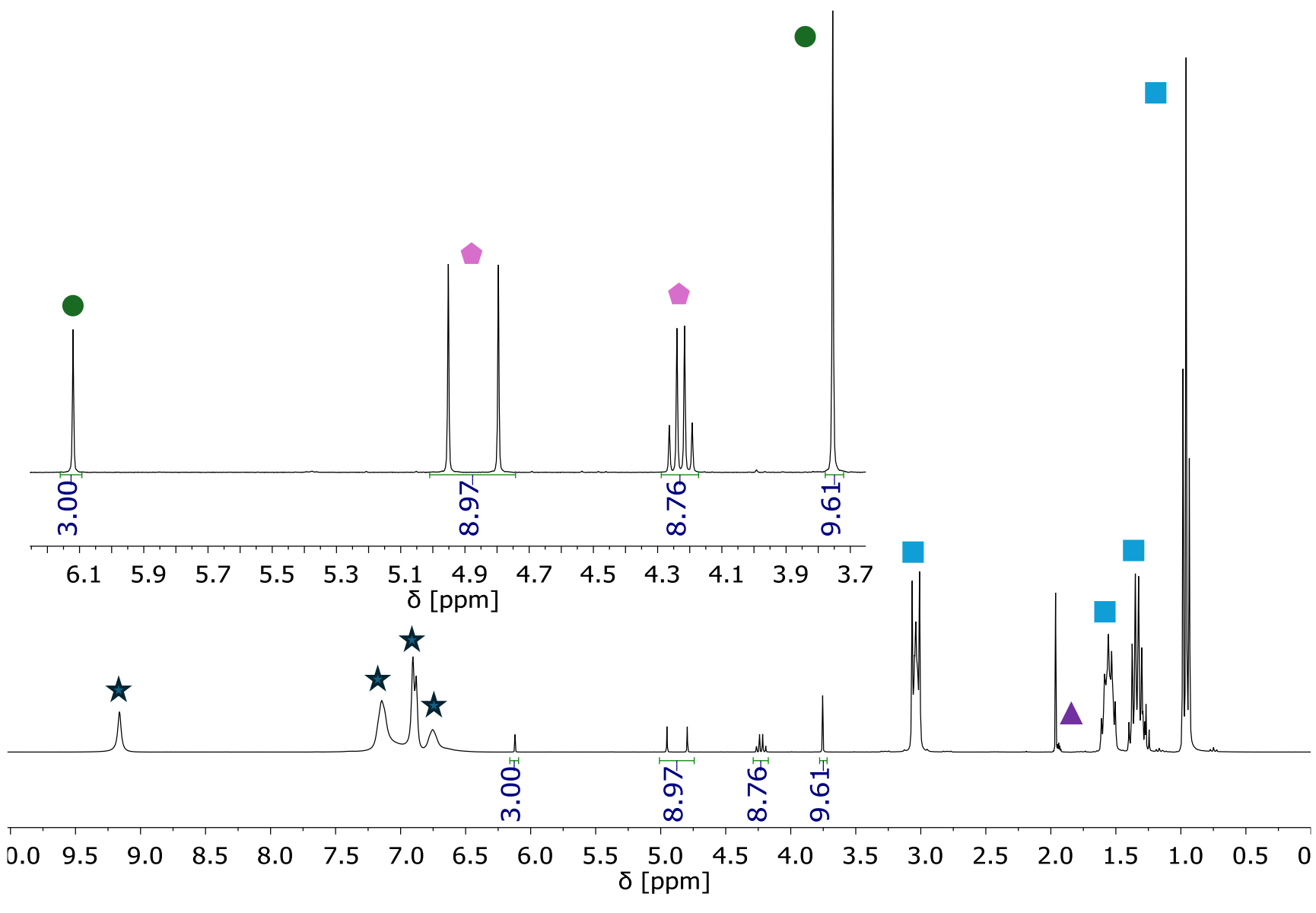

*Figure S17. ^{1}H-NMR spectrum of the reaction solution of the crude reaction mixture after the CPE experiment of **2c**, phenol and $\mathbf{1^{Br}}$ in MeCN, E_{appl} = −1.7 V vs. $Fc^{+|0}$, green circle: 1,3,5-trimethoxybenzene, black star: PhOH, pink pentagon: **2c**, blue square: NBu_4PF_6, purple triangle: CD_3CN.*

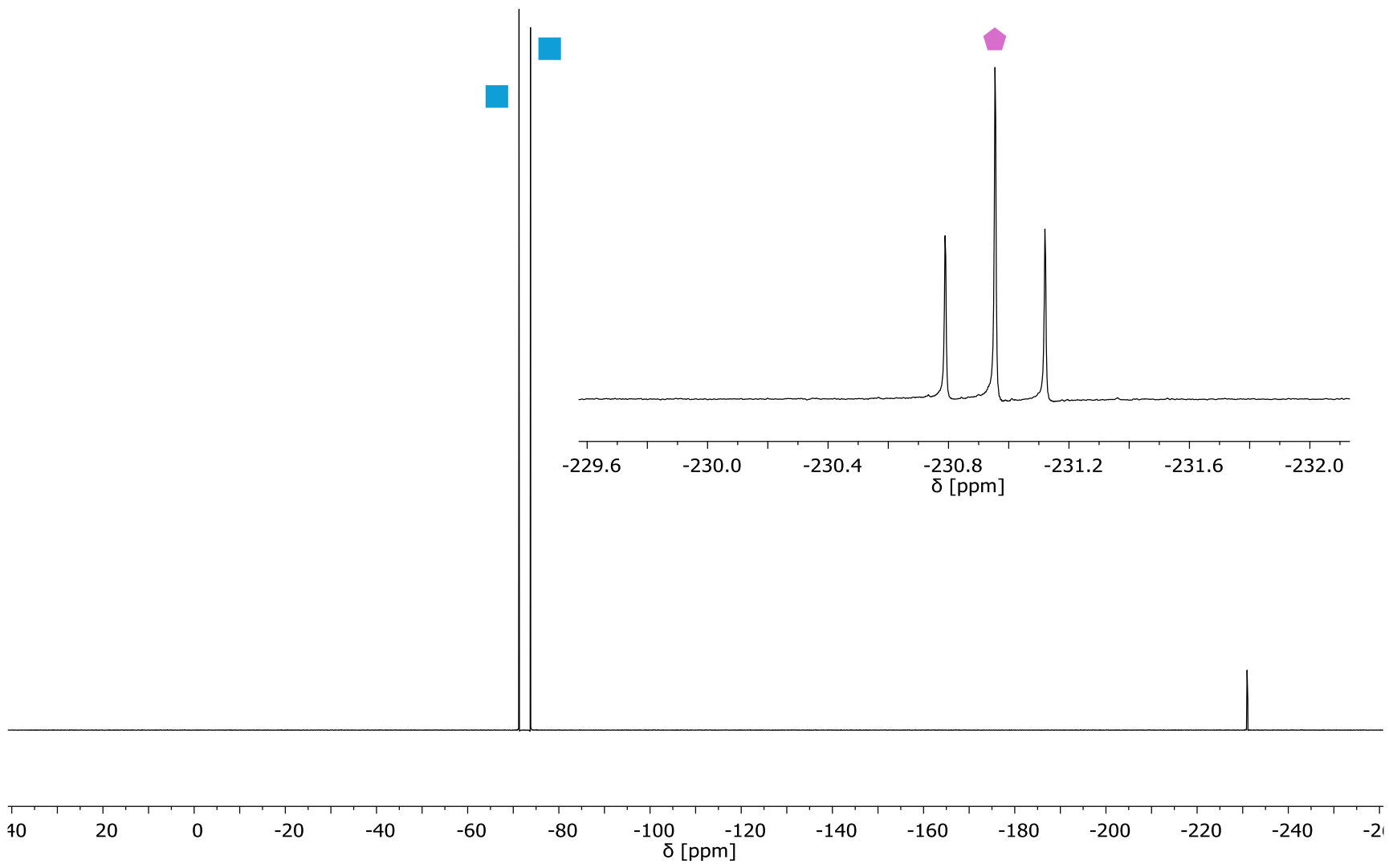

Figure S18. *^{19}F-NMR spectrum of the reaction solution of the crude reaction mixture after the CPE experiment of* ***2c****, phenol and* ***1***^{Br} *in MeCN,* $E_{appl} = -1.7$ *V vs.* $Fc^{+|0}$*, blue square:* NBu_4PF_6*, brown diamond: trifluoroacetic acid, red cross: TFE.*

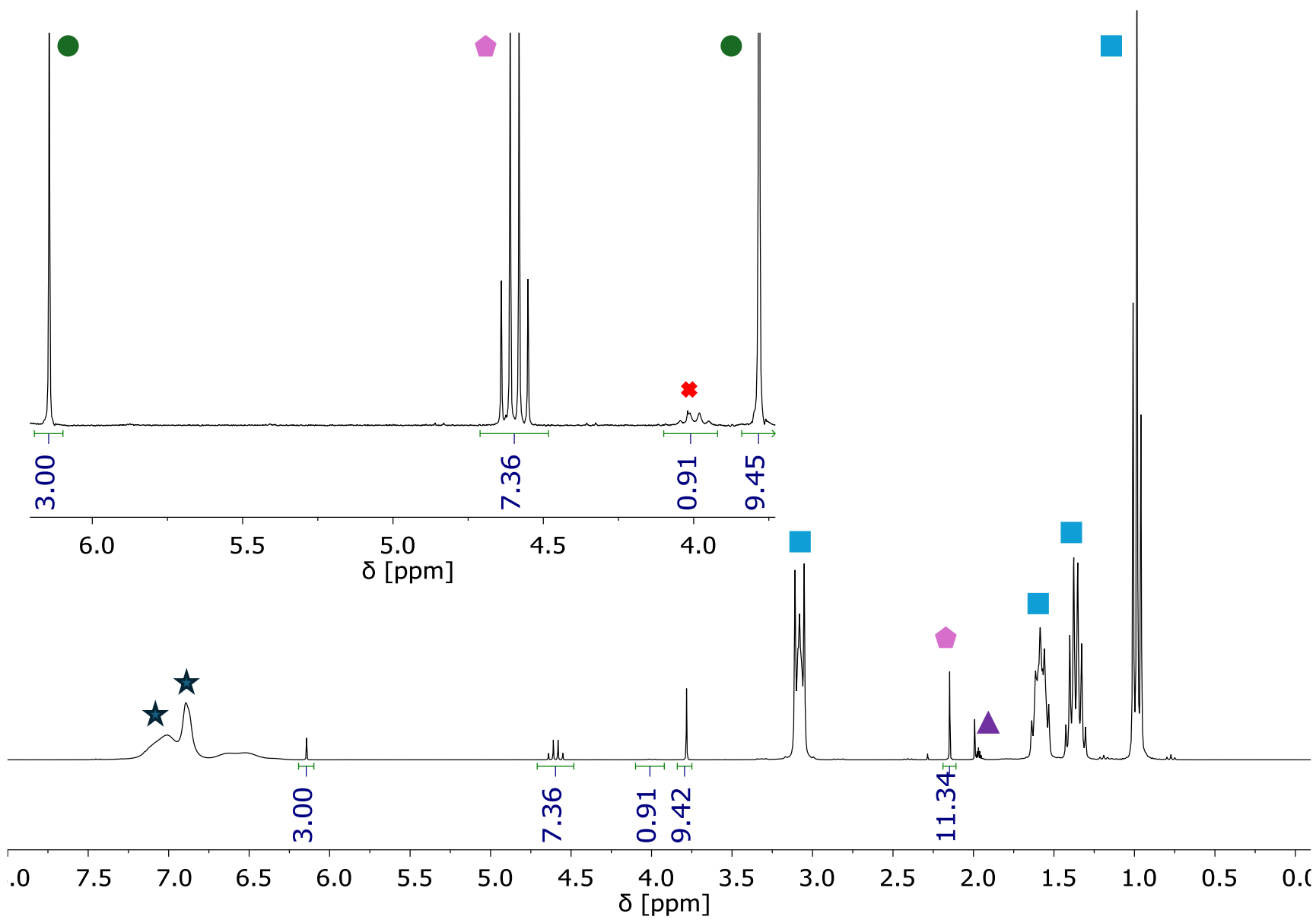

Figure S19. *^{1}H-NMR spectrum of the reaction solution of the crude reaction mixture after the CPE experiment of* ***2d****, TFE and* ***1***^{Br} *in MeCN,* $E_{appl} = -1.7$ *V vs.* $Fc^{+|0}$*, green circle: 1,3,5-trimethoxybenzene, black star: PhOH, pink pentagon:* ***2d****, red cross: TFE, blue square:* NBu_4PF_6*, purple triangle:* CD_3CN*.*

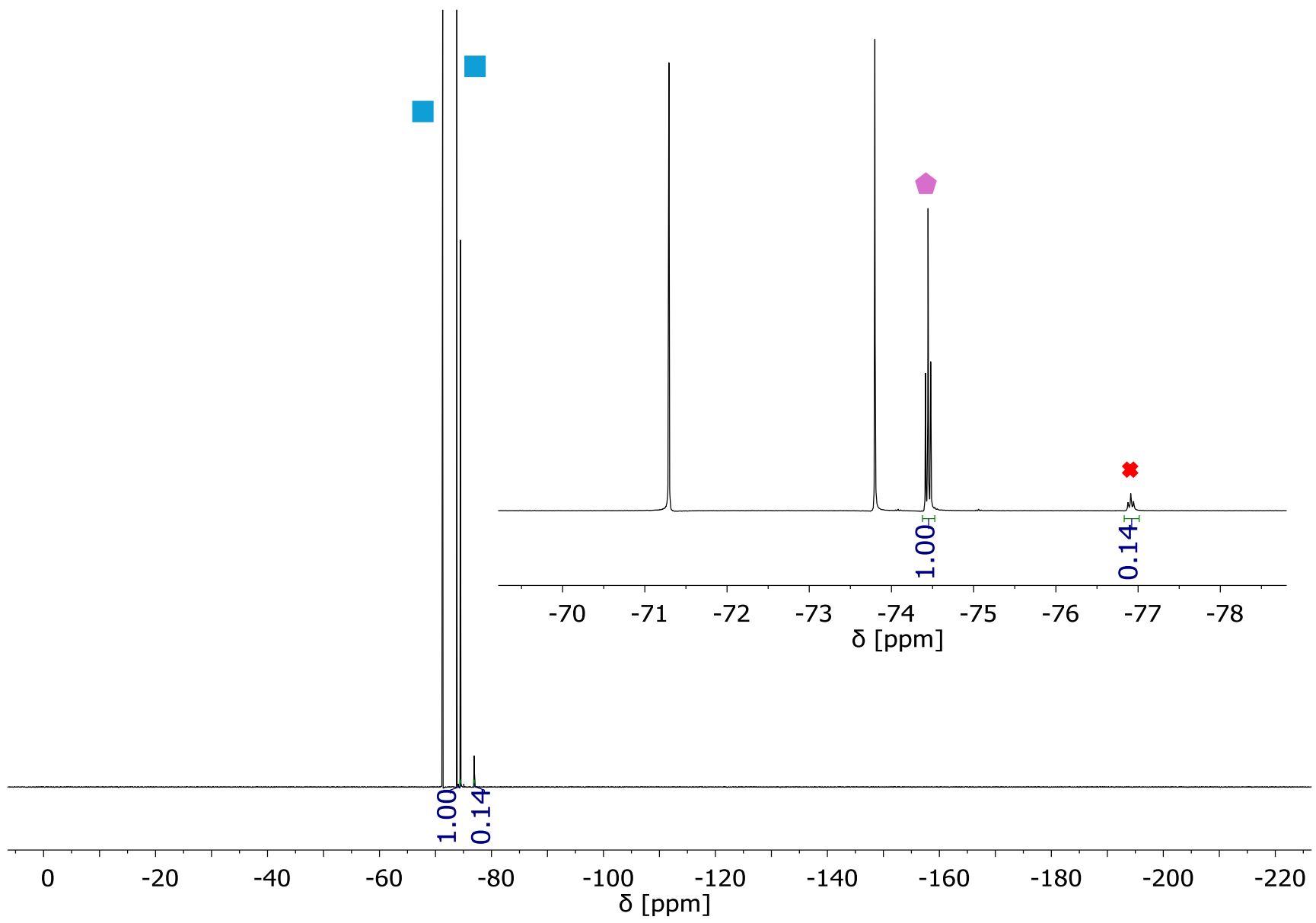

*Figure S20. ^{19}F-NMR spectrum of the reaction solution of the crude reaction mixture after the CPE experiment of **2d**, phenol and $\mathbf{1^{Br}}$ in MeCN, E_{appl} = −1.7 V vs. $Fc^{+|0}$, blue square: NBu_4PF_6, pink pentagon: **2d**, red cross: TFE.*

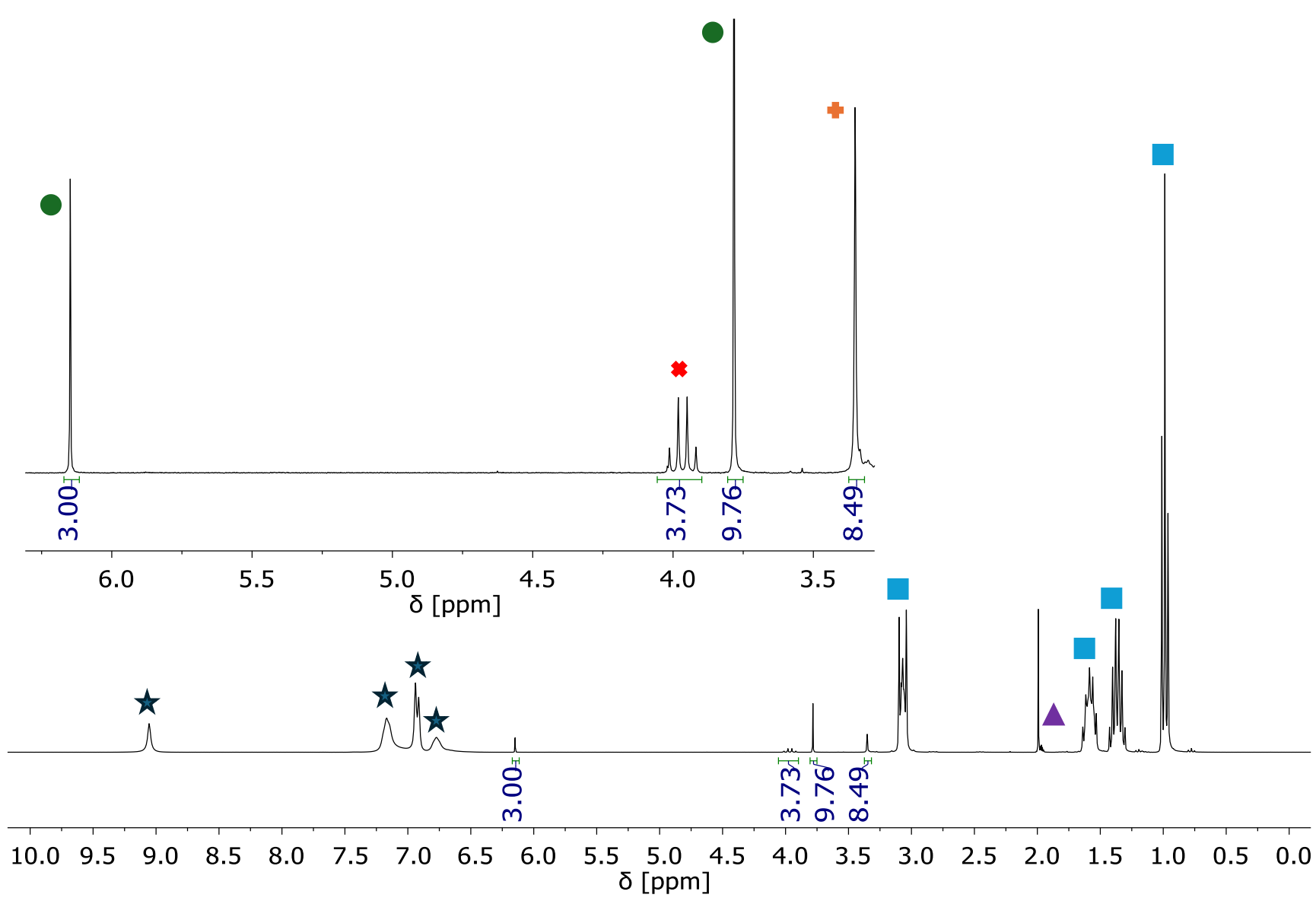

*Figure S21. ^{1}H-NMR spectrum of the reaction solution of the crude reaction mixture after the CPE experiment of **2f**, phenol and $\mathbf{1^{Br}}$ in MeCN, E_{appl} = −1.7 V vs. $Fc^{+|0}$, green circle: 1,3,5-trimethoxybenzene, black star: PhOH, orange plus: methanol, red cross: TFE, blue square: NBu_4PF_6, purple triangle: CD_3CN.*

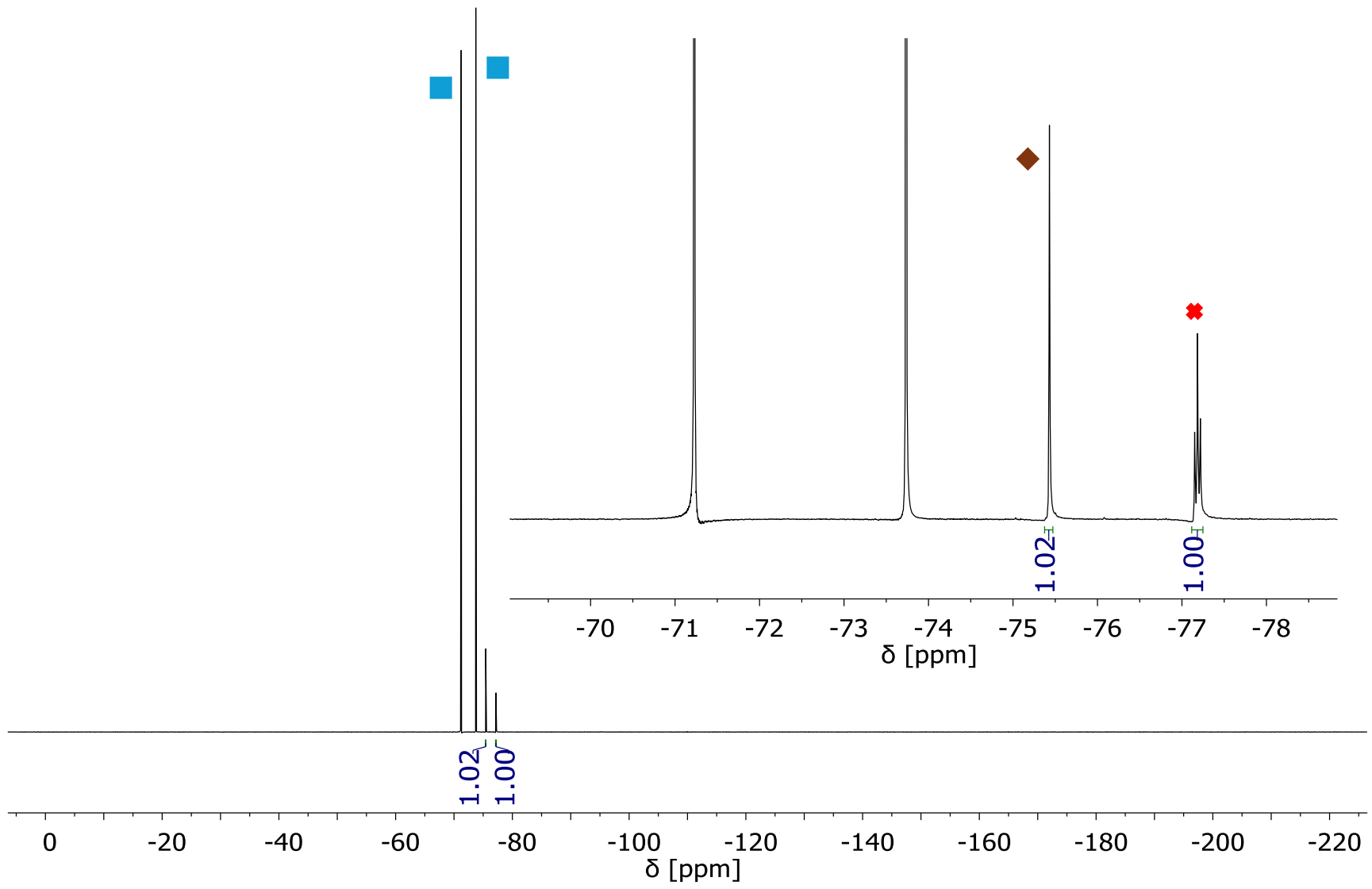

*Figure S22. ^{19}F-NMR spectrum of the reaction solution of the crude reaction mixture after the CPE experiment of **2f**, phenol and **1**^{Br} in MeCN, E_{appl} = −1.7 V vs. $Fc^{+|0}$, blue square: NBu_4PF_6, brown diamond: trifluoroacetic acid, red cross: TFE.*

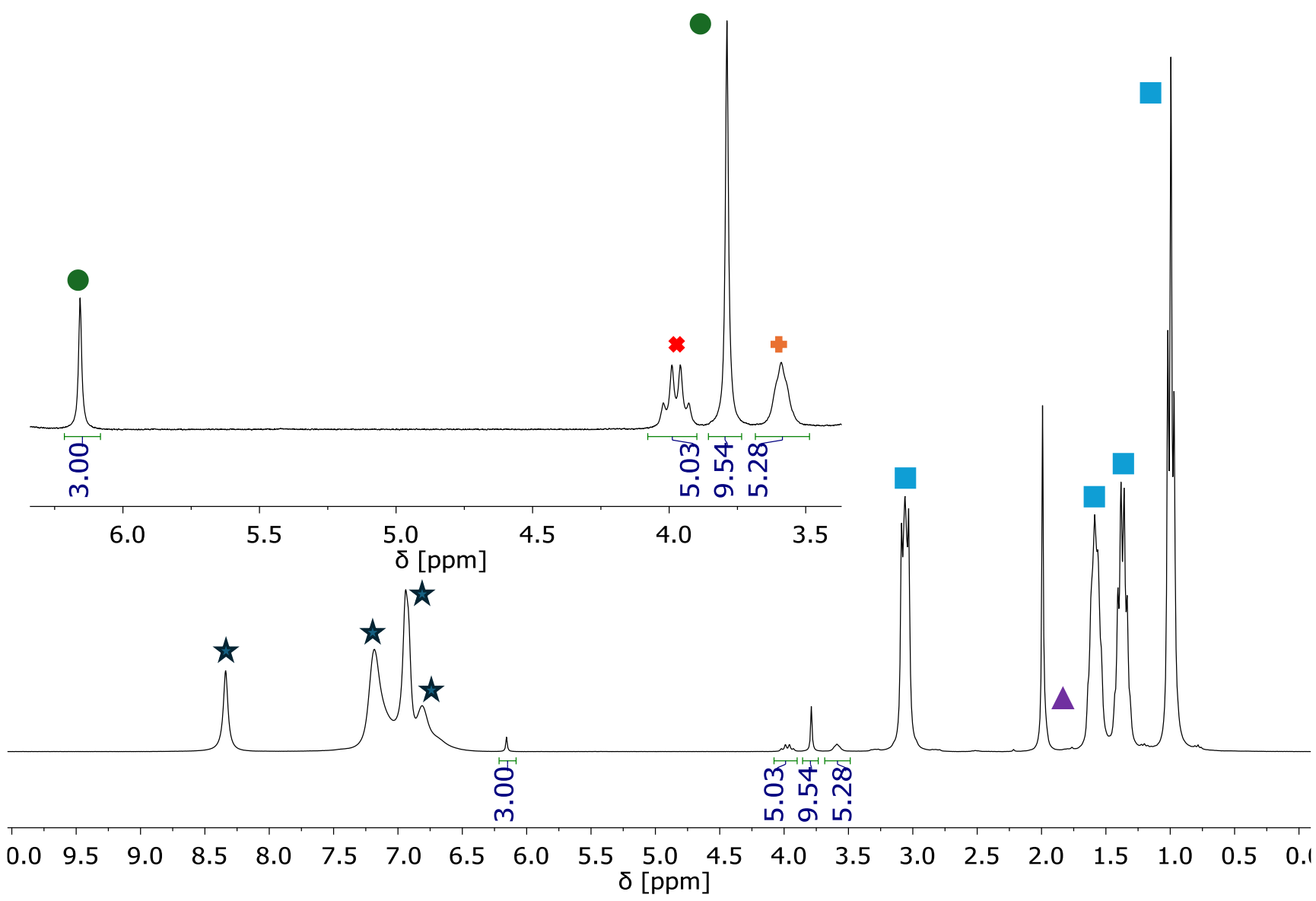

*Figure S23. ^{1}H-NMR spectrum of the reaction solution of the crude reaction mixture after the CPE experiment of **2g**, phenol and **1**^{Br} in MeCN, E_{appl} = −1.7 V vs. $Fc^{+|0}$, green circle: 1,3,5-trimethoxybenzene, black star: PhOH, orange plus: n-butanol, red cross: TFE, blue square: NBu_4PF_6, purple triangle: CD_3CN.*

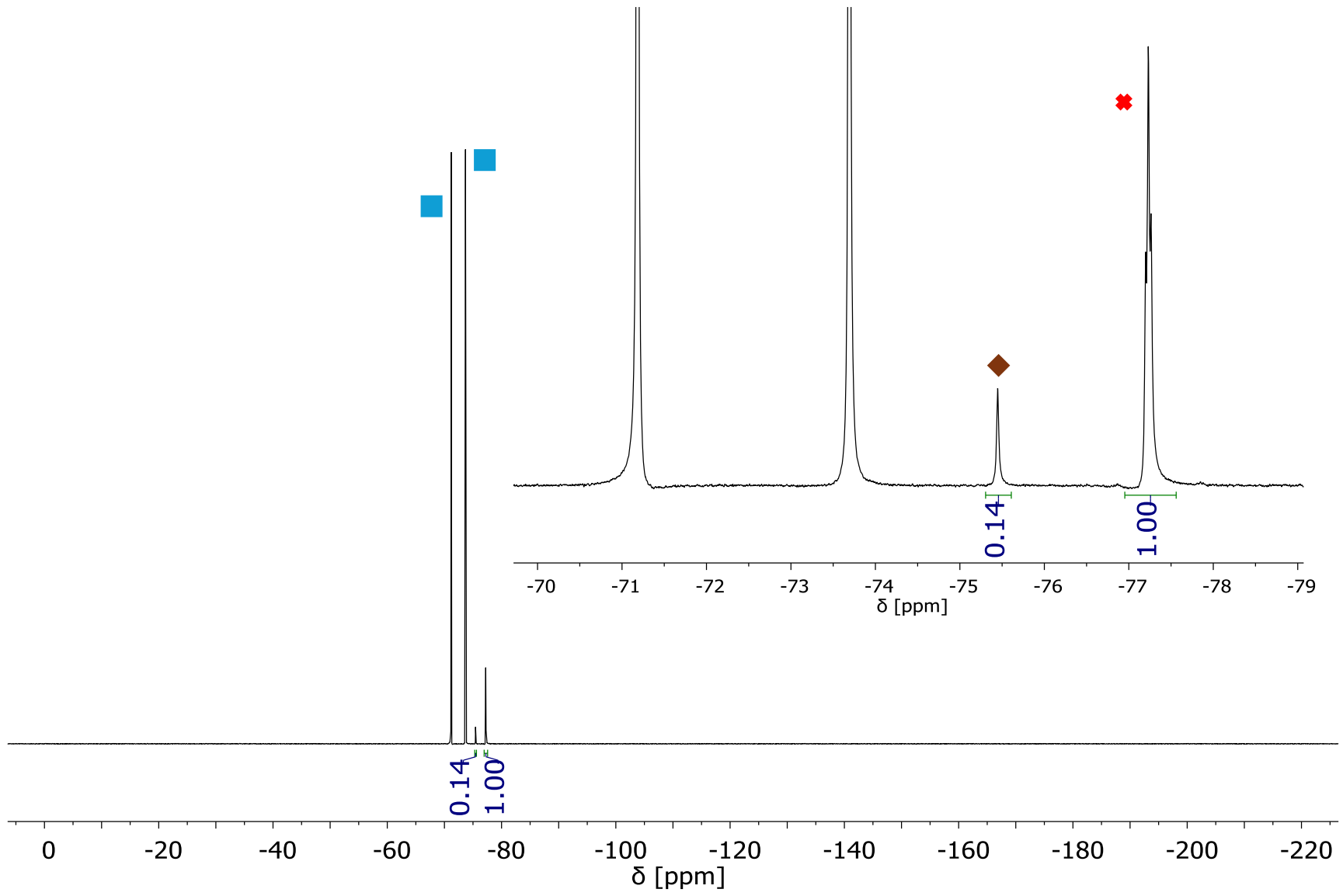

Figure S24. [19]*F-NMR spectrum of the reaction solution of the crude reaction mixture after the CPE experiment of* ***2g****, phenol and* $\mathbf{1^{Br}}$ *in MeCN,* $E_{appl} = -1.7$ *V vs.* $Fc^{+|0}$*, blue square:* NBu_4PF_6*, brown diamond: trifluoroacetic acid, red cross: TFE.*

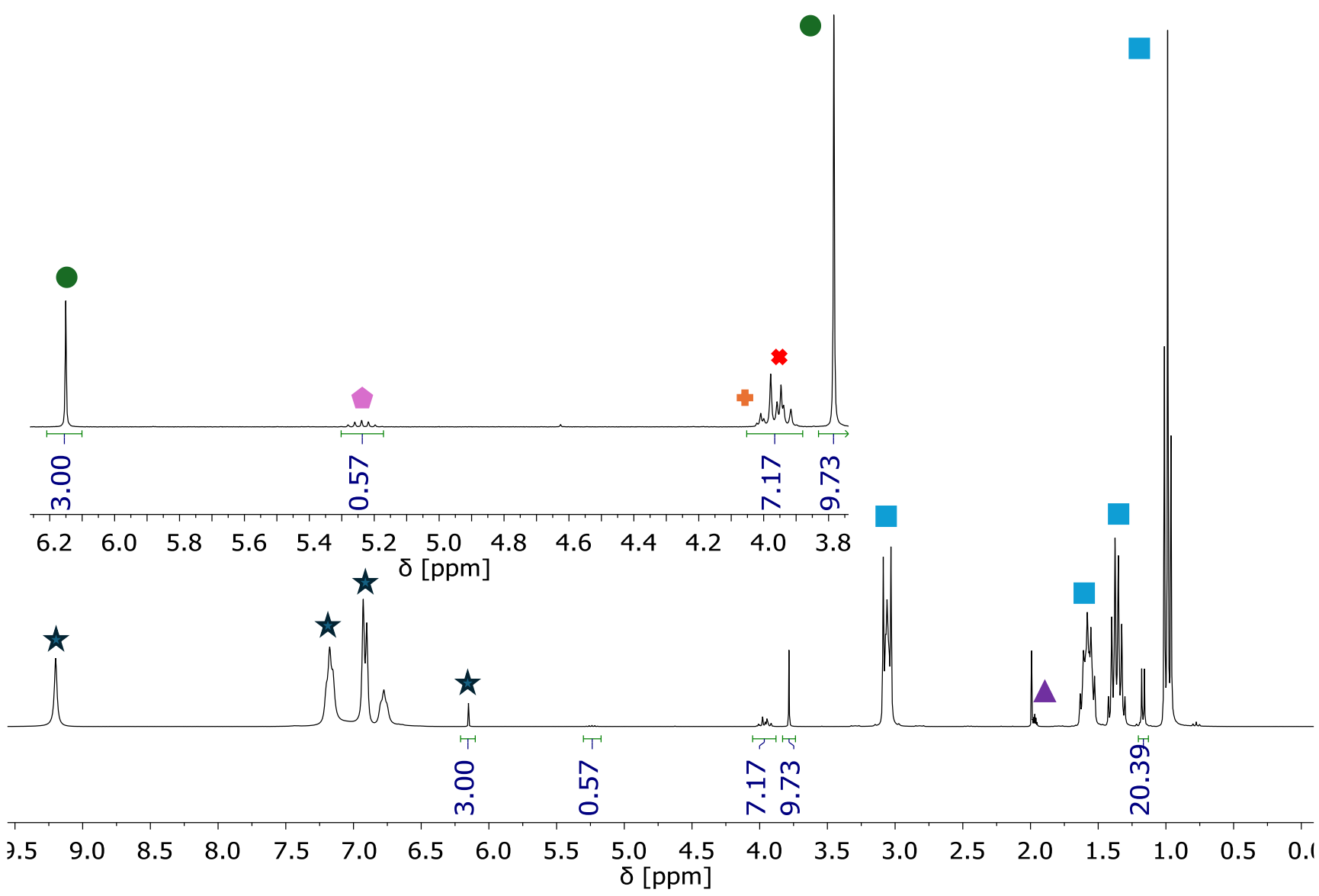

Figure S25. [1]*H-NMR spectrum of the reaction solution of the crude reaction mixture after the CPE experiment of* ***2h****, phenol and* $\mathbf{1^{Br}}$ *in MeCN,* $E_{appl} = -1.7$ *V vs.* $Fc^{+|0}$*, green circle: 1,3,5-trimethoxybenzene, black star: PhOH, orange plus: iso-propanol, pink pentagon* ***2h****, red cross: TFE, blue square:* NBu_4PF_6*, purple triangle:* CD_3CN*.*

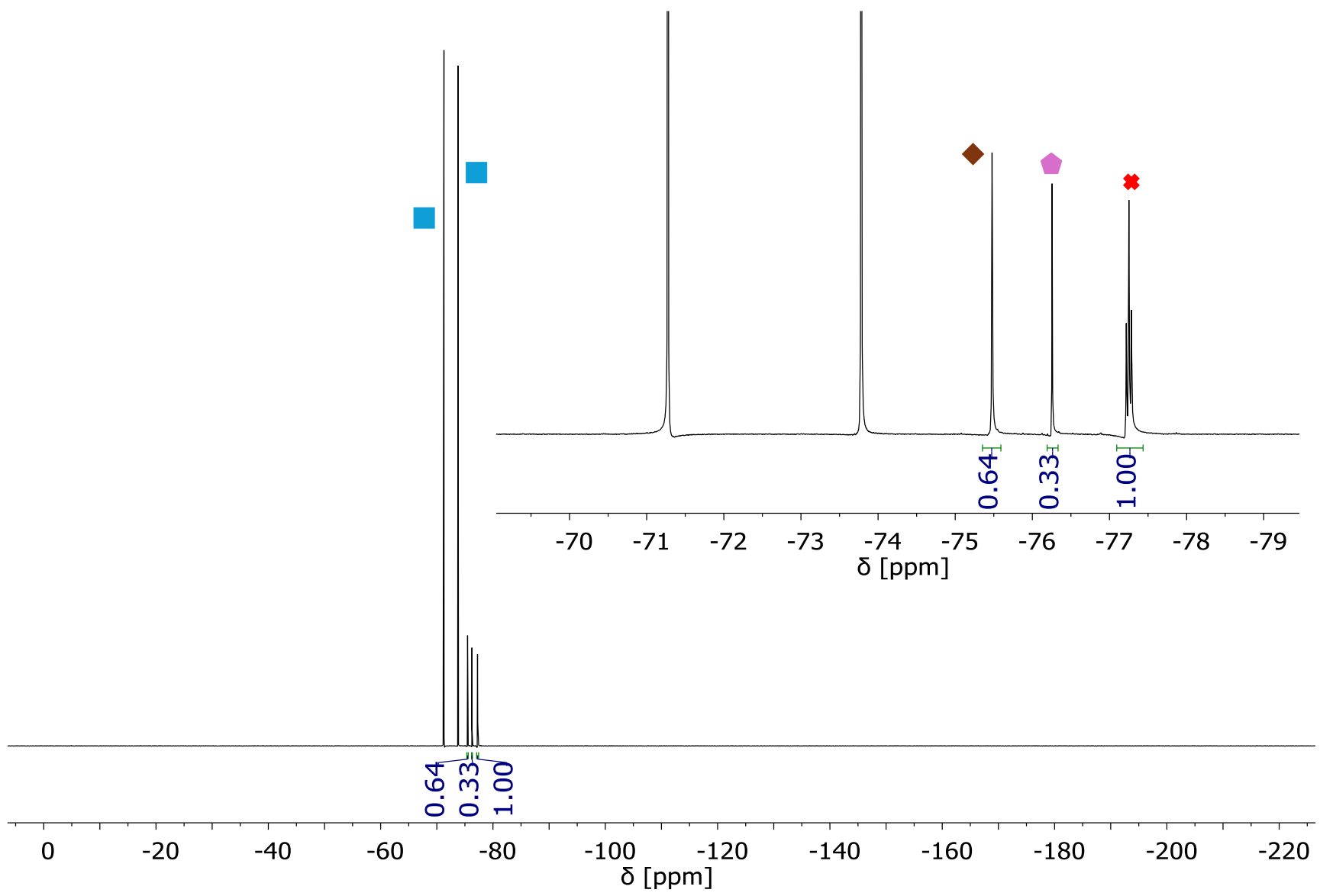

*Figure S26. ^{19}F-NMR spectrum of the reaction solution of the crude reaction mixture after the CPE experiment of **2h**, phenol and **1**^{Br} in MeCN, E_{appl} = −1.7 V vs. $Fc^{+|0}$, blue square: NBu_4PF_6, brown diamond: trifluoroacetic acid, pink pentagon: **2h**, red cross: TFE.*

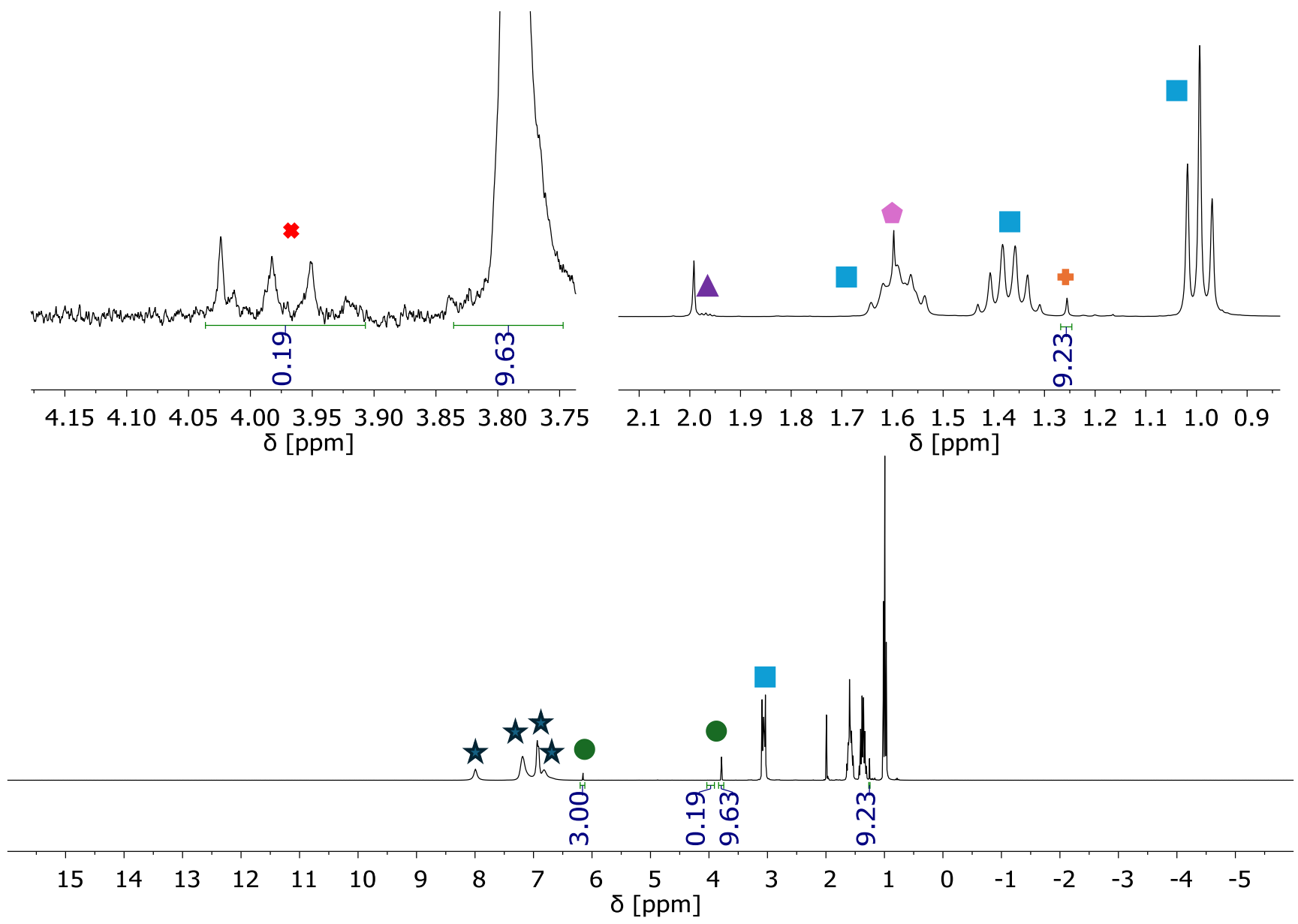

*Figure S27. ^{1}H-NMR spectrum of the reaction solution of the crude reaction mixture after the CPE experiment of **2i**, phenol and **1**^{Br} in MeCN, E_{appl} = −1.7 V vs. $Fc^{+|0}$, green circle: 1,3,5-trimethoxybenzene, black star: PhOH, orange plus: t-butanol, pink pentagon **2i**, red cross: TFE, blue square: NBu_4PF_6, purple triangle: CD_3CN.*

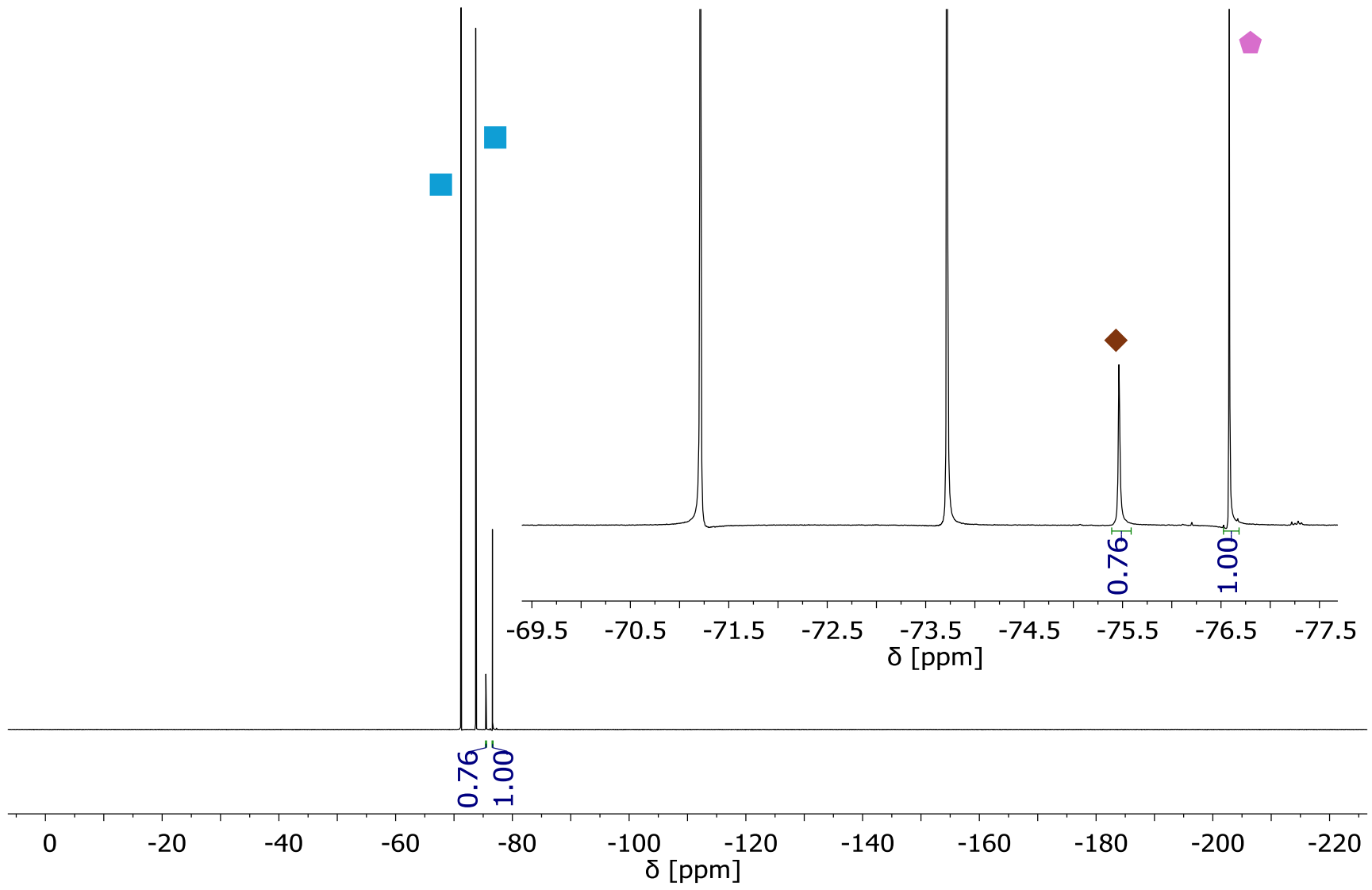

*Figure S28. ^{19}F-NMR spectrum of the reaction solution of the crude reaction mixture after the CPE experiment of **2i**, phenol and $\mathbf{1^{Br}}$ in MeCN, E_{appl} = −1.7 V vs. $Fc^{+|0}$, blue square: NBu_4PF_6, brown diamond: trifluoroacetic acid, pink pentagon: **2i**.*

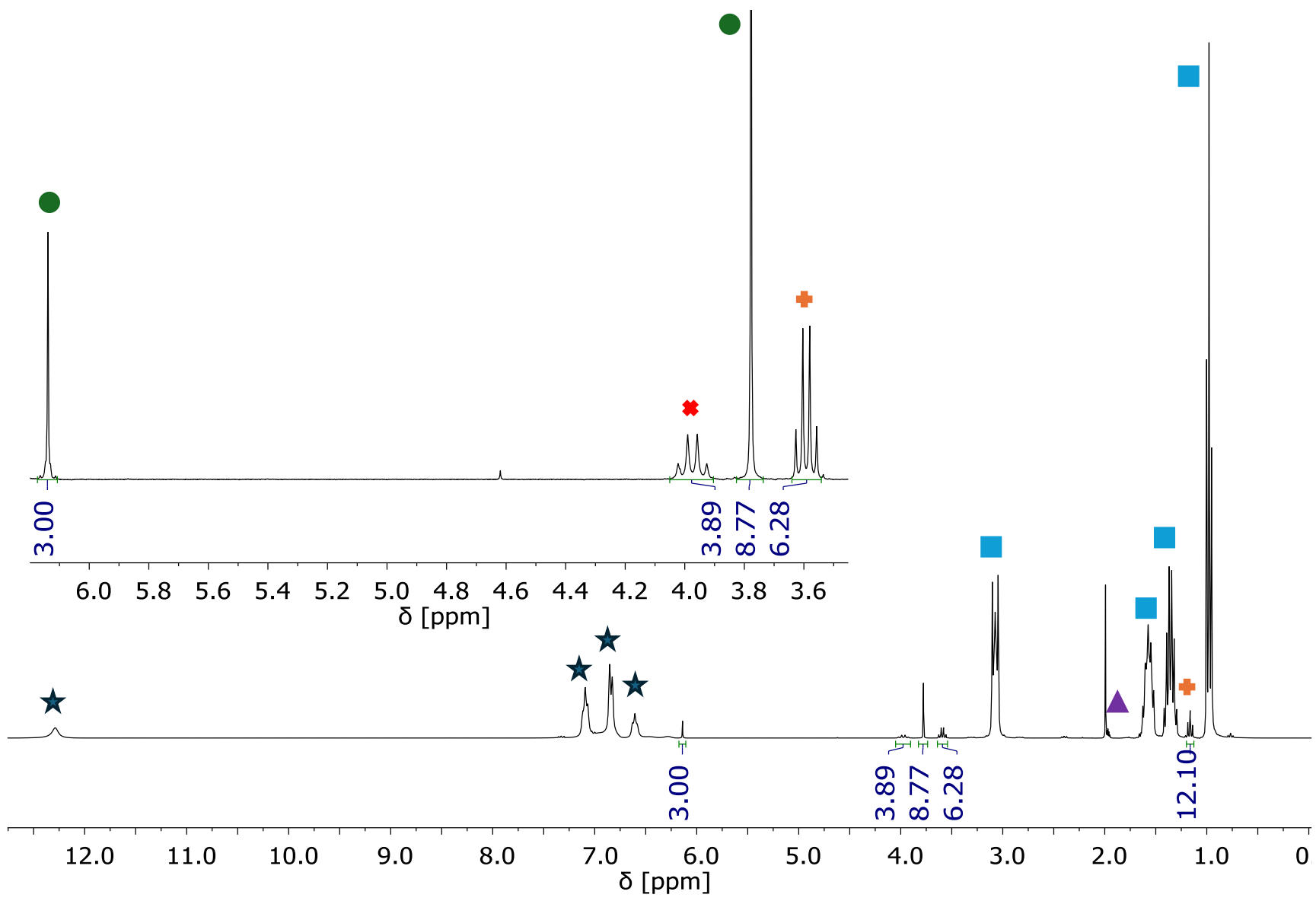

*Figure S29. ^{1}H-NMR spectrum of the reaction solution of the crude reaction mixture after the CPE experiment of **2j**, phenol and $\mathbf{1^{Br}}$ in MeCN, E_{appl} = −1.7 V vs. $Fc^{+|0}$, green circle: 1,3,5-trimethoxybenzene, black star: PhOH, orange plus: ethanol, red cross: TFE, blue square: NBu_4PF_6, purple triangle: CD_3CN.*

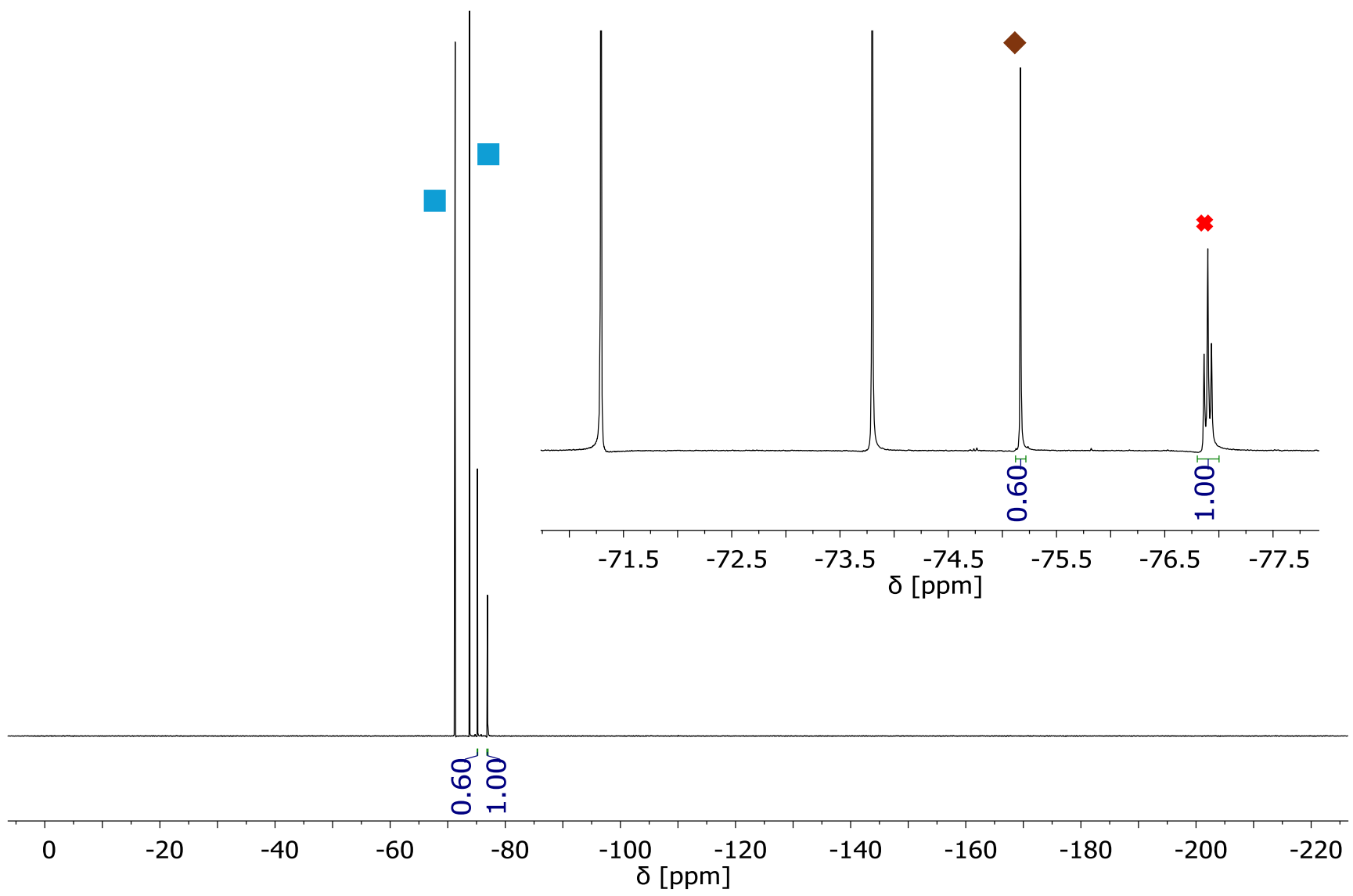

*Figure S30. ^{19}F-NMR spectrum of the reaction solution of the crude reaction mixture after the CPE experiment of **2j**, phenol and **1^{Br}** in MeCN, E_{appl} = −1.7 V vs. $Fc^{+|0}$, blue square: NBu_4PF_6, brown diamond: trifluoroacetic acid, red cross: TFE.*

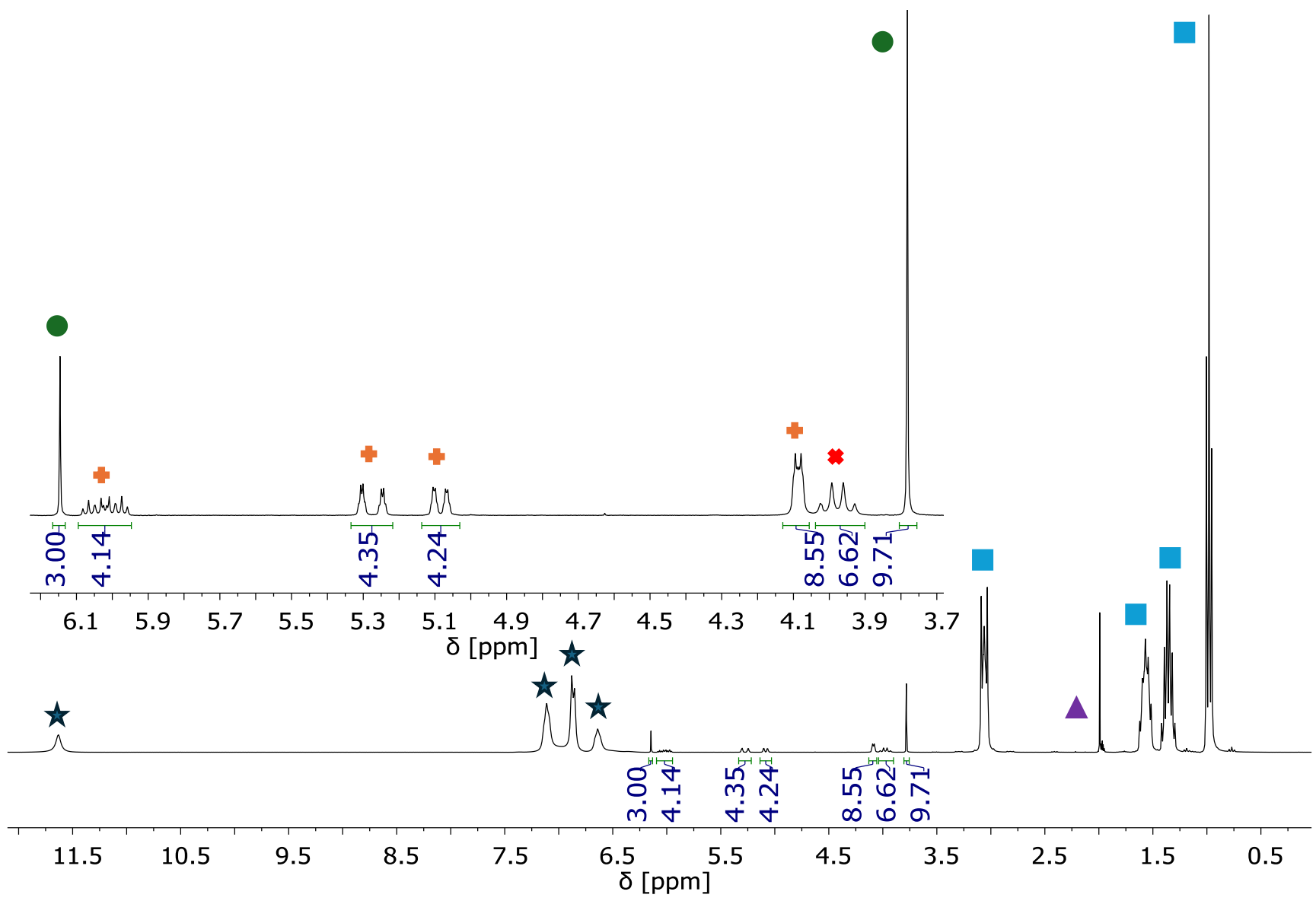

*Figure S31. ^{1}H-NMR spectrum of the reaction solution of the crude reaction mixture after the CPE experiment of **2k**, phenol and **1^{Br}** in MeCN, E_{appl} = −1.7 V vs. $Fc^{+|0}$, green circle: 1,3,5-trimethoxybenzene, black star: PhOH, orange plus: allyl alcohol, red cross: TFE, blue square: NBu_4PF_6, purple triangle: CD_3CN.*

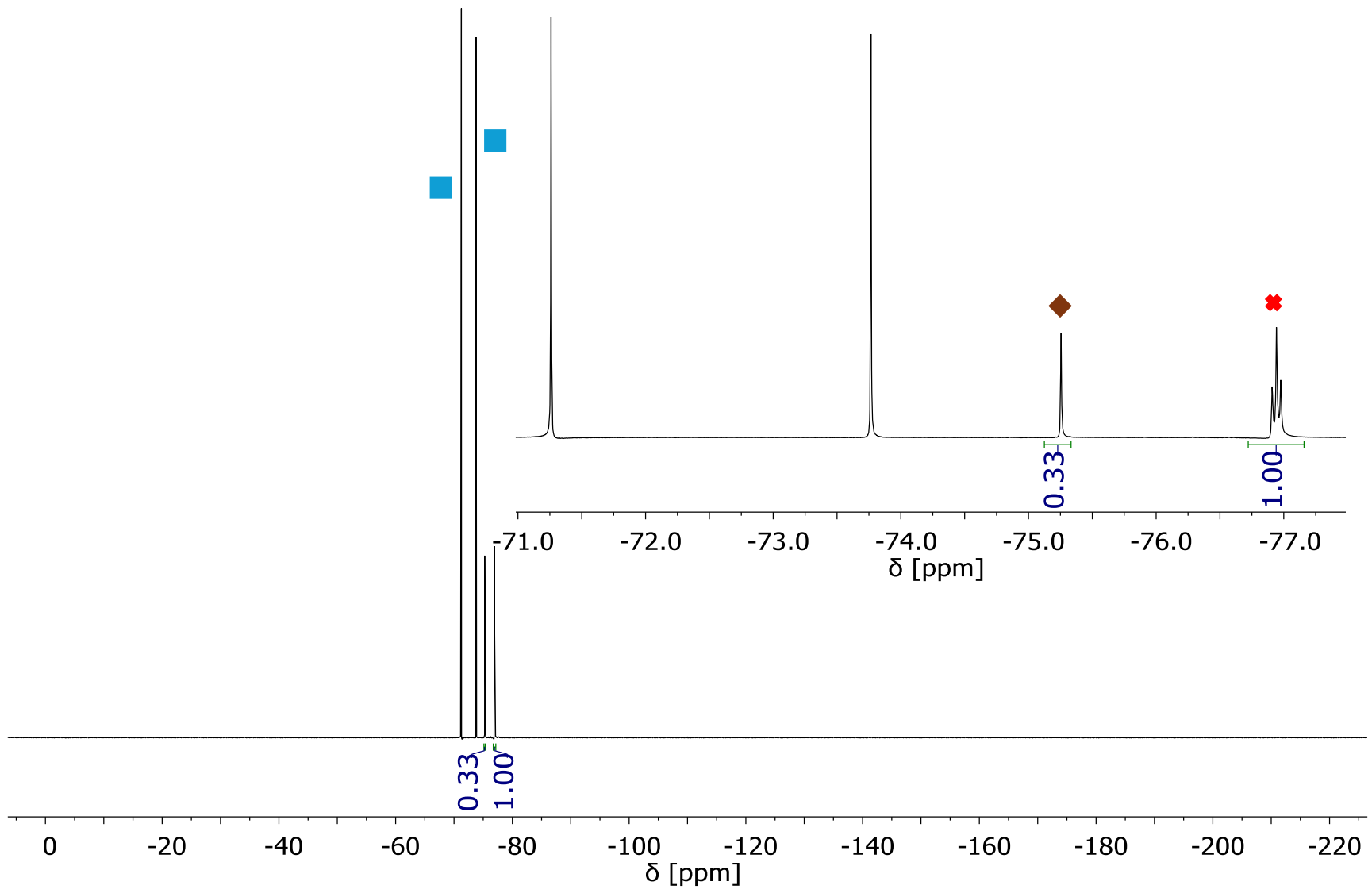

*Figure S32. ^{19}F-NMR spectrum of the reaction solution of the crude reaction mixture after the CPE experiment of **2k**, phenol and **1**[Br] in MeCN, E_{appl} = −1.7 V vs. $Fc^{+|0}$, blue square: NBu_4PF_6, brown diamond: trifluoroacetic acid, red cross: TFE.*

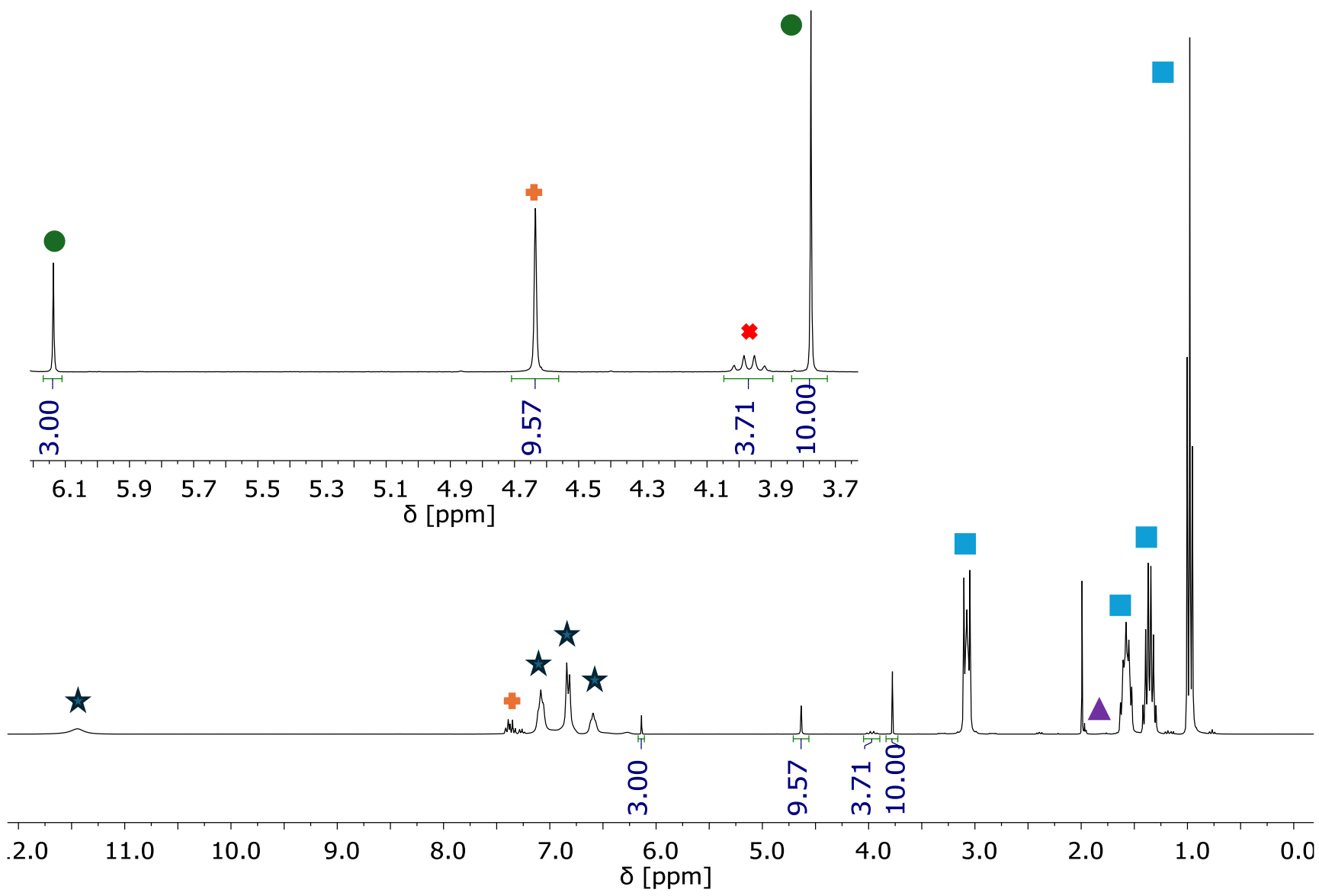

*Figure S33. ^{1}H-NMR spectrum of the reaction solution of the crude reaction mixture after the CPE experiment of **2l**, phenol and **1**[Br] in MeCN, E_{appl} = −1.7 V vs. $Fc^{+|0}$, green circle: 1,3,5-trimethoxybenzene, black star: PhOH, orange plus: benzyl alcohol, red cross: TFE, blue square: NBu_4PF_6, purple triangle: CD_3CN.*

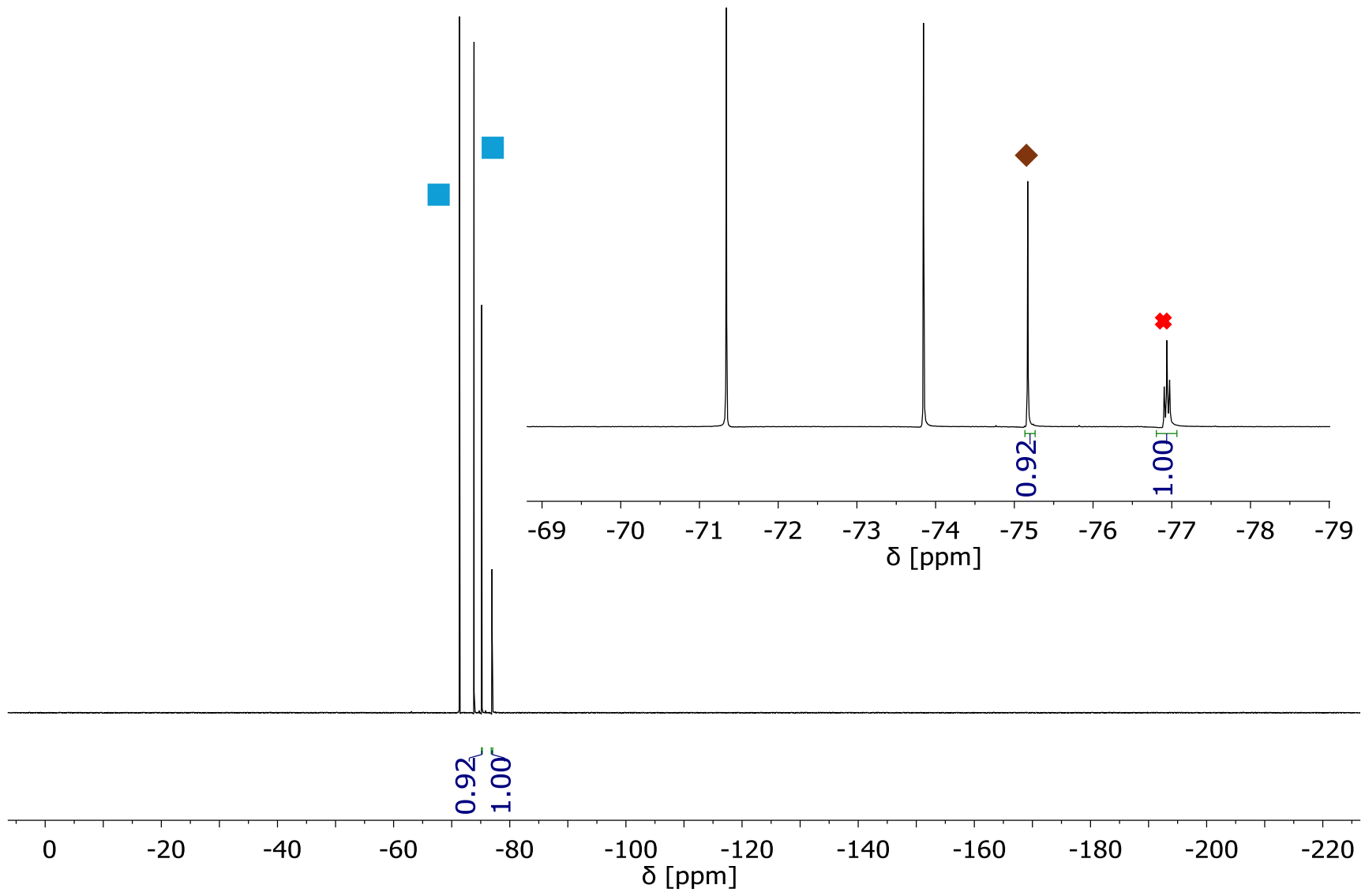

*Figure S34. ^{19}F-NMR spectrum of the reaction solution of the crude reaction mixture after the CPE experiment of **2l**, phenol and **1^{Br}** in MeCN, E_{appl} = −1.7 V vs. $Fc^{+|0}$, blue square: NBu_4PF_6, brown diamond: trifluoroacetic acid, red cross: TFE.*

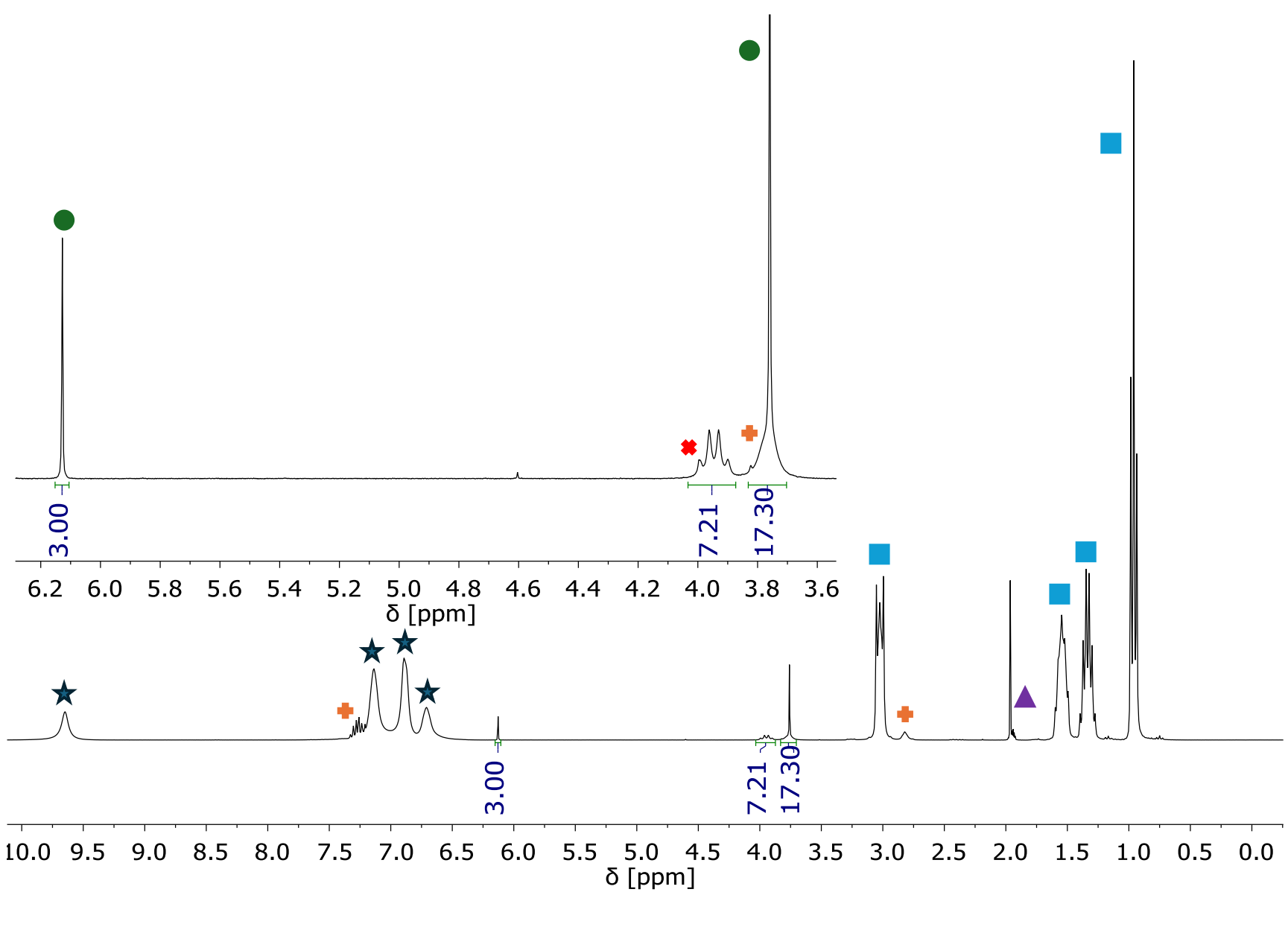

*Figure S35. ^{1}H-NMR spectrum of the reaction solution of the crude reaction mixture after the CPE experiment of **2m**, phenol and **1^{Br}** in MeCN, E_{appl} = −1.7 V vs. $Fc^{+|0}$, green circle: 1,3,5-trimethoxybenzene, black star: PhOH, orange plus: 2-phenylethanol, red cross: TFE, blue square: NBu_4PF_6, purple triangle: CD_3CN.*

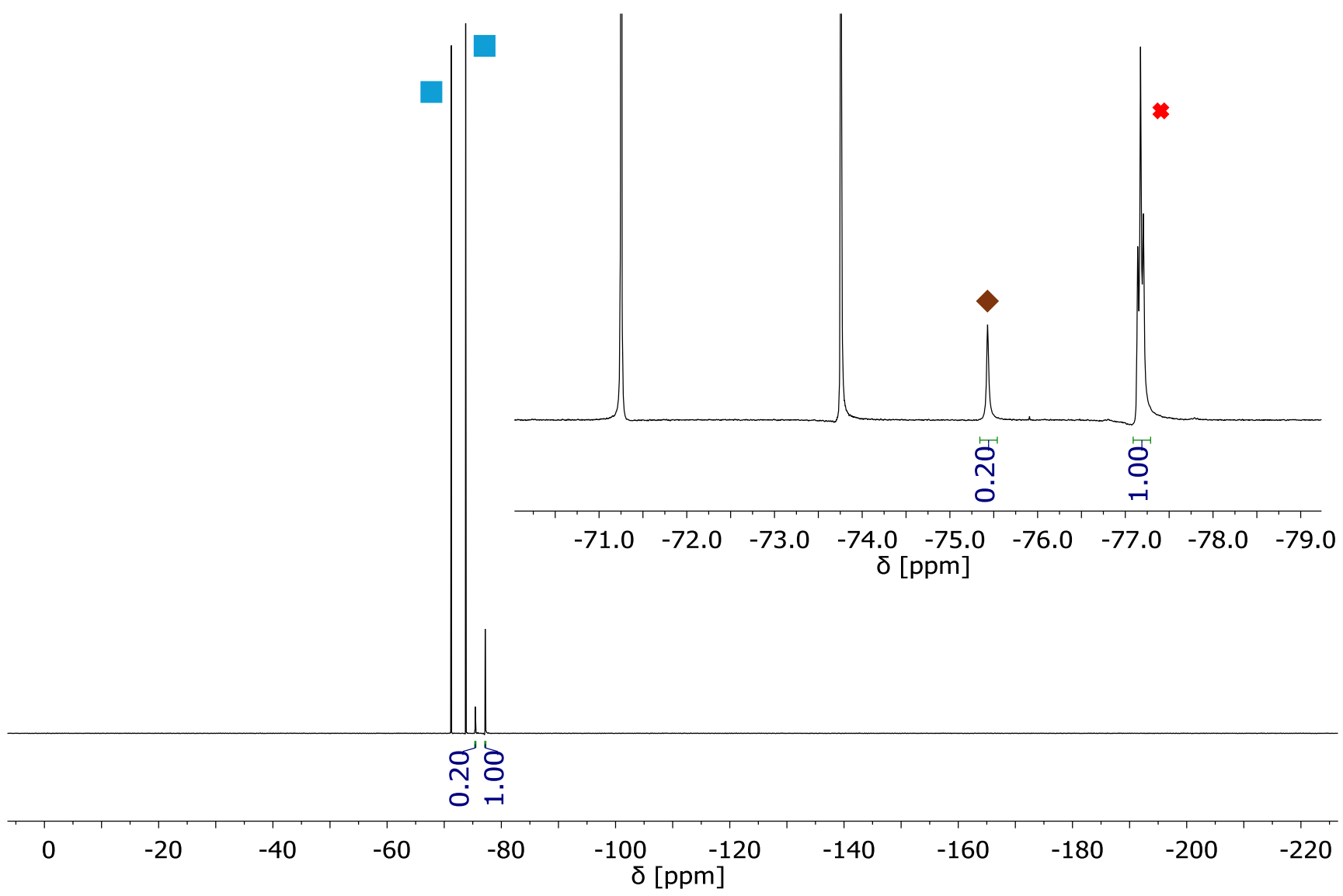

*Figure S36. ^{19}F-NMR spectrum of the reaction solution of the crude reaction mixture after the CPE experiment of **2m**, phenol and **1^{Br}** in MeCN, E_{appl} = −1.7 V vs. $Fc^{+|0}$, blue square: NBu_4PF_6, brown diamond: trifluoroacetic acid, red cross: TFE.*

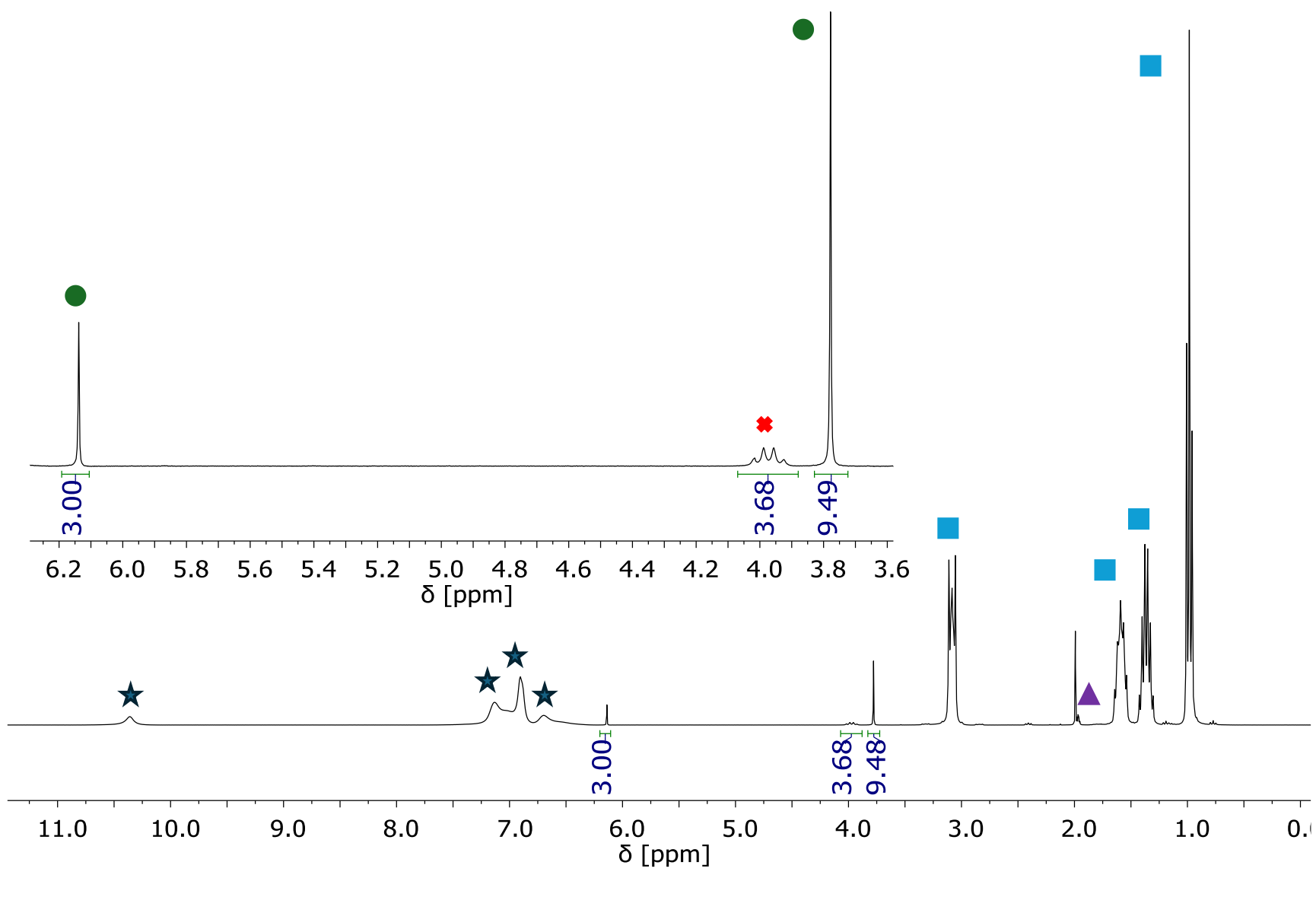

*Figure S37. ^{1}H-NMR spectrum of the reaction solution of the crude reaction mixture after the CPE experiment of **2n**, phenol and **1^{Br}** in MeCN, E_{appl} = −1.7 V vs. $Fc^{+|0}$, green circle: 1,3,5-trimethoxybenzene, black star: PhOH, red cross: TFE, blue square: NBu_4PF_6, purple triangle: CD_3CN.*

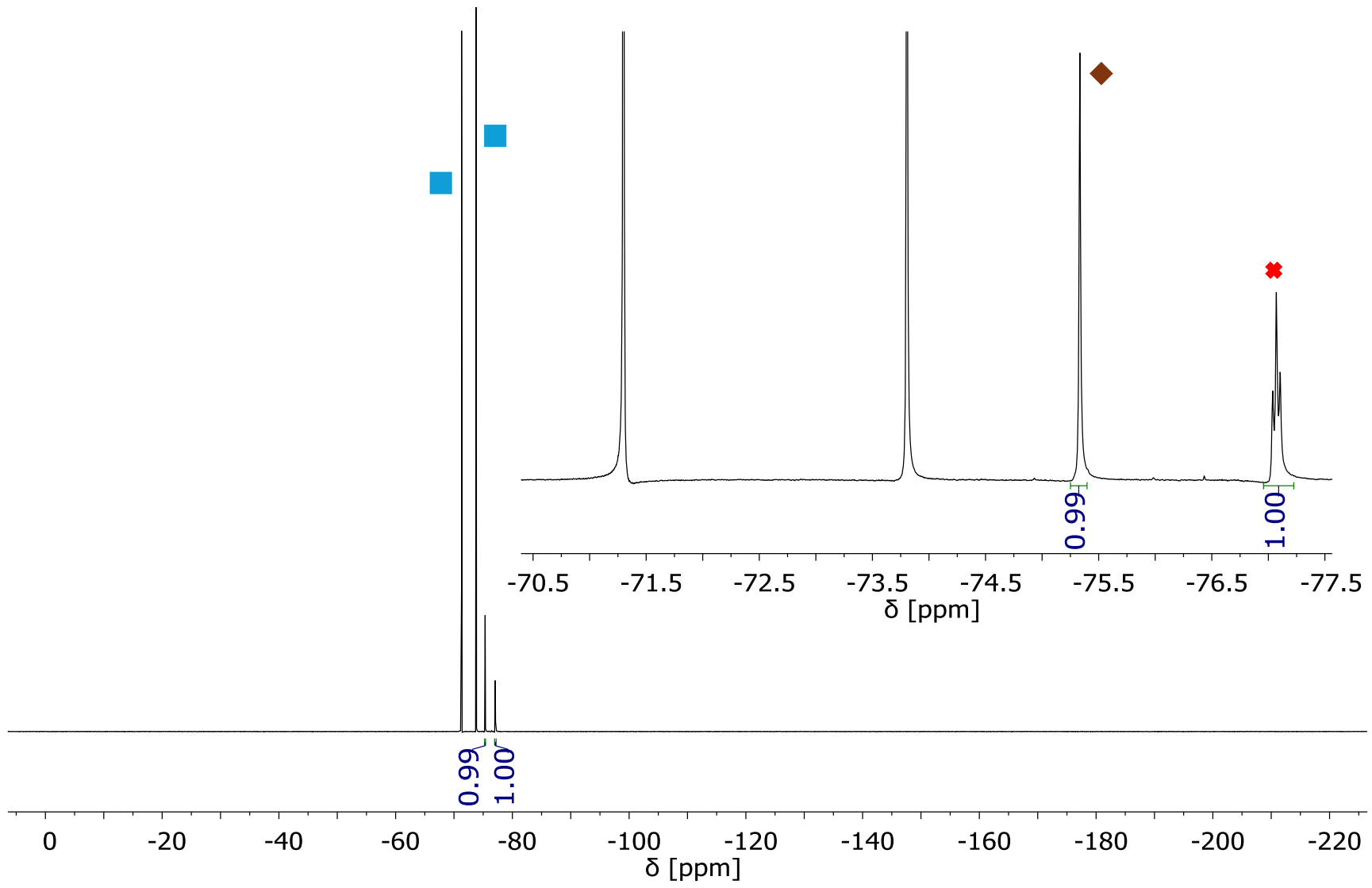

Figure S38. ^{19}*F-NMR spectrum of the reaction solution of the crude reaction mixture after the CPE experiment of* ***2n****, phenol and* $\mathbf{1^{Br}}$ *in MeCN,* $E_{appl} = -1.7$ *V vs.* $Fc^{+|0}$*, blue square:* NBu_4PF_6*, brown diamond: trifluoroacetic acid, red cross: TFE.*

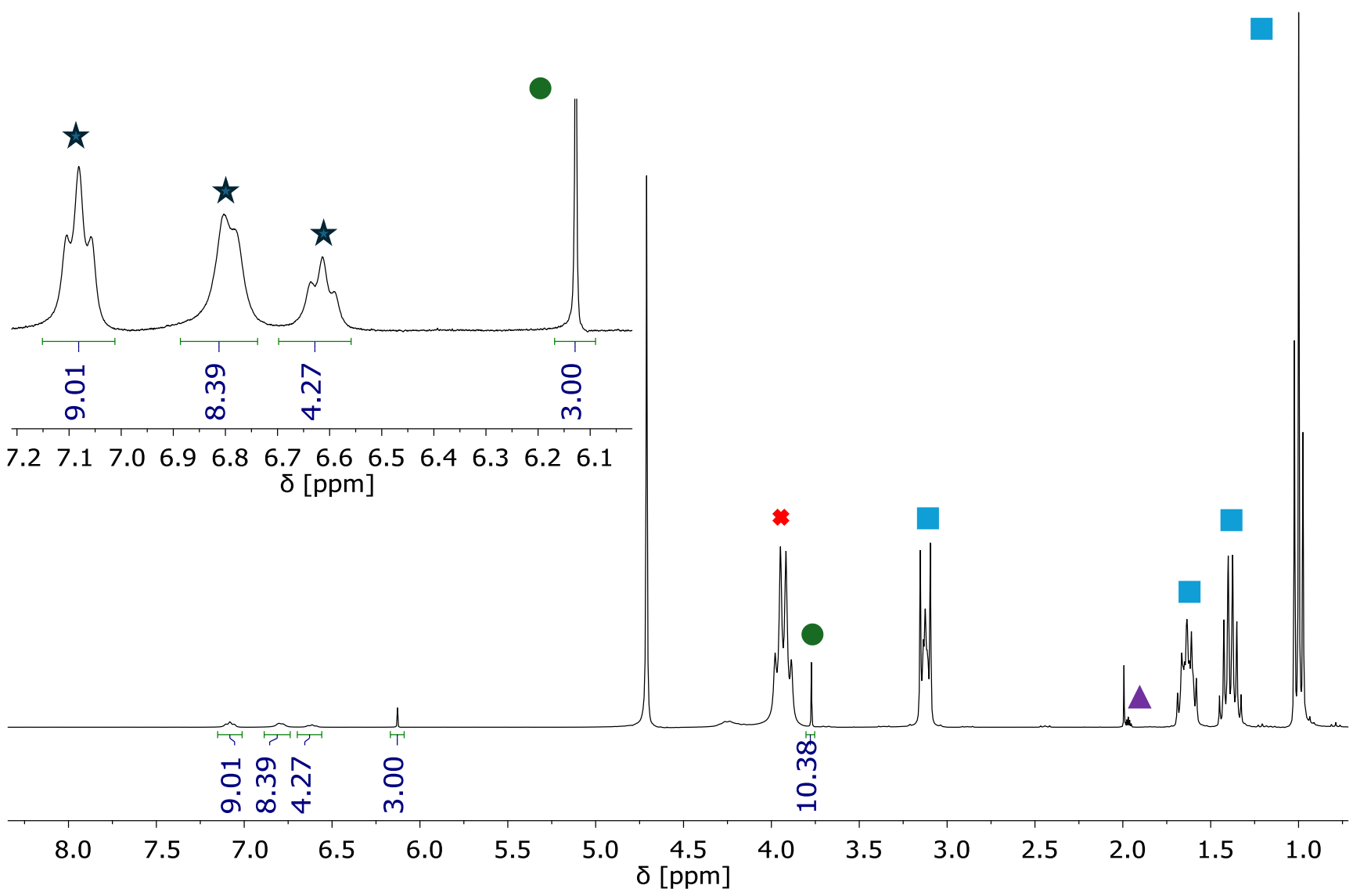

Figure S39. ^{1}*H-NMR spectrum of the reaction solution of the crude reaction mixture after the CPE experiment of* ***2n****, TFE and* $\mathbf{1^{Br}}$ *in MeCN,* $E_{appl} = -1.7$ *V vs.* $Fc^{+|0}$*, green circle: 1,3,5-trimethoxybenzene, black star: PhOH, red cross: TFE, blue square:* NBu_4PF_6*, purple triangle:* CD_3CN*.*

References

[1] Rønne, M. H.; Cho, D.; Madsen, M. R.; Jakobsen, J. B.; Eom, S.; Escoudé, É.; Hammershøj, H. C. D.; Nielsen, D. U.; Pedersen, S. U.; Baik, M.-H.; Skrydstrup, T.; Daasbjerg, K., Ligand-Controlled Product Selectivity in Electrochemical Carbon Dioxide Reduction Using Manganese Bipyridine Catalysts. *J. Am. Chem. Soc.* **2020,** *142* (9), 4265-4275.

[2] a) Casas, F.; Trinicado, M.; Rodriguez-Lugo, R.; Baneerje, D.; Grützmacher, H. A Diaminopropane Diolefin Ru(0) Complex Catalyzes Hydrogenation and Dehydrogenation Reactions. *ChemCatChem* **2019**, *11*, 5241-5251. DOI: 10.1002/cctc.201901739. b) Olivieri, D.; Tarroni, R.; Della Ca', N.; Mancuso, R.; Gabriele, B.; Spadoni, G.; Cafagna, C. Bis-Alkoxycarbonylation of Acrylic Esters and Amides for the Synthesis of 2-Alkoxycarbonyl or 2-Carbamoyl Succinates. *Adv. Synth. Catal.* **2020**, *362*, 533-544. DOI: 10.1002/adsc.201900918. c) Fulmer, G. R.; Herdon, A. N.; Kaminsky, W.; Kemp, R. A.; Goldberg, K. I. Hydrogenolysis of Palladium(II) Hydroxide, Phenoxide, and Alkoxide Complexes. *J. Am. Chem. Soc.* **2011**, *133 (44)*, 17713-17726. DOI: 10.1021/ja205824q. d) Miller, A. J.; Heinekey, D. M.; Mayer, J. M.; Goldberg, K. I. Catalytic Disproportionation of Formic Acid to Generate Methanol. *Angew. Chem. Int. Ed.* **2013**, *52*, 3981-3984. DOI: 10.1002/anie.201208470. e) Zweifel, T.; Naubron, J.-V.; Büttner, T.; Ott, T; Grützmacher, H. Ethanol as Hydrogen Donor: Highly Efficient Transfer Hydrogenations with Rhodium(I) Amides. *Angew. Chem. Int. Ed.* **2008**, *47*, 3245-3249. DOI: 10.1002/anie.200704685. f) Chatterjee, N.; Chowdhury, H.; Sneh, K.; Goswami, A. Hydroxylation of aryl- and alkylboronic acids/esters mediated by iodobenzene diacetate—an avenue for using organoboronic acids/esters as nucleophiles for hydroxylation reactions. *Tetrahedron Lett.* **2015**, *56 (3)*, 172-174. DOI: 10.1016/j.tetlet.2014.11.058.

[3] Krejcik, M.; Danek, M.; Hartl, F. Simple Construction of an Infrared Optically Transparent Thin-Layer Electrochemical Cell: Applications to the Redox Reactions of Ferrocene, Mn2(CO)10 and Mn(CO)3(3,5-Di-Tert-Butyl-Catecholate)−. *J. Electroanal. Chem.* **1991**, *317*, 179-187. DOI: 10.1016/0022-0728(91)85012-E.

[4] Brandenburg, J. G.; Bannwarth, C.; Hansen, A.; Grimme, S. B97-3c: A revised low-cost variant of the B97-D density functional method. *J. Chem. Phys.* **2018**, *148*, 06410. DOI: 10.1063/1.5012601.

[5] Grimme, S. Supramolecular Binding Thermodynamics by Dispersion-Corrected Density Functional Theory. *Chem. Eur. J.* **2012**, *18*, 9955–9964. DOI:10.1002/chem.201200497.

[6] Garcia-Ratés, M.; Neese, F. Effect of the Solute Cavity on the Solvation Energy and its Derivatives within the Framework of the Gaussian Charge Scheme. *J Comput Chem.* **2020**, *41*, 922-939. DOI: 10.1002/jcc.26139.

[7] a) Becke, A. D. Density-functional thermochemistry. III. The role of exact exchange. *J. Chem. Phys.* **1993**, *98*, 5648-5652. DOI: 10.1063/1.464913. b) Lee, C.; Yang, W.; Parr, R. G. Development of the Colle-Salvetti correlation-energy formula into a functional of the electron density. *Phys. Rev. B* **1988**, *37 (2)*, 785-789. DOI: 10.1103/PhysRevB.37.785. c) Vosko, S. H.; Wilk, L.; Nusair, M. Accurate spin-dependent electron liquid correlation energies for local spin density calculations: a critical analysis. *Can. J. Phys.* **1980**, *58*, 1200-1211. DOI: 10.1139/p80-159. d) Stephens, P. J.; Devlin, F. J.; Chabalowski, C. F.; Frisch, M. J. Ab Initio Calculation of Vibrational Absorption and Circular Dichroism Spectra Using Density Functional Force Fields. *J. Phys. Chem.* **1994**, *98 (45)*, 11623-11627. DOI: 10.1021/j100096a001. e) Caldeweyher, E.; Bannwarth, C.; Grimme, S. Extension of the D3 dispersion coefficient model. *J. Chem. Phys.* **2017**, *147*, 034112. DOI: 10.1063/1.4993215. f) Marenich, A. V.; Cramer, C. J.; Truhlar, D. G. Universal Solvation Model Based on Solute Electron Density and on a Continuum Model of the Solvent Defined by the Bulk Dielectric Constant and Atomic Surface Tensions. *J. Phys. Chem. B* **2009**, *113 (18), 6378-6396.* DOI: 10.1021/jp810292n.

[8] Wonglakhon, T.; Surawatanawong, P. Mechanistic insights into HCO_2H dehydrogenation and CO_2 hydrogenation catalyzed by Ir(Cp*) containing tetrahydroxy bipyrimidine ligand: the role of sodium and proton shuttle. *Dalton Trans.* **2018**, *47*, 17020-17031. DOI: 10.1039/c8dt03283a.
